# Genetic alterations driving metastatic colony formation are acquired outside of the primary tumour in melanoma

**DOI:** 10.1038/s41467-017-02674-y

**Published:** 2018-02-09

**Authors:** Melanie Werner-Klein, Sebastian Scheitler, Martin Hoffmann, Isabelle Hodak, Klaus Dietz, Petra Lehnert, Veronika Naimer, Bernhard Polzer, Steffi Treitschke, Christian Werno, Aleksandra Markiewicz, Kathrin Weidele, Zbigniew Czyz, Ulrich Hohenleutner, Christian Hafner, Sebastian Haferkamp, Mark Berneburg, Petra Rümmele, Anja Ulmer, Christoph A. Klein

**Affiliations:** 1 Experimental Medicine and Therapy Research, University of Regensburg, Franz-Josef Strauß Allee 11, 93053 Regensburg, Germany; 2 Regensburg Center for Interventional Immunology (RCI) and University Medical Center of Regensburg, Franz-Josef Strauß Allee 11, 93053 Regensburg, Germany; 3 Division of Personalized Tumor Therapy, Fraunhofer-Institute for Toxicology and Experimental Medicine, Regensburg, Am Biopark 9, 93053 Germany; 4 Department of Medical Biometry, University of Tübingen, Silcherstr. 5, 72076 Tübingen, Germany; 5 Department of Dermatology, University of Tübingen, Liebermeisterstr. 25, 72076 Tübingen, Germany; 6 Department of Dermatology, University of Regensburg, Franz-Josef Strauß Allee 11, 93053 Regensburg, Germany; 7 Department of Pathology, University of Regensburg, Franz-Josef Strauß Allee 11, 93053 Regensburg, Germany; 8 Institute of Pathology, University Hospital, Friedrich-Alexander-University Erlangen-Nürnberg, Krankenhausstr. 8-10, 91054 Erlangen, Germany

## Abstract

Mouse models indicate that metastatic dissemination occurs extremely early; however, the timing in human cancers is unknown. We therefore determined the time point of metastatic seeding relative to tumour thickness and genomic alterations in melanoma. Here, we find that lymphatic dissemination occurs shortly after dermal invasion of the primary lesion at a median thickness of ~0.5 mm and that typical driver changes, including *BRAF* mutation and gained or lost regions comprising genes like *MET* or *CDKNA2*, are acquired within the lymph node at the time of colony formation. These changes define a colonisation signature that was linked to xenograft formation in immunodeficient mice and death from melanoma. Thus, melanoma cells leave primary tumours early and evolve at different sites in parallel. We propose a model of metastatic melanoma dormancy, evolution and colonisation that will inform direct monitoring of adjuvant therapy targets.

## Abstract (web summary)

For several cancer types, mouse models indicate that metastasis occurs early. Here, the authors show that human melanoma cells disseminate early and illustrate a genetic colonisation signature that evolves in parallel at different sites.

## Introduction

The classical linear progression model^1^ holds that metastases are generated from late-disseminating clones that genetically resemble the predominant clone of the primary tumour (PT)^2,3^, whereas parallel progression suggests dissemination from early transformed lesions and acquisition of important genetic changes at the metastatic site^1^. Parallel progression gained substantial support by patient data^4,5^ and transgenic mouse models^6–8^, indicating that metastatic dissemination often occurs early. In mouse models, molecular mechanisms of early dissemination were identified and evidence was provided for a superior metastatic potential of early disseminated cancer cells (DCCs) compared to late DCCs^9,10^. In patients, comparative sequencing of PTs and metastases revealed branched parallel progression in all cases tested, whereas no case followed linear progression^11^. However, since clinical practice often derives therapy decisions from an assumed (based on a linear progression model) identity between accessible PTs and inaccessible metastatic seeds, a correct understanding of metastatic progression is essential. Surprisingly, despite high relevance for adjuvant therapies, which need to take into account the molecular heterogeneity of DCCs and PTs^12^, no study has ever investigated the relative time point of dissemination during tumour progression or assessed the evolutionary events during the early steps of metastasis, i.e., early colonisation, within target organs of patients.

In this study, we address both aspects in melanoma and lymph node metastasis because (i) overtly visible melanoma is often detected when tumour thickness is measured in micrometres, unlike late-detected cancers whose diameter is recorded in the range of centimetres and (ii) we recently developed a highly sensitive assay based on disaggregation and immunocytology to detect disseminated cancer cells in the sentinel lymph node (SLN). We demonstrated in more than 1000 patients^13^ that this method outperforms conventional histopathology and that gp100 is best suited (considering sensitivity and specificity) to detect melanoma cells^13,14^. We now apply this assay to detect, isolate and characterise early metastatic seeds and their molecular progression. We find that dissemination occurs very early and that early DCCs need to acquire important genetic alterations outside the PT to form metastatic colonies in patients or in xenografts. Moreover, we identify for each DCC alteration the most likely site of its acquisition.

## Results

### Tumour thickness at dissemination and colony formation

To determine melanoma thickness at seeding and colonisation, we examined the clinical data from our previous studies^13,14^, where we had established and validated a gp100-based detection method for single melanoma cells in SLNs of 1027 patients with clinically node-negative disease as assessed by palpation and ultrasound. Of these, 51% harboured gp100-positive cells^13^, whereas we did not detect a single gp100-positive cell among 70 control samples. Genetic analysis showed that 98% of randomly selected gp100-positive cells from SLNs harboured DNA sequence changes^13^.

**Fig. 1:**
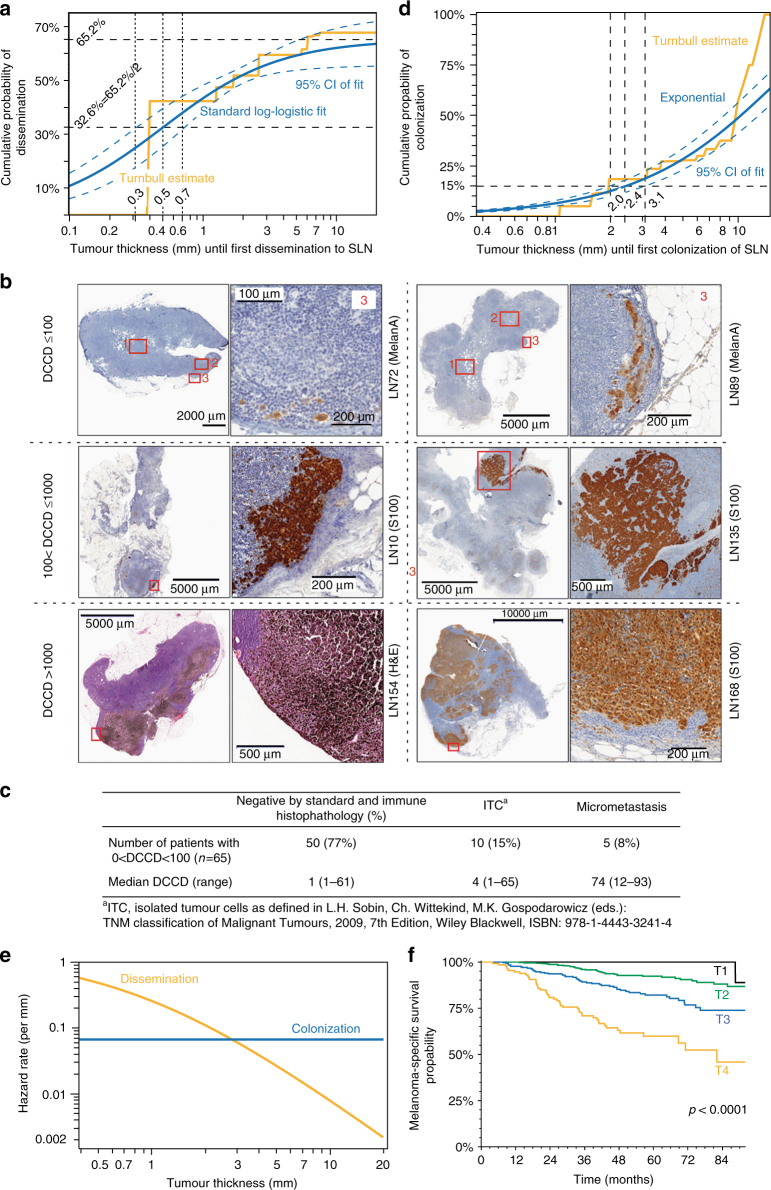
Dissemination of melanoma cells as function of tumour thickness. **a** Yellow function: estimated cumulative probability of dissemination as a function of tumour thickness (Turnbull) (*n* = 1027 patients). Blue line: Standard log-logistic distribution incorporating a fraction of patients without long-term dissemination (95% CI: blue dashed lines). Upper black dashed line: only 65.2% of melanomas disseminate lymphatically (95% CI: 60.4–70.0%). Fifty percent of this value (32.6%) provides the median thickness (0.5 mm, 95% CI: 0.3–0.7 mm) of disseminating melanomas (lower black dashed line). **b** Comparative analysis of histopathological and immuncytological SLN halves. Displayed are representative examples with immunocytological scores of DCCD ≤ 100, 100 < DCCD ≤ 1000 and DCCD > 1000. Samples LN 72 and LN 89 are stained against melan A LN 10, LN 135 and LN 168 against S100. LN 154 shows a highly pigmented melanoma in H&E staining. A close-up of the subcapsular region 3 is shown in the main figure; more central or core regions 1 and 2, see Supplementary Fig. 4a for higher magnification. **c** Evaluation of histopathological findings in corresponding sections of samples with a positive DCCD < 100. **d** Percentage of DCC-positive patients (*n* = 525) with colonisation (DCCD > 100) according to the Turnbull estimate (yellow). The percentage of colonisation (blue curve, 95% CI blue dashed curves) is described by a cumulative exponential distribution function (median 10.3 mm; 95% CI: 8.4–13.0 mm). **e** Hazard functions for dissemination (yellow line), and colonisation (blue line) describing the instantaneous risk per unit thickness for an event (dissemination, *n* = 1027; colonisation, *n* = 525) for those tumours, for which it has not yet occurred (de novo dissemination/ colonisation). **f** Survival analysis of melanoma patients (*n* = 1027) according to T stage (T1: ≤ 1 mm (*n* = 83); T2: 1.01–2.0 mm (*n* = 496); T3: 2.01–4.0 mm (*n* = 315); T4: >4 mm thickness (*n* = 133); log-rank test, *p* < 0.001)

There was a weak positive correlation (Spearman’s *ρ* = 0.18, *p* < 0.0001, *n* = 1027) between PT thickness and disseminated cancer cell density (DCCD; defined as the number of gp100-positive cells per million cells in disaggregated SLNs). The percentage of patients with gp100-positive SLNs increased only marginally from T1 (≤1 mm) to T4 (>4 mm) tumours (T1: 45.8%, T2: 47.4%, T3: 54.9% and T4: 59.4%), suggesting that dissemination occurs preferentially early. We used the nonparametric Turnbull method to determine thickness at dissemination and compared five different parametric models for best fit. While all models largely concurred (Table 1a), a standard log-logistic model outperformed the other approaches (Table 1a). These analyses revealed that (i) lymphatic dissemination was restricted to 65.2% of all patients and that (ii) in 50% of these cases cancer cells had spread before tumours reached a thickness of 0.5 mm (95% CI: 0.3–0.7 mm; Fig. 1a). In sum, our data show that ~1/3 of all melanomas disseminated lymphatically at a tumour thickness of <0.5 mm, ~1/3 at a thickness ≥0.5 mm and ~1/3 were not capable of lymphatic spread.

**Table 1:** Identification of best fitting model for tumour thickness at dissemination and colonisation

(a) Tumour thickness at dissemination								
Distribution	Asymptote (%)	Asymptote 95% CI	FPA (%)	TD at FPA (mm)	95% CI FPA (mm)	No of parameters	*p* value^a^	BIC^b^
Standard log-logistic	65.2	60.4–70.0	32.6	0.5	0.3–0.7	2	0.671	718.7
Exponential	58.1	55.2–61.0	29.0	0.5	0.4–0.7	2	0.604	719.5
Weibull	63.5	53.5–73.4	31.7	0.4	0.04–0.8	3	0.581	725.5
Log-normal	65.9	51.5–80.2	32.9	0.5	0.04–0.8	3	0.572	725.6
Fréchet	71.2	47.5–94.9	35.6	0.6	0.05–0.9	3	0.567	725.7

(b) Tumour thickness at colonisation							
Distribution	TD15 colonisation (mm)	95% CI TD15 (mm)	TD50 Colonisation (mm)	95% CI TD50 (mm)	No of parameters	*p* value^a^	BIC^b^
Exponential	2.4	2.0–3.1	10.3	8.4–13.0	1	0.31	210.1
Standard log-logistic	2.4	1.9–3.1	13.4	10.5–17.1	1	0.28	210.8
Fréchet	2.3	1.9–2.8	11.1	7.3–21.4	2	0.32	215.4
Log-normal	2.4	2.0–2.9	9.7	6.9–17.0	2	0.25	215.6
Weibull	2.5	2.0–3.1	8.9	6.8–14.2	2	0.18	216.0

*FPA* 50% of asymptote, *TD* tumour thickness, *TD15* tumour thickness at 15% colonisation, *TD50* tumour thickness at 50% colonisation, *CI* confidence interval, *BIC* Bayes information criterion

^a^For goodness of fit comparing model predictions with observed values (see methods)

^b^Lowest BIC identifies best model

We asked at what tumour thickness DCCs would have grown to a colony in SLNs. In all cases, we split the SLNs and analysed one half by histopathology (preserving the architecture) and the other by gp100 immunocytology after disaggregation (destroying the architecture but enabling quantification; for details, see ref. ^13^). To establish a DCCD representative for colonisation, as opposed to early dissemination (first arrival), we compared both halves of a patient’s SLN. Analysing histopathological detection rates vs. DCCD for 494 patients^14^, we had noted before that histopathological detection of cancer cells was associated with a DCCD of 100 in 50% of cases (see ref. ^14^). We therefore analysed 65 unselected, consecutive cases with a positive DCCD of up to 100, expecting that first colonies may become detectable slightly below this value. We assessed whether the SLN half screened by standard and immune histopathology contained so called isolated tumour cells (ITC; <0.2 mm or <200 cells) or larger lesions, i.e., micrometastases (Fig. 1b and Supplementary Fig. 4a). Of the 65 cases, 50 (77%) were negative by standard and immune histopathology, 10 (15%) harboured ITC and 5 (8%) micrometastases. Interestingly, the median DCCD of histopathologically negative and of ITC samples were 1 and 4, respectively, whereas micrometastatic lesions displayed a median DCCD of 74 in the half analysed by immunocytology (Fig. 1c). Thus, it seems safe to conclude that colonisation, understood as micrometastasis formation, occurs at a DCCD of ~100, whereas samples with lower DCCD comprise melanoma cells before colony outgrowth.

Of the 525 DCC-positive patients, the proportion of samples with a DCCD > 100 increased with tumour thickness. An exponential distribution function provided best fit (Fig. 1d and Table 1b). Median thickness at colonisation (10.3 mm) was about 20 times higher than at seeding. We noted that the risk of de novo tumour seeding steadily decreased as tumours grew, while the risk of de novo colonisation did not depend on tumour thickness (Fig. 1e)

### Discrepancy between dissemination and survival

Patients with T1 and T4 stage melanomas differed by only 13.6% for gp100-positive SLNs (38/83 (45.8%) vs. 79/133 (59.4%), respectively). To explore how this marginal difference in DCC positivity is linked to survival, we asked how many patients had died during the median follow-up period of 49 months (range, 3–123 months), with 370 (36%) patients having a follow-up of ≥5 years. Only 1/83 (1.2%) DCC-positive T1 stage melanoma patient died, consistent with previous studies^15,16^. In contrast, 47/133 (35.3%) of T4 stage cases harbouring DCCs died (Fig. 1f; 9-year survival 88.9% for T1, and 45.9% for T4; *p* < 0.0001, log-rank test). Thus, there is a discrepancy between T1 and T4 stage melanomas regarding seeding and death. Nevertheless, for the total cohort of patients, each DCC increased the risk of death^13^.

### Genetic lineages of PTs and DCCs

The standard approach to addressing outcome-associated differences between T1 and T4 melanomas employs PT tissue. It assumes that the molecular characteristics of metastasis-initiating DCCs can be identified using the PT, because PTs and DCCs are thought to be largely identical. To test this assumption, we compared the genomic profiles of PTs and their matched DCCs from SLNs.

**Fig. 2:**
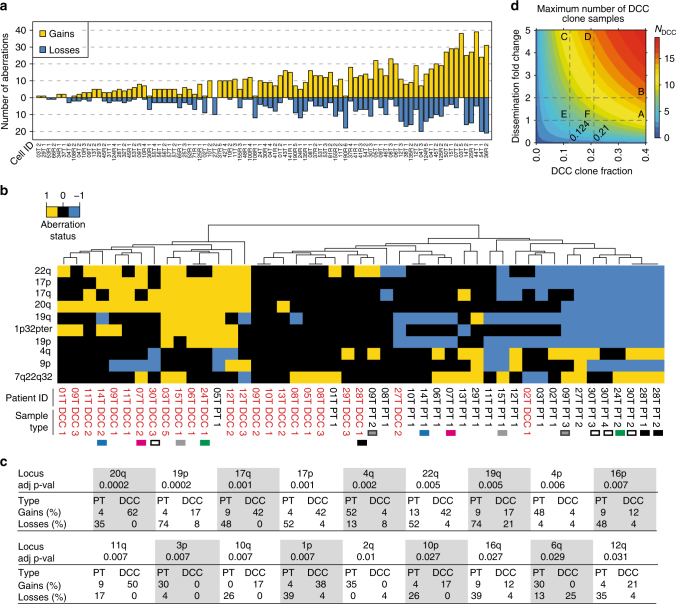
gp100-positive cells from SLNs and primary tumours display multiple copy number alterations (CNAs). **a** Ninety-one DCCs from our patients were selected according to QC criteria (see main text and Supplementary Figure 1) for CGH and mutation analysis. Histograms depict the genomic gains (yellow) or losses (blue) per cell. The identifiers indicate cell ID. **b** Cluster analysis of paired PTs (*n* = 23 samples) and DCCs (*n* = 24 samples) from 19 patients for chromosomal aberrations (gain = +1; loss = −1). Only the 10 most variable regions are included (see Supplementary Figure 5 for all 53 variable regions). Side-line identifiers indicate patient ID, sample type (PT and DCC) and sample index; bottom-line labels indicate chromosomal regions. Black (28T), white (30T) and dark grey (09T) filled squares indicate examples of PT-DCC pairs for which several areas of the PT were available. Examples of paired DCCs and PTs with varying PT thickness are indicated by coloured squares (green: 1 mm, grey: 1.4 mm, pink: 2 mm, blue: 7 mm PT thickness). **c** Comparison of paired PTs and DCCs for chromosomal aberrations. Displayed are the 18 chromosomal regions that differ significantly (FDR-adjusted Fisher’s exact test *p* value ≤0.05) between paired PTs (*n* = 23, 19 patients) and DCCs (*n* = 24, *n* = 19 patients) regarding aberration frequency. Gains and losses are given in percent. **d** Maximum number of DCC samples *N*_DCC_ (colour-coded) that cannot be excluded to derive from corresponding DCC-like clones within the PT plotted against the assumed DCC-like clone fraction in the PT and the successful dissemination fold change. If DCC-like and non-DCC-like PT clones disseminate equally (fold = 1), at most 11/24 DCC samples may result from DCC clones within the PT at the maximum indicated clone fraction of 0.40 (A); for twofold dissemination not more than 14/24 (B). For fold = 5, the same number of samples can be excluded up to a fraction of 0.124 (11/24) (C) and 0.21 (14/24) (D), respectively. These intermediate fractions correspond to a maximum of 5/24 (0.124) (E) and 7/24 (0.21) (F) DCC samples, respectively, if clones disseminate similarly (fold = 1). See 'Methods' section for details. Significance level *α* = 0.05 (Poisson-binomial distribution)

We first assessed the genomes of disseminated melanoma cells in a subset of 61 patients (Supplementary Fig. 1 and Supplementary Table 1) of which high-quality genomic DNA of DCCs was available for molecular analysis (in total, 91 individual DCCs). Inclusion criteria are summarised in Supplementary Fig. 1. The malignant origin of each included DCC was confirmed by comparative genomic hybridization (CGH; Fig. 2a) or targeted sequencing for *BRAF*/*NRAS*. DCCs were found to display a large range of copy number variations ranging from 1 to 52 per cell (Fig. 2a; median = 14; interquartile range = 14.8). We noted that genomic gains per cell (median = 9; range, 0–39) were more frequent than losses (median 3.5; range, 0–21). As a control, we isolated 30 single leukocytes and performed CGH analysis (Supplementary Fig. 2) with none of the control cells displaying any aberration (*p* < 0.0001; Fisher’s exact test).

Next, DNA from available PTs of these patients was isolated by laser microdissection (Supplementary Fig. 4b) and whenever possible several areas were analysed. However, compared to other cancers such as kidney cancer^17^, early stage melanomas are very small, often technically precluding the assessment of subclones from different areas. Microdissected PT samples (*n* = 23, 19 patients) and micromanipulator-isolated single DCCs (*n* = 24, 19 patients) were analysed by CGH (Supplementary Fig. 3). Regardless of melanoma thickness, there was a striking disparity between PTs and matched DCCs (Fig. 2b). Unexpectedly, PTs from different individuals clustered closer together than individual PTs and their matched DCCs (Fig. 2b and Supplementary Fig. 5). Interestingly, PTs displayed  significantly more deletions than DCCs (Fig. 2b, c and Supplementary Fig. 5; *p* = 0.003, Mann–Whitney *U* test). The corresponding difference in gains was clearly nonsignificant (*p* = 0.66, Mann–Whitney *U* test). When several areas from the same PTs were available, we noted genomic heterogeneity, however they still clustered together (for example 28T, 30T and 09T in Fig. 2b and Supplementary Fig. 5) apart from their paired DCCs.

The observed disparity between PTs and DCCs may result from two scenarios: (i) the isolated DCCs represent hidden subclones within the PT that still exist at the time of surgery and from which dissemination may have occurred late (scenario 1) or (ii) the DCCs disseminated early and represent genomic states that have become extinct within the primary site by later selective sweeps (scenario 2). We investigated the 19 matched patient samples assuming that each DCC derives from one of the PT areas selected according to highest similarity (i.e., maximum support for scenario 1; see 'Methods' section for details). For defining a detection threshold, we supposed that an aberration must be present in at least 60% of cells in the corresponding PT sample analysed by CGH^18^. To obtain a conservative estimate of how many of our DCC samples could derive from DCC-identical subclones within the PT, i.e., conform to scenario 1, we derived maximum DCC subclone percentages that are still consistent with the respective PT bulk measurement results for all loci. Depending on the individual patient, these maximum percentages ranged between 10 and 40%. We then calculated the maximum number of DCCs that could derive from the hidden clones on an *α* = 0.05 significance level. We found that a maximum of 11/24 DCC samples could possibly result from DCC-identical subclones within the PT if their corresponding subclone fractions are indeed maximal, i.e., equal 10–40% depending on the individual patient (Fig. 2d). In case of rarer DCC subclones, one must assume that they disseminate several fold more successfully than the remaining (i.e., non-DCC like) PT clones. For example, if a DCC subclone displays a fivefold higher successful dissemination propensity, it may not exceed a frequency of 12.4% for the same maximum of 11/24 subclone-derived DCCs. Consequently, SLN DCCs, displaying the identified karyotypes, may equally likely result from both scenarios, i.e. late dissemination from rare PT subclones (scenario 1) or and early dissemination (scenario 2) followed by selective extinction or somatic progression of the disseminating clone. However, if the DCC-like subclones indeed make up less than the respectively estimated maxima of 10–40% of PT cells and display a dissemination ability similar to the predominant (non-DCC-like) PT clones, the late dissemination scenario 1 becomes increasingly unlikely.

**Fig. 3:**
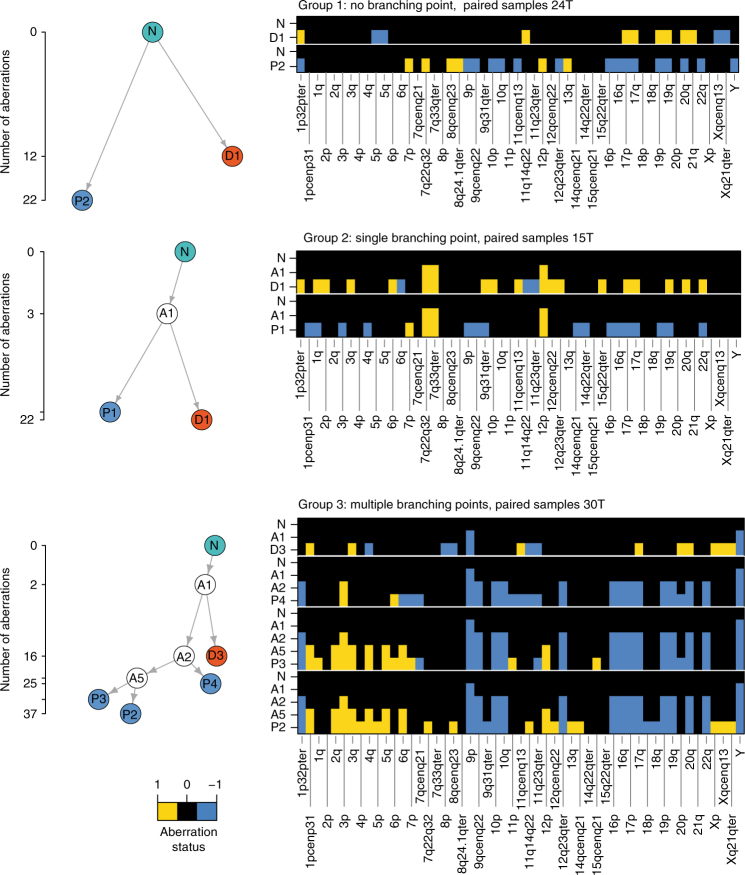
Phylogenetic trees of paired PT and DCC samples. Representative examples for the three distinct groups observed in our data (for all 19 patients, see Supplementary Figures 6–8). The ordinate of the tree panels indicates the number of aberrations per CGH profile (square root scale). Heat maps show aberration profiles along paths from normal cells (N) to PT (P1–4) and DCC (D1, D3) samples, respectively. Profiles A1, A2 and A5 denote inferred common ancestors (intermediates) (see 'Methods' section)

We then reconstructed the phylogeny of PTs and their matched DCCs based on the assumption that (1) aberrations more common among samples have developed earlier in time and (2) the overall number of genomic changes leading to the observed sample data is minimal (maximum parsimony). Applying this approach to our copy number alteration (CNA) data, we observed three types of phylogenetic trees (Fig. 3) defined by the number of branching points. In all 19 matched samples, DCCs and PT samples branched early and evolved in parallel (Fig. 3 and Supplementary Figs. 6–8).

### DCCs and PTs are rarely clonal for BRAF mutations

**Fig. 4:**
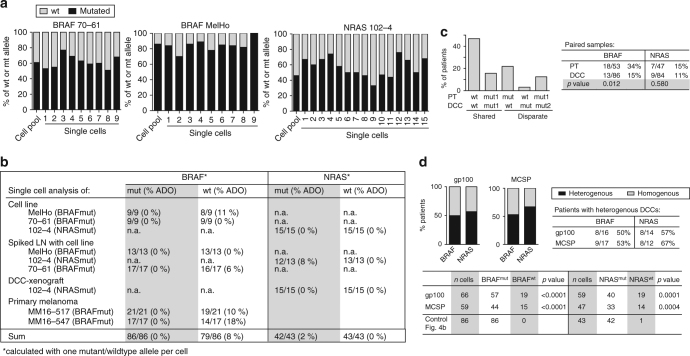
BRAF and NRAS mutations in PTs and DCCs. **a** Single cell WGA reliably captures wild-type and mutated alleles. Exon 15 mutation c1799T>A (*BRAF*) and Exon 2 mutation c181C>A (*NRAS*) were detected in all single cells (lanes 1–9 or 1–15) of cell lines with *BRAF* (cell lines 70–61 (*n* = 9) and MelHo (*n* = 9)) or *NRAS* (cell line 102–4; *n* = 15) mutation. The allelic ratio of wt vs. mut alleles of each cell line was determined using pooled DNA. Note that this ratio is preserved in most single cells. **b** Summary of results in **a** and detection of *BRAF* and *NRAS* mutation in single gp100+ cells isolated from (i) lymph nodes from healthy controls spiked with melanoma cell line cells, processed and stained as SLN of melanoma patients; (ii) an enzymatically digested DCC-xenograft derived from NRAS-mutated DCCs and (iii) primary melanomas with BRAF mutation. **c** Mutation analysis of *BRAF* and *NRAS* for paired PT-DCC samples (*n* = 32 patients). Different mutations (either *NRAS* or *BRAF*) are indicated by mut1 and mut2. Fisher’s exact test *p* values indicate differences in *BRAF* mutational status between PTs and DCCs. **d** Percentage of patients (*n* = see Table 1) with homogeneous (all cells harbouring the mutation) and heterogeneous *BRAF*/*NRAS* mutational status among DCCs. DCCs were detected using two markers, gp100 (*n* = 43 cells) or MCSP (*n* = 61 cells)

Since *BRAF* and *NRAS* mutations are frequent in melanoma, occurring in 40 and 21% of cases on average, respectively^19^, we investigated whether PTs pass them on to DCCs. We performed a series of control experiments on single cells from cell lines, xenografts and PTs to determine the allelic drop-out (ADO) rate for mutant and wild-type alleles. We assumed one mutant and one wild-type allele per cell and found the ADO rate for *BRAF* to range between 0% (mutant allele; 86/86 alleles detected) and 8% (wt allele; 79/86 alleles detected; Fig. 4a, b) and for *NRAS* between 0% (wt allele; 43/43 detected) and 2% (mutant allele; 42/43 detected). We concluded that our single-cell assay is well suited to address questions of clonality and lineage descent for *BRAF* and *NRAS*. In matched patient samples, *BRAF* was mutated more frequently in PTs (34%) than in DCCs (15%; *p* = 0.012, Fisher’s exact test; *n* = 32 patients; Fig. 4c and Supplementary Fig. 11), whereas no significant difference was observed for *NRAS* mutations (15% in PTs and 11% in DCCs; *p* = 0.58; *n* = 29 patients). For these two oncogenes, we found a shared wild type in 47%, a shared mutated status in 16% and disparate mutational states in 37% of cases (Fig. 4c). Among patients with mutated PTs, matched DCCs were mostly not sharing these mutations (shared in 3/11 for *BRAF* and 3/6 for *NRAS*, Supplemental Fig. 12), indicating that they had disseminated before fixation of the mutation within the primary site.

*BRAF* and *NRAS* mutations have been suggested to initiate melanoma^20^ and, consequently, be fully clonal. We therefore determined if sibling DCCs, i.e., individual DCCs from patients with *BRAF* or *NRAS* mutant gp100+ DCCs of whom we could isolate more than one DCC, all share the same mutation. We found that sibling gp100+ DCCs are heterogeneous in 50 and 57% of cases for *BRAF* and *NRAS* mutations, respectively (Fig. 4d). To rule out a selective effect of the detection marker gp100, we analysed additional melanoma DCCs detected by the melanoma marker MCSP (melanoma-associated chondroitin sulphate proteoglycan) and obtained similar results (Fig. 4d). To test whether this heterogeneity of sibling DCCs reflects method-induced mutant allele loss or non-clonality, we compared the mutation rates of sibling DCCs with single cells (*n* = 86 for *BRAF* and 43 for *NRAS*) from our control experiments (Fig. 4b). In the latter, we detected the mutant allele in all (*BRAF*) or all but one (*NRAS*) single cells, whereas the mutant allele often remained undetected in gp100^+^ or MCSP^+^ DCCs (*p* < 0.0001 for *BRAF*; *p* = 0.0001 and 0.0004 for *NRAS* and gp100 and MCSP, respectively; Fig. 4d). Therefore, the frequent failure to detect the mutant allele in sibling DCCs cannot be explained by a technical artefact. We conclude that *BRAF* or *NRAS* mutations among sibling DCCs are not fully clonal. Finally, when we compared the mutational state for PT-DCC-metastases triplets or pairs of PTs-metastases or pairs of DCC-metastases, we found that DCCs with and without *BRAF*/*NRAS* mutations were able to form manifest metastases (Supplementary Fig. 12a, b).

In summary, both copy number alterations and targeted mutation analysis demonstrate that primary melanomas and their paired DCCs are largely genetically disparate implying early evolutionary branching. Consequently, we focused on the evolution of DCCs within the SLN because of their obvious relevance for systemic cancer progression.

### Genomic evolution of DCCs outside the PT

DCCs disseminate early and display genomes either representative of earlier evolutionary states before loss of chromosomal material (Fig. 2b, c) or of a hidden PT subclone. To address cancer evolution outside the PT, we first investigated the genomic profiles of DCCs that had not yet expanded within the SLN and representing the time early after arrival.

**Fig. 5:**
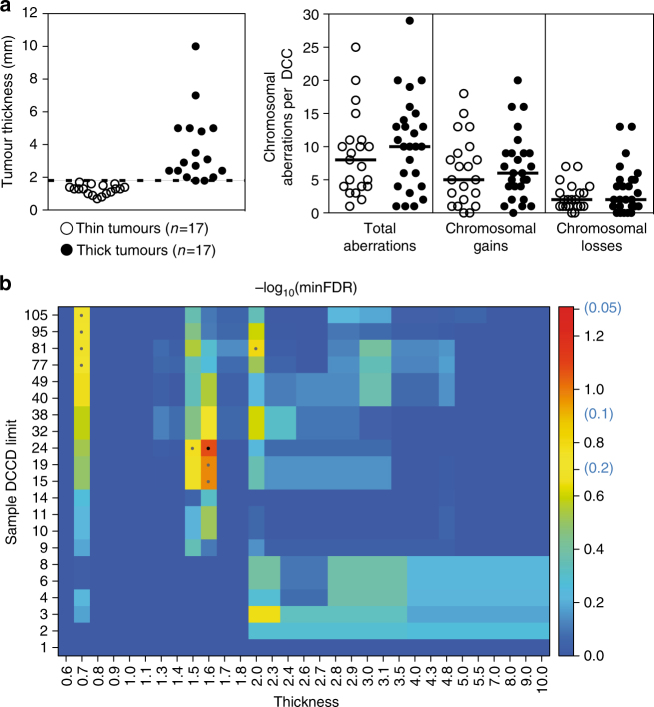
Molecular analysis of DCCs at dissemination. **a** Left: Separation of primary melanomas according to median thickness into thin (<1.8 mm) and thick (≥1.8 mm) tumours, from which DCCs were analysed. Right: Number of chromosomal aberrations per DCC from thin (*n* = 17) and thick (n = 17) tumours . **b** Samples were selected for suggested early arrival in the SLN as indicated by small DCCD values up to 105 and then tested for genomic differences regarding their origin from thick and thin primaries. The *y* axis specifies the DCCD limit up to which samples were included in the analysis, with sample numbers ranging from 6 (DCCD = 1) over 31 (DCCD = 24) to 46 (DCCD = 105). The *x* axis reports the limiting thickness according to which samples were split regarding their association with thin or thick tumours (set points left of these values). Colour indicates the minimum FDR-adjusted *p* values (Fisher’s exact test) across loci/mutations assessing the largest genomic differences between these two groups. The smallest *p* value of FDR = 0.084 occurs at DCCD = 24 and thickness = 1.6 mm for locus 18q21–23 (black central dot). *p* values up to FDR = 0.2 are indicated by grey dots and may refer to the loci 18q21–23, 1q24–44 or 8q23–24.3. The colour key includes the non-log-transformed FDR values 0.05, 0.1 and 0.2 in brackets. No significant differences were observed

Since colony formation became clearly apparent at a DCCD > 100 (Fig. 1b and ref. ^14^), we considered samples with a DCCD ranging from 1 to 99 to contain early arriving DCCs. Thus, DCCs from the 34 patients with a DCCD below 100 were categorised as thin and thick melanomas taking the median thickness as a split (Fig. 5a). We found no difference in the number of CNAs (total; gains; losses) between DCCs arriving from thick and thin melanomas (Fig. 5a) and could not identify any genetic alteration that was significantly enriched in DCCs from thick melanomas (Fig. 5b). Therefore, we can exclude a simple model of mutation accumulation and late arising metastasis-causing alterations within growing PTs.

**Fig. 6:**
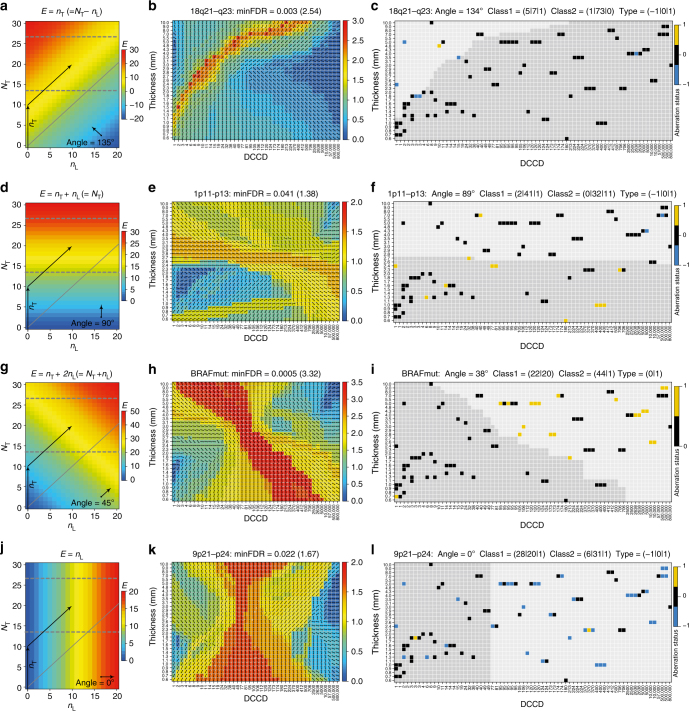
DCCs acquire genetic alterations within and outside the PT. **a**,** d**,** g**,** j** Genome alterations are explained by four different risk scenarios. Panels **a**, **d**, **g** and **j** show different risk functions *E* (colour-coded) as a function of the number of cell divisions in the SLN (*n*_L_) and the PT (*N*_T_). The two arrows indicate a prototypic state trajectory: initially, cell divisions exclusively occur within the PT (*N*_T_ > 0, *n*_L_ = 0) until after *n*_*T*_ cell divisions a tumour cell disseminates to the SLN. Subsequently, PT and SLN cells grow simultaneously (*N*_T_ > 0, *n*_L_ > 0). The grey dashed horizontal lines at *N*_T_ = 14.4 and 26.6 correspond to the minimum (0.6 mm) and maximum (10 mm) experimental thickness values ('Methods' section). Growth rates are presumed equal in both environments implying (i) inaccessibility of the region below the diagonal (grey solid line), consistent with the empty lower right triangular area in **c**, **f**, **i**, **l** and (ii) *N*_T_ = *n*_T_ + *n*_L_ (after dissemination; 'Methods' section). The angle indicates the direction of the normal vector regarding equal risk lines. **b**,** e**,** h**,** k** Classification results for four prototypic CGH results (loci/mutations) using linear classifiers with 2D set points according to the indicated DCCD and PT thickness values and directions as pictured by the small arrows within each rectangle of the colour matrix ('Methods' section). Colour encodes the negative decadic logarithm of the FDR-corrected *p* values of each classification (Fisher’s exact test). Basically, the red/orange areas separate regions differing in their distribution of genomic alterations. Their orientation parallels the equal risk lines in **a**, **d**, **g**, **j**. DCCD and thickness values were chosen according to experiment while classifier set points were slightly displaced relative to these values (white points included for the 5% most significant FDR values). Minimum FDR values are indicated by black points. *N* = 87 DCCs, 57 patients, 82 loci/mutations. **c**,** f**,** i**,** l** Corresponding measurement results as well as the class assignments of the best (lowest FDR) classifiers indicated by the light (class1) and dark (class2) grey areas. The class boundary does not necessarily appear linear as samples are listed according to rank while linear classification was performed in log(DCCD)-log(thickness) space. The classifier angle and the distribution of deletions (−1), balances/wt (0) and amplifications/mutations (+1) in each class are given in the title

**Fig. 7:**
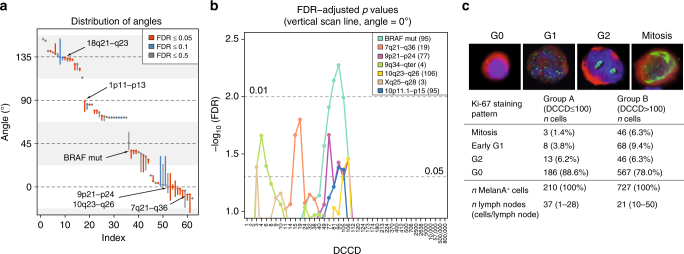
DCC genetic alterations acquired in the SLN correspond to colonisation. **a** Distribution of best classifier angles for different loci/mutations. These can be grouped according to the prototypic categories in **a**, **d**, **g** and **j**. Vertical bars correspond to the range of angles associated with the same minimal FDR value, diamonds indicate angles that are closest to the ideal prototypes. Only the 62/82 loci/mutions with at least 10 samples per class were included. **b** DCCD-dependence of genomic changes derived using a vertical classifier (angle = 0°) positioned according to the experimentally measured values (set points left of these values). Only category 3 and 4 loci whose FDR values fall below 0.05 are shown. Numbers in brackets following the legend (e.g. BRAF mut (95)) indicate peak DCCD. **c** Proliferation of DCCs in sentinel nodes. Immunofluorescence of Melan A^+^ cells in G0-, G1-, G2-phase and mitosis (from left to right). Nucleus (blue), Melan A (red), Ki-67 (green; for *n*, see figure; Fisher’s exact test, *p* = 0.0005 for G0 *vs*. non-G0)

We investigated for all genetic changes of SLN DCCs their association to PT thickness and DCCD assuming that alterations may either be acquired within the PT before dissemination or thereafter. Since the risk of acquiring genetic changes is linked to the number of cell divisions and the local environment, we determined for each alteration whether the risk depends on the number of cell divisions within the PT or the SLN. Strikingly, we could classify most of the genetic changes into one of four categories associated with the angle of a straight line that best separates DCC samples with different aberration status in the two-dimensional space of thickness and DCCD (Figs. 6a, d, g, j and 7a; and 'Methods' section). The risk of acquiring category 1 changes (Fig. 6a–c) is solely related to the number of cell divisions a cell undergoes within the PT before dissemination (*n*_T_) while subsequent cell divisions within the SLN (*n*_L_) do not alter (increase or decrease) their emergence (Fig. 6a–c). Notably, all changes acquired under the linear progression model conform to this category. Category 2 (Fig. 6d–f) comprises alterations whose risk is fully explained by the total number of cell divisions, i.e., the generation of changes is independent of the microenvironment and reflects total disease duration if growth rates within and outside the primary are equal. As an example, initial gain of chromosome 1p11–13 was 're-normalised' over time and vanished as PTs grew larger than 2.7 mm (Fig. 6e, f). Category 3 changes (Fig. 6g–i) have a higher risk of emergence within the SLN than within the PT. A striking example here is mutant *BRAF*, which is rarely found in very small primary lesions^21,22^, but is highly associated with the emergence of SLN colonies (Fig. 6h, i). Thus, although BRAF mutations are present in ~1/3 of PTs in paired PT-DCC samples (Fig. 4c), BRAF-mutated PT cells obviously do either not disseminate or not engraft in SLNs. Finally, we identified alterations, whose risk was negligible for the number of cell divisions within the PT before dissemination but dramatically increased at colonisation of the distant site (category 4, Fig. 6j–l).

In contrast to category 1 (exclusive dependence on the number of cell divisions before dissemination) and category 2 (dependence on the total number of divisions), categories 3 and 4 show a real and observable dependence on the number of divisions within the SLN, i.e., the DCCD. To identify critical DCCD values, we scanned for the emergence of DCCD-dependent genomic changes. Of note, alterations in regions harbouring typical melanoma-driving onco- and tumour suppressor genes such as *BRAF* or *CDKN2A* (9p21-p24) emerged at a DCCD of 77–106, exactly when we noted morphological colony formation (Fig. 7b and Supplementary Tables 2 and 3). NRAS mutation was associated with insignificant classification FDR values and the best classifier for NRAS separated out only 4 out of 87 samples. In Figure 7a we required a minimum of 10 samples per class and displayed in Figure 7b only loci reaching 5% significance. Thus, NRAS does not surface in either graphics. Obviously, cell divisions within the SLN determine the risk of acquiring alterations. Therefore, we compared labelling indices for the proliferation marker Ki-67 in SLN DCCs from patients with DCCD ≤ 100 and DCCD > 100, i.e., before and after colony formation (Fig. 7c). As double staining of MIB-1 (anti-Ki-67) and HMB45 (anti-gp100) was unsuccessful, we replaced gp100 with the melanoma-associated marker Melan A^14^. Ki-67 expression was assessed in 37 nodes with DCCD ≤ 100 (Group A) and 21 nodes with DCCD > 100 (Group B), involving evaluation of 937 cells in total. In Group A, 11.4% of cells were clearly cycling , while 22.0% were in the cell cycle in group B (*p* = 0.0005; Fisher’s exact test, Fig. 7c). Thus, a basal proliferation rate may initially enable acquisition of genetic alterations that subsequently drive accelerated outgrowth.

### Colonisation-associated alterations and malignant behaviour

**Fig. 8:**
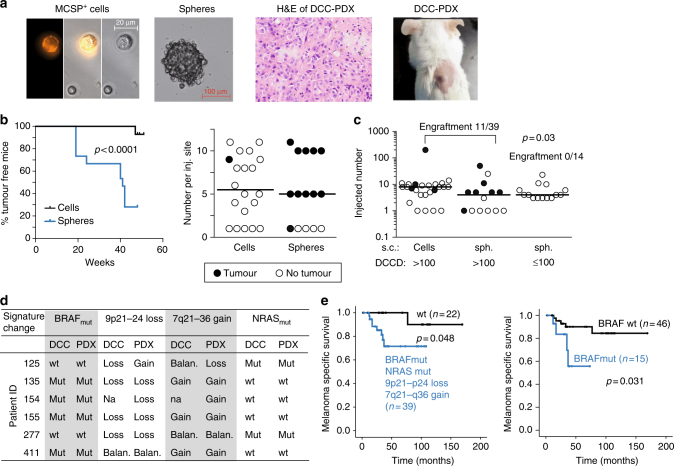
Tumour-forming ability of DCCs before and after colonisation and patient survival. **a** Left to right: Isolated MCSP^+^ DCCs from a patient-SLN; DCC-derived sphere; H&E staining of a patient DCC-derived xenograft (DCC-PDX); DCC-PDX (7 s.c. injected DCCs). Pictures are representative for 15 transplanted patient samples. **b** Side-by-side transplantation of paired MCSP^+^ DCCs (*n* = 3 patients, 20 injection sites) and DCC-derived spheres (*n* = 3 patients, 15 injection sites) from the same patient into NSG mice. Left: Kaplan–Meier analysis of tumour-free mice (*p* < 0.0001, log-rank test). Right: number of injected MCSP^+^ DCCs and DCC-derived spheres per injection site. Black filled circles indicate tumour formation. **c** Number of MCSP^+^ DCCs (DCCD > 100: *n* = 25 injection sites) or DCC-derived spheres (DCCD > 100: *n* = 14 injection sites; DCCD ≤ 100: *n* = 14 injection sites) that were transplanted into NSG mice. Each circle represents one injection site. Black filled circles indicate tumour formation (engraftment). The *p* value (*p* = 0.03 Fisher’s exact test) indicates a significant difference in the engraftment rate for samples with DCCD > 100 (cells and spheres were pooled, *n* = 39, *n* = 10 patients) vs. DCCD ≤ 100 (spheres, *n* = 14, *n* = 5 patients). **d** Mutational status of patient-derived DCCs and their respective xenografts regarding the colonisation signature from Fig. 7a and Supplementary Table 3 (i.e., loss of genetic locus 9p21–24 and gain of 7q21–36, *BRAF*mut and *NRAS*mut), *n* = 6 patients. Note that samples 125 and 277 add *NRAS* mutations to the colonisation signature. **e** Left: Kaplan–Meier survival analysis of patients with DCCs that display at least one of the final colonisation signature features (i.e., loss of genetic locus 9p21–24, gain of 7q21–36, *BRAF*mut or *NRAS*mut, *n* = 39) or not (wt regarding colonisation signature, *n* = 22). Right: Kaplan–Meier survival analysis of patients with DCCs that display *BRAF* mutation (*BRAF*mut, *n* = 15) or wild type sequence (wt, *n* = 46)

Cancer cells forming a SLN colony displayed a characteristic signature of alterations. To test whether pre- and post-colonising DCCs have tumour-initiating ability, we transplanted both DCC types and cells from cell lines into NSG mice. We first evaluated conditions for xenotransplantation of few melanoma cells^23^. For cell line cells (Supplementary Fig. 13a, b) and patient DCCs (Fig. 8a, b), we compared two approaches: direct transplantation of groups of DCCs and transplantation of DCC spheres after brief culture under melanosphere conditions (Fig. 8a). We found that melanospheres formed tumours in immunodeficient NSG mice earlier and more frequently than groups of single cells (*p* < 0.0001, log-rank test; Fig. 8b and Supplementary Fig. 13a, b). The applied conditions supported growth from as few as a single transplanted sphere or a group of seven DCCs (Fig. 8b). We therefore only transplanted spheres from samples with DCCD ≤ 100 and spheres or groups of single DCCs from samples with DCCD > 100 for comparing the tumour-initiating ability of DCCs from SLNs with DCCD ≤ 100 to those with DCCD > 100. The number of spheres per injected site was similar in both cases (*p* = 0.39, Mann–Whitney *U* test; Fig. 8c). Strikingly, a DCCD > 100 was predictive of successful xenotransplantation (11/39 (28.2%) transplantations gave rise to tumours in 6/10 patients), whereas samples with DCCD ≤ 100 never established tumours (0/14 injection sites in 0/5 patients; Fig. 8c; *p*= 0.03, Fisher's exact test). Genetic fingerprinting confirmed patient origin (Supplementary Fig. 13c). Furthermore, in 5/6 patient-derived xenografts either *BRAF* mutation, loss of 9p21–24 (harbouring *CDKN2A)*, or gain of 7q21–36 (harbouring *MET*) was present (Fig. 8d). In one case (patient 125 in Fig. 8d), successful outgrowth was linked to the presence of an *NRAS* mutation.

Finally, we explored whether the genetic aberrations associated with colony formation in the SLN or with tumour formation in mice were associated with clinical outcome. For this, we tested if one or several alterations of our final colonisation signature (comprising *BRAF* mutation, loss of 9p21–24, gain of 7q21–36 and *NRAS* mutation) in single DCCs increased the risk of death. Indeed, one or several of the signature changes were present in DCCs of 8/9 (89%) patients dying from melanoma (*p* = 0.048, log-rank test; Fig. 8e), with *BRAF* mutation being the most relevant single indicator (*p* = 0.031).

## Discussion

This study provides a compelling molecular model that accounts for the ectopic evolution in the spread of early systemic cancer. We report the first estimate for the tumour extent at which metastatic dissemination of a human cancer occurs; it is based on highly sensitive, direct detection of DCCs rather than being inferred from tumour growth rates^24–26^. The median thickness of seeding melanomas was 0.5 mm (95% CI: 0.3–0.7 mm), much smaller than previously thought.

We therefore specifically investigated the fate of early DCCs and collected all available evidence to differentiate between three possible scenarios. Model 1: early DCCs (here defined as DCCs before evidence of colony formation in the SLN) inherit all genetic changes from the primary lesion that enable them to form a metastatic colony. Model 2: early DCCs may exist but are irrelevant; only late DCCs that disseminate after acquisition of driver mutations within the PT, grow out to manifest colonies. Model 3: early DCCs are genetically immature and acquire critical alterations after homing to a distant site and thereby gain the ability to form a colony.

Our data allow us to reject model 1. Despite clear evidence for dissemination from earliest lesions, the 9-year death rate for T1 melanomas was much lower than the seeding rate at this stage. In contrast, seeding and death rates in T4 melanomas were similar. In line with this, pre-colonising DCCs lacked typical melanoma driver changes and were unable to form xenografts. Therefore, dissemination is necessary but not sufficient to generate lethal metastasis.

To address model 2, we first compared PTs and DCCs. We focused on CNAs because they best reflect cancerous progression as opposed to point mutations^4,20,27^, which are frequently detected also in benign lesions unlike CNAs^4,20,28^. Independent of tumour thickness, CNA profiles of PTs and matched DCCs were clearly different. Primary tumours displayed a differential loss of chromosomal material and phylogenetic analysis indicated that PTs and DCCs branch off early and progress in parallel, rendering model 2 unlikely. To corroborate this reasoning, we scrutinised DCCs that had just arrived in the SLN and specifically compared DCCs from thin vs. thick melanomas. We tested whether we can detect late-disseminating, genetically more mature, possibly more aggressive cells in SLNs from patients with thick melanomas. However, early DCCs from thin and thick melanomas did not differ characteristically, neither with regard to the number nor the quality of alterations.

Our analysis finally refuted model 2 and supported model 3 when we determined for each DCC alteration its link to the number of cell divisions within and outside the PT and visualised the aberration status as a function of tumour thickness and DCCD. Most changes were found to fall into one of four categories (category 1: exclusive dependence on the number of cell divisions before dissemination; category 2: dependence on the total number of cell divisions before and after dissemination; category 3: higher impact of cell divisions in SLN; category 4: exclusive dependence on the number of cell divisions after dissemination). Only 29% of loci showing significant FDR values correspond to category 1 and their alteration may thus derive from linear progression. The great majority (71%), however, conform to category 2 (18%), 3 (18%) or 4 (36%) that are in an increasing degree inconsistent with linear progression. Had, e.g., BRAF mutations been persistently generated within the primary and continuously spread to the SLN, a positive mutation status at small DCCD values would have been a frequent observation in Fig. 6i. Since, however, BRAF mutations existed in ~1/3 of PT samples while being absent in precolonizing DCCs, BRAF mutant cells either remain in the primary, are unable to survive in or bypass the sentinel. Recent findings support the first explanation because strong oncogene activation apparently favours proliferation, but impedes dissemination^10,21,29^. Consistently, most of our experimental data cannot be explained by the linear progression paradigm stating that genetic alterations originate exclusively from the primary. Instead, our alternative hypothesis that most alterations, including BRAF mutation, are preferentially generated within the SLN post dissemination (conforming to parallel progression) is in distinctly and visibly better agreement with our experimental observations. Interestingly, acquisition of category 3 and 4 changes was associated with DCCDs of 77–106, exactly when colony formation became morphologically evident. Thus, early DCCs arrive immaturely and progress within the SLN (model 3).

Our transplantation experiments strongly support this conclusion. Acquisition of these changes by DCCs was associated with xenograft formation in mice, whereas early DCCs (DCCD < 100) failed to form tumours, consistent with DCC immaturity. DCCD > 100 was linked to colony formation in the SLN and a significant increase in mean Ki-67 proliferation index from 11 to 22%, confirming basal proliferation and indicating the acquisition of advantageous changes. Interestingly, the mean proliferation index of 11% before colonisation resembles that of T1 melanomas at transition to the tumorigenic vertical growth phase (VGP), previously found to range between 9 and 13%^22^. Thus, early SLN DCCs display progression-enabling growth rates.

**Fig. 9:**
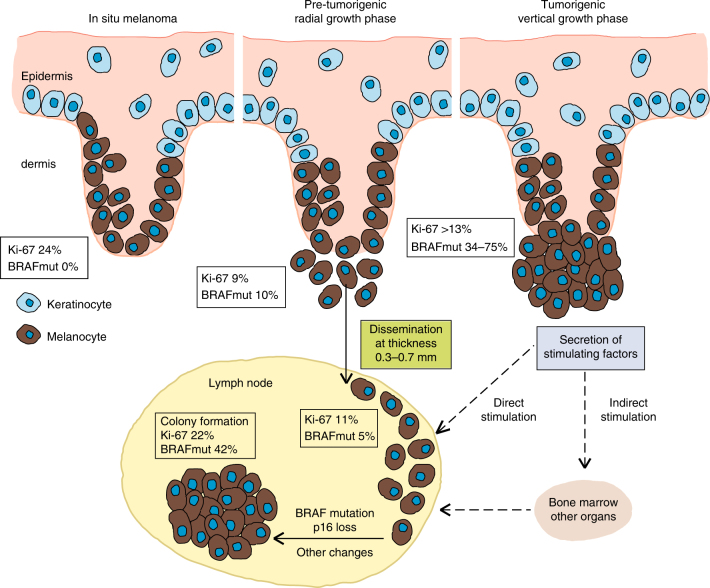
Model of melanoma progression from local to metastatic disease. Histologic appearance, patient-derived dissemination estimate, proliferation rate and *BRAF* mutational state are integrated into the scheme. Data are taken from this study and from references^22, 30, 31^

Our model (model 3) is fully supported by previous analyses. *BRAF* mutations were found in 0 and 10% of in situ and early radial growth phase (RGP) melanomas, respectively ^30,31^. When the VGP starts, melanomas expand in the dermis (i.e., become tumourigenic), often acquire *BRAF* mutations ^30,31^, and increase their proliferation rate^22^. DCCs in the SLN and possibly other metastatic sites apparently re-capitulate this process during colonisation. Our genomic data and mathematical analyses of dissemination and colonisation indicate that after PT cells acquired a proliferative phenotype, de novo dissemination becomes increasingly unlikely, as evidenced by hazard rates for de novo dissemination diminishing with increasing tumour thickness. Also, *BRAF* and *NRAS* mutations in PTs were rarely present in matched DCCs, indicating that *BRAF/NRAS* mutant clones were less likely to seed than to expand. All findings cohere to a model of a largely parallel passage through the 'Vogelgram'^32^ of melanoma cells at the primary and secondary sites (Fig. 9). The initial disparity between PTs and DCCs regarding *BRAF* mutations, in addition to the strong selective advantage this mutations endows DCCs with during colony formation, explains both the observed disparity for *BRAF* mutations between PTs and metastases^31,33–35^ and the increased frequency of *BRAF* mutations in metastases compared to early RGP melanomas.

Evolution outside the PT is consistent with a large sequencing study comparing PTs and matched metastasis from various cancers^11^, where linear progression was never observed. However, it should be noted that most cancer phylogeny models apply the 'infinite sites assumption'^36–38^, stating that each mutation is generated maximally once^39^, which is not justified in cancer. For example, melanoma of unrelated patients converge on the classical *BRAF*-*V600E* mutation in 40% of cases. Therefore, it is unreasonable to exclude *a priori* that two clones of the same cancer acquire the *BRAF*-*V600E* mutation independently. Our single-cell analysis supports previously reported evidence^40^ that current models of branching evolution as deduced from sequencing studies of bulk tumours still underestimate the complexity of cancer evolution by relying on the infinite sites model.

It remains to be explored in more detail why high T stage is a risk factor in melanoma. The correlation between tumour thickness and colonisation may suggest that PTs facilitate colonisation by secreted factors^41^. Such factors may act in a dose (i.e., tumour volume)-dependent manner, either directly upon DCCs or indirectly by altering the microenvironment locally or systemically. Formal proof of this scenario would require model systems that enable genomic in vitro progression from immature to mature cancer cells triggered by supporting factors. Such models are currently not available. The lack of adequate supporting signals from the PT may explain why we failed to observe engraftment of pre-colonising cells in NSG mice, that rarely thrive, neither in mice nor in humans, after early melanoma removal.

Our findings have implications for the development of adjuvant therapies. First, novel drugs may be required to eradicate the metastatic seed prior to colonisation, as pre-colonising DCCs may lack typical drug targets. Second, this also applies to the identification of neo-antigens in DCCs to develop adequate immune strategies against metachronous metastasis. Last but not least, we will need suitable assays to monitor molecular evolution during occult disease.

## Methods

### Patients

We used data from 1027 melanoma patients from Tübingen with clinically node negative (as assessed by palpation and ultrasound) melanoma who underwent tumour resection  and sentinel node biopsy to describe the association between thickness and melanoma spread^13^. Molecular studies and *BRAF*/*NRAS* mutational survival analysis included patients recruited in Tübingen and Regensburg. Informed written consent was obtained from all patients. The study was approved by the ethics committees of the Universities Tübingen (ethics vote number 5/99) and Regensburg (07-079).

### Cell lines

The melanoma cell lines A375 and MelHo were used (obtained from the Leibniz Institute DSMZ-German Collection of Microorganisms and Cell cultures) and their identity verified by short tandem repeat (STR) analysis (Cell-ID™, Promega). MelHo is listed in the ICLAC database for mis-identified cell lines due to unclear patient origin, but was used as the cell line is heterogenic for the exon 15 mutation c1799T>A (*BRAF*). The cell lines 70–61 and 102–4 were developed from DCC-derived xenografts and are exon 15 mutation c1799T>A (*BRAF*) and exon 3 mutation c181C>A (*NRAS*) mutated, respectively, as determined by Sanger sequencing (Sequiserve, Vaterstetten, Germany). Their patient origin was verified by STR analysis (Cell-ID™, Promega), their melanoma origin by a human pathologist and their aberrant genotype by CGH. A375 and MelHo were maintained in DMEM, 10% FCS, 100 U/ml penicillin, 0.1 mg/ml streptomycin; 102–4 and 70–61 in RPMI, 10% FCS, 100 U/ml penicillin, 0.1 mg/ml streptomycin. All cell lines were routinely tested for mycoplasma and were found to be negative.

### Controls

Control lymph nodes (*n* = 70) were obtained from 60 non-melanoma patients (47 skin-draining nodes from non-malignant conditions, 6 SLNs from non-melanoma skin cancer patients and 17 nodes from non-small cell lung cancer patients). Lymph nodes were halved and then either fixed (for histopathology) or disaggregated without fixation, stained and evaluated identically to the melanoma-derived SLNs. After screening 2 × 10^6^ lymphocytes, the control lymph node status was revealed to the observer, and screening of the samples was continued until completion, unlike to the melanoma patient samples.

### Lymph node disaggregation and immunocytology

Quantitative immunocytology was performed as described^13,14^ after SLN biopsy using unfixed SLN tissue. Briefly, the lymphatic tissue was cut into 1-mm pieces and disaggregated mechanically into a single-cell suspension by rotating knives (DAKO Medimachine, DAKO), washed with HBSS (Life Technologies, Heidelberg, Germany) and centrifuged on a density gradient made of a 60% Percoll solution (Amersham, Uppsala, Sweden). Cells were counted using a Neubauer counting chamber. Per slide, 10^6^ cells from the interphase were then given onto adhesion slides (Menzel, Braunschweig, Germany) in a volume of 1 ml PBS. After sedimentation for 1 h, the slides were air-dried overnight. Immunocytological staining was carried out with the alkaline phosphatase/anti-alkaline phosphatase method using primary antibodies against gp100 (HMB45, 0.7 µg/ml, catalogue number M0634, DAKO) and as primary antibody and 5-bromo-4-chloro-3-indolyl phosphate/NBT (DAKO) as substrate, yielding a blue reaction product. A SLN was defined as gp100 positive if it contained at least one gp100-positive cell. The number of positive cells per million lymphocytes was recorded. Positive samples were stored for a maximum of 4 days in PBS at 4 °C until cell isolation for whole-genome amplification. For the isolation of living DCCs, single cells were stained with an anti-human MCSP (melanoma chondroitin sulphate proteoglycan, clone 9.2.27, catalogue number 554275, BD Pharmingen, 25 µg/ml) according to the manufacturer's recommendations and detected by indirect immunofluorescence (goat anti-mouse-Cy3, catalogue number 115-166-071, Jackson, 15 µg/ml). After washing, MCSP^+^ cells were isolated using a micromanipulator (Eppendorf PatchMan NP2) and transplanted.

### Comparison between single cells and sphere transplantations

Single cells of disaggregated SLNs were plated in 6 cm poly-HEMA (12 mg/ml, Sigma-Aldrich)-coated cell culture plates (Sigma-Aldrich) at a density of 200,000 viable cells/ml. Cells were grown in a serum-free DMEM/Ham’s F12 basal medium (PAN Biotech GmbH), supplemented with 100 U/ml penicillin, 0.1 mg/ml streptomycin (both PAN Biotech GmbH), 0.5% BSA (VWR-Biochemical), 10 µg/ml insulin (Sigma-Aldrich), 10 nM HEPES (Sigma-Aldrich), 1 × B27 (Life Technology GmbH), 10 ng/ml EGF (Sigma-Aldrich) and 10 ng/ml bFGF (Sigma-Aldrich), 4 μg/ml heparin (Sigma-Aldrich), 5 ng/ml GRO-α (R&D Systems), 20 ng/ml HIL-6 (kindly provided by S. Rose-John) and 0.2% methylcellulose (Sigma-Aldrich). Cultures were incubated at 37 °C and 5% CO_2_ and 7% O_2_. Sphere growth was weekly monitored. To generate spheres from melanoma cell lines (MelHo, A375 maintained in DMEM, 10%FCS, 100 U/ml penicillin, 0.1 mg/ml streptomycin), single cells were plated at a density of 10,000 viable cells/ml on poly-HEMA (12 mg/ml)-coated cell culture plates in the same medium as for SLN cells, but without HIL-6 and GRO-α. Spheres were isolated manually.

### Xenotransplantation

Spheres or MCSP^+^ cells from disaggregated SLNs were collected using a micropipettor or micromanipulator and pooled in a microwell (volume 10–15 µl, Terasaki). Microwells were pre-coated over night with 12 mg/ml poly-HEMA (Sigma-Aldrich) at RT. Single cells were transplanted in a final volume of 30 µl and 25% high-concentration matrigel (BD Biosciences) as published before^23^. Cells were injected with an insulin syringe (Microfine, 29 G, U-50, BD Biosciences) subcutaneously into NOD.Cg-*Prkdc*^*scid*^ *IL2r*γ^*tmWjl/Sz*^ (NSG, 6–8 weeks old, males and females). Mice were purchased from the Jackson Laboratory and maintained under specific-pathogen-free conditions, with acidified water and food ad libitum in the research animal facilities of the University of Regensburg, Germany. All approved experimental animal procedures were conducted to German federal and state regulations (Government of Upper Palatinate or Lower Franconia). Mice were palpated every week at the site of injection. Melanoma origin of xenografts was verified by a human pathologist and patient origin was authenticated using STR analysis (Cell-ID™, Promega). Due to the whole-genome amplification (Klein et al., 1999) of samples prior to STR analysis, which includes restriction digest by Mse I, only the STR loci TH01, D21S11, D5S818, D13S317, D16S538 and vWA can be used for detection. Amplified fragments were detected using 3100-Avant Genetic Analyzer (Applied Biosystems). Fragment sizes were determined manually using Cell™ ID Allelic Ladder and Cell™ ID Bins 1.0 provided by Promega.

### Ki-67 labelling of DCC

For immunofluorescence staining, cells were incubated with primary antibodies against Melan A/MART-1 (clone A103, rabbit monoclonal dilution 1:100, 11 µg/ml, catalogue number 281M-86, Sigma) and Ki-67 (clone MIB-1, mouse monoclonal, catalogue number M7240, DAKO, 1.6 µg/ml) overnight at 4 °C. As secondary antibodies, we used Alexa Fluor 555 (donkey anti-rabbit, Thermo-Fisher-Scientific/Invitrogen, catalogue number A-31572) and Alexa Fluor 488 (donkey anti-mouse, Thermo-Fisher-Scientific/Invitrogen, catalogue number A-21202). The nucleus was stained with DAPI (blue), Melan A with Alexa Fluor 555 (red) and Ki-67 with Alexa Fluor 488 (green). Counterstaining was performed with 4′d-diamidino-2-phenylindole (DAPI) in mounting medium (Vector, Vectashield).

### DNA extraction and microdissection of PT areas

Areas for DNA extraction were marked by a pathologist (P.R.) and subsequently extracted from paraffin-embedded tumour blocks. The PALM microbeam system (Bernried) was used for microdissection and catapulting. DNA from microdissected areas was isolated as described in the section whole-genome amplification.

### Whole-genome amplification

Whole-genome amplification (WGA) from single cells or microdissected tissue DNA was performed as previously described and is described therein in detail^42,43^. The method is now commercially available as kit (*Ampli1*, Silicon Biosystems).

### Comparative genomic hybridisation

CGH of single cells and microdissected tissue was performed as previously described and is described therein in detail^42,44^. For most samples we used chromosomal CGH, as it is a very robust method, well established for single cells, and for some samples array CGH. We carefully compared both methods and found a good agreement between array CGH and chromosomal CGH when applied to same samples^44^. While aCGH may detect more changes (mainly for aberrations <10 Mb), the overall picture for aCGH and cCGH is very similar. For cases where we used aCGH, the resolution was adjusted to that of cCGH.

### Phylogeny of PTs and DCCs

For phylogenetic tree inference an independent method was developed because present methods are targeted either at two-valued mutation data^45,46^ or major–minor (allelic) copy numbers from SNP arrays or sequencing^47^. Briefly, phylogenetic trees were generated by assuming ideal (i.e., error free) data and inferring plausible common ancestors (intermediates) of aberration profiles in matched samples by extracting shared features of an increasing number of samples, i.e., evaluating common aberrations of sample pairs, triplets, quads, etc., and organising these ancestors according to hierarchical levels. Subsequently, admissible edges were constructed top–down between vertices allowing for at most two relosses of acquired gains and no regains of any losses (these conditions are also ensured globally for each path). Then, all simple paths from the normal cell to the samples were generated using the igraph R-package (version 1.0.1), combined into a directed acyclic graph (DAG) and filtered for fewest genomic changes along the graph and lowest number of intermediates (maximum parsimony). While different ‘equal length’ DAG solutions are generally possible using this approach, for the investigated samples all computations resulted in unique tree structures. For an illustration of the method and comparison to the infinite sites model, see Supplementary Figure 9.

### DCC sampling model

Basics: A DCC may derive from a minority clone of the PT that is overlooked by our CGH measurement for the bulk PT sample. Metaphase CGH requires 60% of cells in a sample to show an amplification (deletion) to be detected^18^. This corresponds to a limiting mean allele frequency of 2 + 0.6 (2–0.6). Thus, a maximum of 40% of cells may be balanced although an aberration has been detected for the whole PT sample. This percentage is reduced to 20% if the minority clone has an opposing aberration. The general problem statement is to find DCC-like PT clone fractions $f_k\left( {k = 1, \ldots ,M} \right)$, with *M* the number of measured DCCs, and non-DCC-like clone allele frequencies $C_i\left( {i = 1, \ldots ,L} \right)$, with *L* the number of measured loci, for which the composite mean allele frequency $\bar P_i = \left( {1 - \mathop {\sum }\nolimits_k f_k} \right)C_i + \mathop {\sum }\nolimits_k f_kD_{ki}$ falls within the interval range measured for locus *i* of the bulk PT sample, i.e., one of the intervals $I_1 = [1,1.4]$, $I_2 = (1.4,2.6)$ or $I_3 = [2.6,3]$ (Supplementary Figure 10). Here, we presuppose that single cells show heterozygous alterations only, corresponding to the DCC measurement data *D*_*ki*_ assuming values 1 (deleted), 2 (balanced) or 3 (amplified). Each non-DCC-like PT-clone allele frequency *C*_*i*_ can be the average of different (unmeasured) clones and thus may take any real value in the interval [1,3]. Notably, *f*_*k*_ = 0 for all *k* is always a solution since *C*_*i*_ can be freely chosen to match the measured interval range. If multiple vectors $\mathop{f}\limits^{\rightharpoonup} = \left( {f_1, \ldots ,f_M} \right)$ fulfill the above requirements we chose the one with the maximum product $\mathop {\prod }\nolimits_k f_k$ to obtain a conservative estimate for DCC origin from DCC-like PT clones (see also ‘Computation’ below). If multiple loci are present, valid vectors $\mathop{f}\limits^{\rightharpoonup}$ need to be determined across all loci prior to selection according to maximum product. The 19 patients for whom matched PT-DCC measurements were available comprised 23 PT and 24 DCC samples. Specifically, there were 13 patients with one PT and one DCC sample, i.e., $\left( {T,D} \right) = \left( {1,1} \right)$, and 3, 1, 1 and 1 patients with $\left( {T,D} \right) = \left( {1,2} \right),\left( {2,1} \right),\left( {3,1} \right)$ and (2,3), respectively. We assumed that each DCC derives from one of the matched PT samples and tested all *T*^*D*^ assignment possibilities per patient finally choosing the one with the maximum product of fractions (conservative estimate, see above). This resulted in 5, 9 and 10 DCCs with maximum clone fractions of $f_1 = 0.1$, $f_2 = 0.2$ and $f_4 = 0.4$, respectively. For Fig. 2d, DCC clone fractions were scaled per sample according to $f_i^s = f_i \cdot s (i = 1,2,4)$ with *s* running from 0 to 1 while the reference $x = f_4 \cdot s$ is displayed on the *x* axis, e.g. when $f_4^s$ for a sample with maximum fraction $f_{{\mathrm{max}}} = 0.4$ increases from 0 to 0.4, $f_1^s$ for a patient with $f_{{\mathrm{max}}} = 0.1$ rises from 0 to 0.1.

Differential dissemination: We assumed that different tumour cell clones can have different rates of successful dissemination and denoted by *d* the ratio (fold change) of DCC-like/non-DCC-like successful dissemination. Accordingly, the clonal fraction of the DCC with index *i* in the SLN is given by $p_i = {\rm{d}}f_i/\left( {1 + (d - 1)\mathop {\sum }\nolimits_k f_k} \right)$.

Computation: In case of only one unique value *p* for all *N* = 24 DCC samples, the probability of independently picking *n* DCCs derived from the corresponding DCC-like PT clone is given by the binomial probability $pr\left( n \right) = b\left( {N,n} \right)p^n\left( {1 - p} \right)^{N - n}$, with *b* the binomial coefficient. The minimum number of samples n* that can be excluded to derive from DCC-like clones according to significance level *α* = 0.05 can be calculated according to $1 - \mathop {\sum }\nolimits_{l < n^ \star } pr\left( l \right) \le \alpha$. The maximum number of samples that cannot be excluded on the same *α*-level is then one less. If different DCCs have different fractional values $p_k\left( {k = 1 \ldots M} \right)$, the probability for *n* successes is given by the Poisson-binomial distribution $pr\left( n \right) = \mathop {\sum}\nolimits_{A \in F(n)} {\mathop {\prod}\nolimits_{i \in A} {p_i} \mathop {\prod}\nolimits_{j \in A^C} {(1 - p_j)} }$, in which *F*(*n*) is the set of all subsets of *n* integers out of $\left\{ {1, \ldots ,M} \right\}$ and *A*^*C*^ is the complement of *A* regarding $\left\{ {1, \ldots ,M} \right\}$. Since *F*(*n*) contains $M!/(n!\left( {M - n} \right)!)$ elements it can be very large, e.g. $\left| {F\left( {12} \right)} \right|\sim 2.7 \cdot 10^6$ for *M* = 24. We thus used the discreteness of the occurring fractional values, i.e. the fact that the 24 fractions take at most $1 \le K \le 6$ different values. The probability for *n* successes can then be rewritten as $pr\left( n \right) = \mathop {\sum}\nolimits_{i = 1, \ldots ,L} {\mathop {\prod}\nolimits_{k = 1 \ldots K} {b\left( {N\left[ k \right],\,n[i,k]} \right)p_k^{n\left[ {i,k} \right]}\left( {1 - p_k} \right)^{N\left[ k \right] - n\left[ {i,k} \right]}} }$, in which the ith row $n\left[ {i, \cdot } \right]$ of the *L* × *K* partition matrix $n\left[ { \cdot , \cdot } \right]$ denotes the ith partition of *n* into *K* integers not exceeding the respective class numbers *N*[*k*] (gtools R-package, version 3.5.0).

### Tumour growth model

Spherical tumours of diameter 0.6 and 10 mm may contain $N_{0.6} = 2.2\cdot 10^4$ and *N*_10_ = 10^8^ tumour cells, respectively, assuming a melanoma cell diameter of 18 μm^48^ and a tumour cell fraction of 60%^49^. Interestingly, this corresponds to the volume fractions of 52% for simple cubic and of 74% for hexagonal close spherical packing. In an exponential growth model, tumours of diameter 0.6 and 10 mm result from ${\rm{log}}_2(N_{0.6}) = 14.4$ and ${\rm{log}}_2(N_{10}) = 26.6$ cell divisions as indicated in Fig. 6a, d, g, j.

### DCCD thickness model

Principle: We assume exponential growth kinetics for both the PT and the SLN colony starting from a single-cell, i.e., the number of PT cells is given by $M_{\mathrm{T}} = 2^{g_{\mathrm{T}}t}$ and in the SLN cells obey $M_{\mathrm{L}} = 2^{g_{\mathrm{L}}(t - t_{\mathrm{L}})}$, with $t_{\mathrm{L}} \le t$ the time of dissemination to the SLN (start of growth). The base-2 logarithm of *M*_T_ and *M*_L_ indicates the respective number of cell divisions, i.e., $N_{\mathrm{T}} = g_{\mathrm{T}}t$ for the PT and $n_{\mathrm{L}} = g_{\mathrm{L}}\left( {t - t_{\mathrm{L}}} \right)$ for the SLN. The number of cell divisions within the PT incurred by cells eventually disseminating to the SLN is given by $n_{\mathrm{T}} = g_{\mathrm{T}}t_{\mathrm{L}}$. We propose a model, in which cell divisions within the PT or within the SLN can have a differential influence on the occurrence of genomic alterations, a notion that is also supported by recent literature^50^. Accordingly, we define a corresponding risk function

$$E = an_{\mathrm{T}} + bn_{\mathrm{L}} = aN_{\mathrm{T}} + \left( {b - a\hat g} \right)n_{\mathrm{L}}$$

with $\hat g = g_{\mathrm{T}}/g_{\mathrm{L}}$ the ratio of growth rates and $n_{\mathrm{T}} = N_{\mathrm{T}} - \hat gn_{\mathrm{L}}$. Based on our experimental observations, we consider four major categories of genomic alterations. First, alterations whose risk of occurrence exclusively depends on the number of cell divisions within the PT before dissemination. Accordingly, setting *a* = 1 and *b* = 0 leads to $E = N_{\mathrm{T}} - \hat gn_{\mathrm{L}}$ implying that curves of equal risk $\left( {E = E_0 = {\rm{const}}} \right)$ are ascending lines $y = E_0 + \hat gx$ in the *x*–*y* plane $\left( {x = n_{\mathrm{L}},y = N_{\mathrm{T}}} \right)$. Second, we regard alterations for which the total number of cell divisions is most important, i.e., irrespective of the environment. Here, *a* = *b* = 1 results in $E = n_{\mathrm{T}} + n_{\mathrm{L}}$. For equal growth rates (i.e., $\hat g = 1$), this scenario corresponds to risk proportional to the number of PT cell divisions (i.e., *E* = *N*_T_) and thus horizontal equal risk lines. Third, we include alterations for which cell divisions within the SLN are considerably more important than in the PT. Accordingly, for *a* = 1 and $b = 2\hat g$ the risk becomes $E = N_{\mathrm{T}} + \hat gn_{\mathrm{L}}$ resulting in descending equal risk lines $y = E_0 - \hat gx$. Fourth, we account for alterations for which the number of cell divisions within the SLN are most decisive. Here, choice of *a* = 0 and *b* = 1 results in *E* = *n*_L_ and thus vertical equal risk lines. Referring to our previous work^13^ alterations falling into the third category appear most important for patient survival. Notably, we do not presently differentiate between risk of gain and loss (die out) of alterations.

Linear classifiers: Linear classification was performed in scaled log(DCCD)-log(thickness) space, i.e., log values were scaled to the unit interval independently for each variable. Each classifier consisted of a 2D set point $p = (\rm{scl.DCCD,scl.thick})$ of scaled logarithmic DCCD and thickness values and an angle $\varphi \in [0,180)$ specifying the direction of the classifier’s normal vector. 2D set points were chosen according to the experimental DCCD and thickness values but displaced by ±*δ*/2 with *δ* the minimal absolute difference between the experimental DCCD and thickness values, respectively. This resulted in 4 displacement set points per DCCD thickness pair and enabled unequivocal classification of each sample. Angles were scanned as per 1° step size. Classification was performed by constructing difference vectors between all experimental values and the set point and evaluating the sign of the corresponding scalar products regarding the normal vector. Classification results were assessed by applying Fisher’s exact test to the corresponding 2 × 2 or 2 × 3 contingency tables and FDR-adjusted *p* values were calculated across 82 loci/mut. In Fig. 6b, e, h, k, only the lowest FDR value across displacement set points and angles is displayed for each DCCD-thickness pair. The mapping between log(DCCD) and the number of cell divisions in the SLN is basically qualitative. To arrive at maximum classifier angles of about 135° as predicted by theory in case of equal growth rates $\left( {\hat g = 1} \right)$, we derived corrected angles according to re-scaling the log(DCCD)-axis by a factor of 1/2. Finally, large angles near 180° were transformed to small angles near 0° by subtracting 180° (Fig. 7a).

Sample weights: Multiple DCCs from a single individual may tend to be more similar to each other than do DCCs derived from different patients. This can be a confounding factor when analysing patient collectives, in which the number of DCCs per patient varies. A commonly applied corrective are patient weights, i.e. coefficients *w*_*i*_ = 1/*n*_*i*_ that down-weight samples according to the number (*n*_*i*_) of samples of patient *p*_i_. The effective number of samples available for statistical analysis is then calculated as $M = \mathop {\sum}\nolimits_{i = 1 \ldots P} {w_in_i}$, here equalling the number of patients *P*. Generally, *P* is smaller than the sum of measured samples $N = \mathop {\sum}\nolimits_{i = 1 \ldots P} {n_i}$ implying that statistical power is lost in this approach if multiple patient samples are at least partially representative of the whole population. We thus aimed at increasing sample weights within an admissible range defined by high equivalence of the corresponding distributions of genomic features. Specifically, we compared the distribution of amplifications (+1), balances (0) and deletions (−1) for genomic loci *l* between the patient weight and our admissible weight model. In our model, weights are maximised within the range $1/n_i \le w_{l,i} \le 1$, while the corresponding *p* value of statistical testing is kept above a limiting value (*p* = 0.95). For spatial sample classification (here, regarding regions within the DCCD-thickness plane) it is important that the distributional equivalence holds both globally and locally. As a consequence, we conducted global tests enclosing all samples, as well as local tests defined by *k* nearest patient neighbours (*k* = 5). Numeric computation in R was time intensive and thus parallelised using the R-packages foreach (version 1.4.3) and doParallel (version 1.0.10). The use of genomic locus-specific weights can be justified by the fact that patient DCC clonality can be locus-specific (Hoffmann, Klein, et al., unpublished results). We note that the power approach to equivalence testing, which requires sufficient (80%) power for detecting some reasonable difference *δ* in addition to a high *p* value of difference testing has been invalidated^51,52^. Different equivalence testing methods have been proposed in the literature, the most prominent being the family of two one-sided-tests (TOST)^52,53^. Nevertheless, there appears to be only one method^54^ addressing contingency tables, which, moreover, is restricted to 2 × 2 tables and the *χ*^2^ test. Here, we dealt with 2 × 3 tables and applied Fisher’s exact test because of low case numbers. We could thus not employ present equivalence testing procedures. While these tests are appealing from a theoretical point of view, they still have their own unsolved problems regarding e.g. the choice of an appropriate margin *δ* (specifically for contingency tables) or issues concerning the rejection region^52^. Nevertheless, we expect sample weights resulting from difference testing as applied in this study to be largely similar to corresponding results of future expanded equivalence testing methods. Sample weights apply to the results shown in Figs. 5b, 6, 7a, 7b.

Fisher’s exact test for non-integer tables: *χ*^2^ testing is advised against if contingency table entries are small (<5), which applies to our data since deletions were often rare. Moreover, we used testing as a means to evaluate classification results, for which some zero case numbers are actually desired, e.g. for the off-diagonal elements of 2 × 2 contingency tables. Fisher’s exact test is unrestricted regarding case numbers but can only be applied to integer tables. To make Fisher’s method available for non-integer analysis (sample weights), we chose to interpolate the *p* values of neighbouring integer contingency tables as follows. Let *r* be the number of non-integer entries of a contingency table that are summarised in an *r*-dimensional vector *v*_*r*_. We construct an *r*-dimensional simplex *s*_*r*_ that is rooted at the integer-rounded vector [*v*_*r*_] and is directed towards *v*_*r*_. Subsequently, the Fisher test *p* values corresponding to the *r* + 1 integer vertices of the simplex are calculated and used to linearly approximate the *p* value of the contingency table.

### Mutation analysis of *BRAF* and *NRAS*

Mutations in *NRAS* and *BRAF* genes were detected using Sanger sequencing (Sequiserve, Vaterstetten, Germany) after gene-specific amplification from WGA samples. The primers for *BRAF* exon 15 analysis were as follows: forward 5′-TCCAGACAACTGTTCAAACTG-3′ and reverse 5′-CTCTTCATAATGCTTGCTCTG-3′, encompassing the mutations of codon 600 (V600E, previously called V599E; V600K, V600R). Cycling temperatures were set to 94 °C (2 min), 60 °C (30 s) and 72 °C (2 min) for one cycle; 94 °C (15 s), 60 °C (30 s) and 72 °C (20 s) for 14 cycles; 94 °C (15 s), 60 °C (30 s) and 72 °C (30 s) for 24 cycles and an additional final extension step at 72 °C (2 min). The PCR primers for *NRAS* exon 3 codon 61 analysis were: forward 5′-GGCAAATACACAGAGGAAGC-3′ and reverse 5′-ACCCCCAGGATTCTTACAGA-3′ encompassing the common mutations of codon 61: Q61K and Q61R. The PCR cycler was set to 94 °C (2 min), 63 °C (30 s) and 72 °C (2 min) for one cycle; 94 °C (15 s), 63 °C (30 s) and 72 °C (20 s) for 14 cycles; 94 °C (15 s), 63 °C (30 s) and 72 °C (30 s) for 24 cycles and an additional final extension step at 72 °C (2 min). PCR products were sent for sequencing to Sequiserve (Vatterstetten).

The mutation assay was established using single cells or genomic DNA of cell lines with known exon 15 mutation c1799T>A (*BRAF*) and exon 3 mutation c181C>A (*NRAS*). The mutant *BRAF* allele was detected in 62% (70–61), 84% (MelHo) of detected sequences over all analysed single cells and in 61% (70–61) and 86% (MelHo) in bulk genomic DNA. The mutation *NRAS* allele was present in 59% of all single cells and 46% of the bulk genomic DNA.

When several areas of the PT were microdissected or several DCCs were isolated, the PT or DCCs were called positive if one of the areas or DCCs harboured the *BRAF* or *NRAS* mutation.

### Assessment of the allelic drop-out rate

To determine the allelic drop-out rate (ADO) for BRAF/NRAS mutational analysis of single cells, 10.000 melanoma cell line cells of known BRAF/NRAS mutational status were added to lymph nodes of healthy controls and mechanically disaggregated and stained for immunocytology as described in the section 'Lymph node disaggregation and immunocytology'. Single cells were isolated, WGA performed and the allelic frequency of the BRAF and NRAS mutations in individual cells was determined as described in the corresponding sections above. To determine the ADO in single cells from human primary tumours or DCC-derived xenografts of patients with known mutational BRAF/NRAS status, tumour tissue was digested with collagenase (0.33 µg/ml, Sigma-Aldrich) and DNAseI (0.1 mg/ml Roche) to obtain a single-cell suspension. After gp100-staining, individual cells and cell pools were manually isolated, WGA performed and the allelic frequency of the BRAF and NRAS mutations was determined as described in the corresponding sections above.

### Statistical analysis

Unless otherwise stated, statistical significance was assumed for *p* < 0.05, with all tests performed two-sided.

Statistical methods for the analysis of dissemination and colonisation: To determine the proportion of disseminating and colonising tumours as a function of tumour thickness, the data were fitted non-parametrically by maximum-likelihood according to the iterative method of Turnbull^55^ for interval-censored data for the following reason: for a patient with a certain tumour thickness, it can be observed only whether dissemination or colonisation has occurred but the exact tumour thickness at the time when the event occurred, or would occur, remains unknown. If the event has occurred, the tumour thickness at the time of the event was at least zero and at most equal to the tumour thickness at the time of observation. If the event has not yet occurred, the lower limit of the tumour thickness is known. This situation is identical to survival analysis when all the observations are interval censored, for which a nonparametric maximum-likelihood-estimator is suggested as by Turnbull^55^. Both nonparametric distributions are summarised by parametric models that allow a better interpretation of the data. Therefore, we fitted the following five distributions: (1) standard log-logistic, (2) exponential, (3) log-normal, (4) Weibull and (5) Fréchet. For the dissemination data, we introduced in addition a parameter, which estimates the maximum proportion of patients who disseminate. The parameters of the distributions were fitted by maximum-likelihood, taking into account interval censoring. We also estimate the 95% confidence intervals of the distributions, from which we derive the 95% confidence intervals of certain quantiles. The selection of the best model is based on the Bayes information criterion (BIC), which takes into account the likelihood function, the number of parameters and the number of observations. The goodness of fit of the models was assessed by grouping the 1027 values of tumour thickness for dissemination into deciles and the 525 values of tumour thickness for colonisation into quintiles. The observed number of patients with a positive event was compared with the fitted number of events by a *χ*^2^ statistic. The goodness-of-fit test takes into account the number of categories (ten for dissemination, five for colonisation) and the number of estimated parameters. All five models yield a good fit to the data, but we adopt the simplest models with the minimum number of parameters.

The hazard rate describes the instantaneous risk for an event (dissemination and colonisation) for those tumours, for which the event has not yet occurred. It can be calculated explicitly by the density of the fitted distribution divided by the exceedance probability, which is obtained by subtracting the cumulative distribution function from 1.

Comparison of PTs and DCCs: The frequency statistics of gains and losses between PTs and DCCs were determined with a Mann–Whitney *U* test. Statistical significance for *BRAF*/*NRAS* mutations in paired PTs and DCCs was determined with Fisher’s exact test.

Identification of mutational patterns: Aberration/mutation patterns that discriminate between PTs and DCCs, DCCs from patients with different DCCD and thickness values, as well as with and without *BRAF*/*NRAS* mutations were identified by Fisher’s exact test. Only loci with high enough cross-sample standard deviation (>0.25) allowing for sufficient class discrimination were considered. In Fig. 2b, only the ten most variable loci in terms of the maximum variance across samples without accounting for PT/DCC class labels were included. Multiple testing corrections (across loci/mutations) were derived according to Benjamini and Hochberg (FDR).

Identification of discriminating mutations: To identify the thickness at which critical alterations may have been acquired within the PT, we split DCCs of pre-colonisation samples (defined by a DCCD ≤ 105, see below) into two groups according to the measured thickness values and identified genomic alterations that clearly showed non-random distributions across these groups, i.e., lead to low Fisher’s exact test *p* values. DCCD and thickness thresholds associated with low *p* values might indicate PT sizes and genomic alterations that facilitate direct dissemination to SLNs. We tested all DCCD thresholds up to 105 to define the population of DCCs before colonisation and found evidence for statistical differences for certain thickness thresholds and DCCD limits (Fig. 5b); however, none reached 5% significance.

An equivalent approach was taken to determine limiting DCCD thresholds and genetic alterations that mark the transition from early DCCs to colony-forming DCCs in the SLN. We asked for each genetic region at which DCCD it would split all DCCs into two groups that significantly differ in their distribution of gains and losses. This DCCD would then separate DCCs with and without that specific alteration and indicate the DCCD at which an alteration critical for disease progression has been acquired (Fig. 7b).

Hierarchical cluster analyses (Fig. 2b and Supplementary Figure 5) were performed using Euclidean distance and complete linkage. Analyses were conducted using R (version 3.3.1, http://www.R-project.org) or JMP (http://www.jmp.com).

Survival analysis: All survival statistics and tumour-free time of xenografts were calculated using a log-rank test (JMP, IBM SPSS Statistics 20 for Windows or GraphPad Prism 6.0 software for OSX).

### Data availability

The data sets generated during and/or analysed during the current study are available from the corresponding author on reasonable request.

## Electronic supplementary material

Supplementary Information
