# Supplementary material for: Genetic alterations driving metastatic colony formation are acquired outside of the primary tumour in melanoma
Source: Nat Commun. 2018 Feb 9;9:595. doi: 10.1038/s41467-017-02674-y (PMC5807512; doi:10.1038/s41467-017-02674-y)
Supplement: Supplementary file 1 — Supplementary Information [file 41467_2017_2674_MOESM1_ESM.pdf]

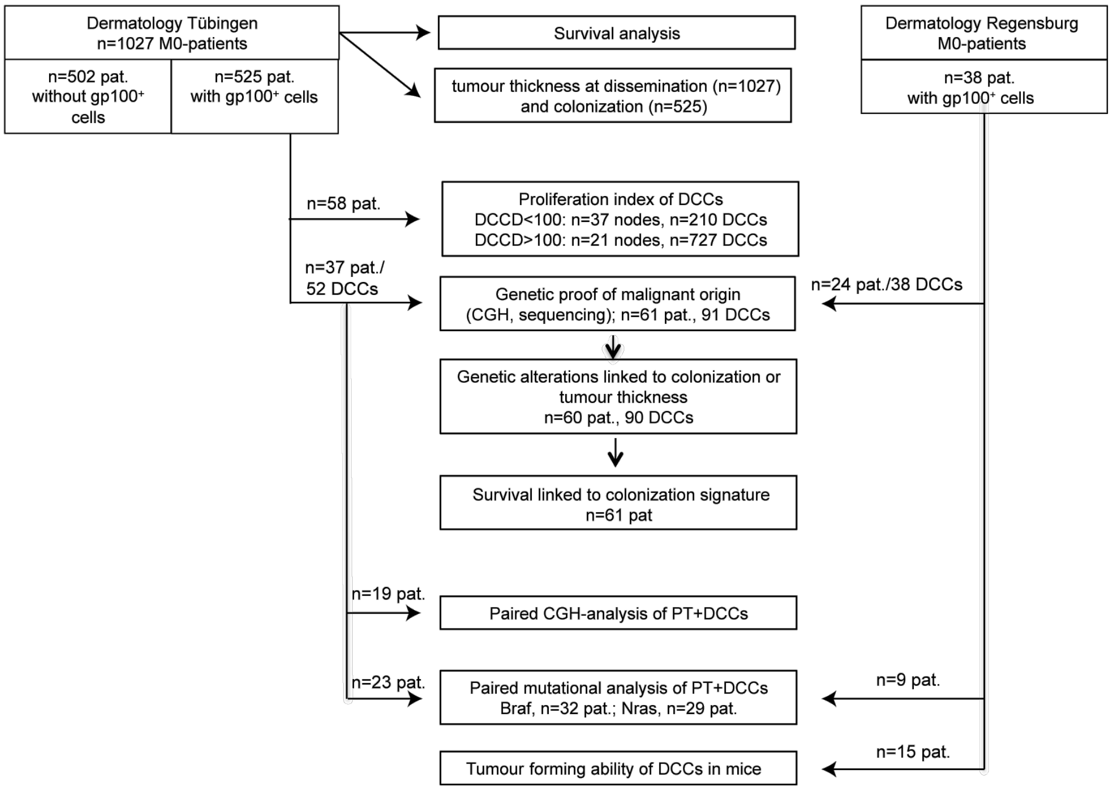

Supplementary Figure 1. Summary of analyzed patient samples. Our study focuses on melanoma patients in their earliest disease stages; therefore only non-metastasized (M0) patients, free of distant metastasis and with clinically node-negative disease were included (see also Supplementary Figure 2). For molecular analysis, samples were included according to availability or DNA-quality. Our criteria for selection of patients/cells for further genomic or functional analysis included:

1) Patients had $gp100^+$ cells in their SLN.
2) Gp100+ DCCs were isolated.
3) DCC-derived DNA passed quality control for CGH analysis (see ref [1]) and high-quality sequences for BRAF/NRAS mutations could be obtained.
4) Paraffin-blocks of primary tumours (PTs) could be received from external dermatologists. (Note, that acquisition of primary tumours is extremely difficult as almost all patients are operated outside of the University hospital).
5) Sufficient material of PTs was left and not completely used for diagnosis.
6) DNA from many paraffin-embedded tissues passed the quality control.
7) Sufficient SLN material to allow short-term culture or xenotransplantations.

Follow-up was available and of sufficient length.

Control Cells

2316 KMN1

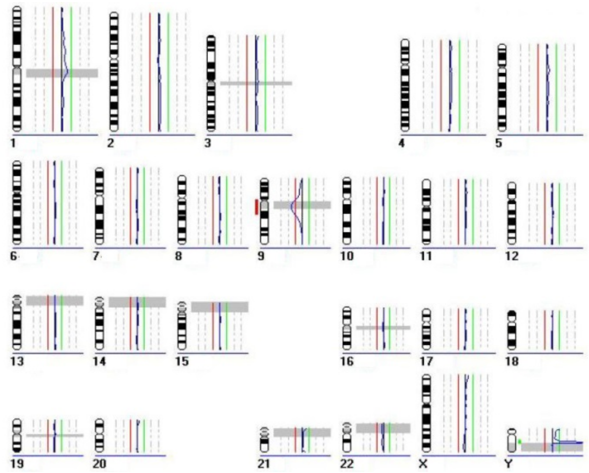

2337 KMN1

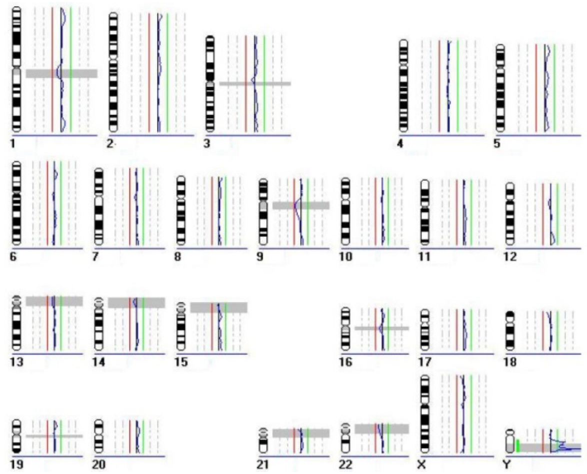

2349 KMN1

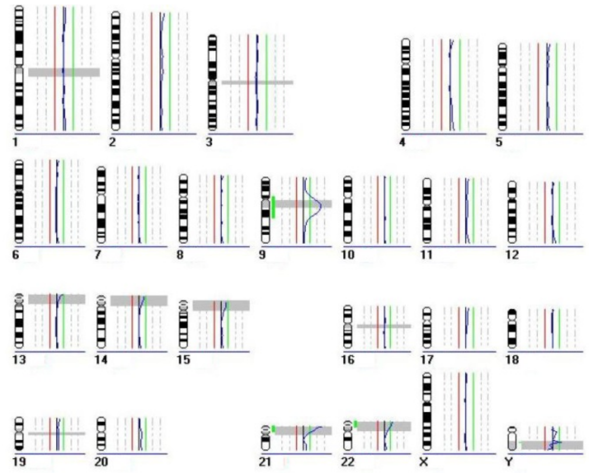

2349 KMN2

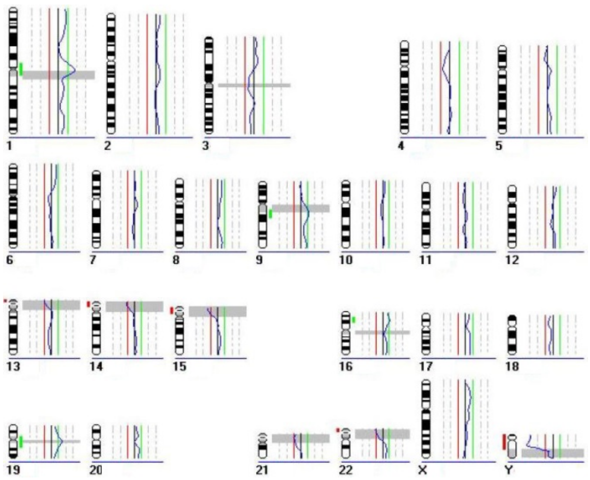

2436 KMN1

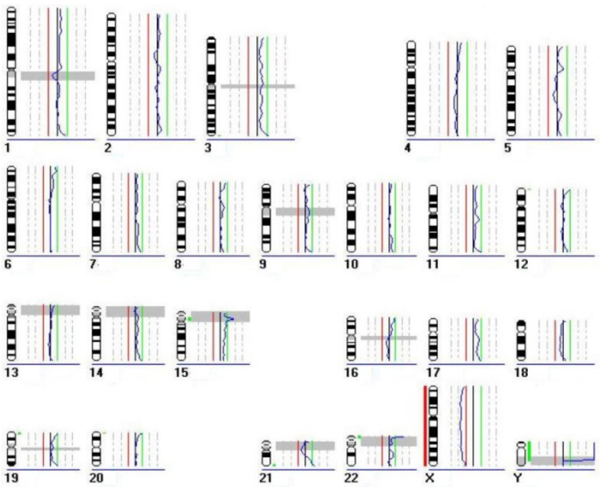

2436 KMN2

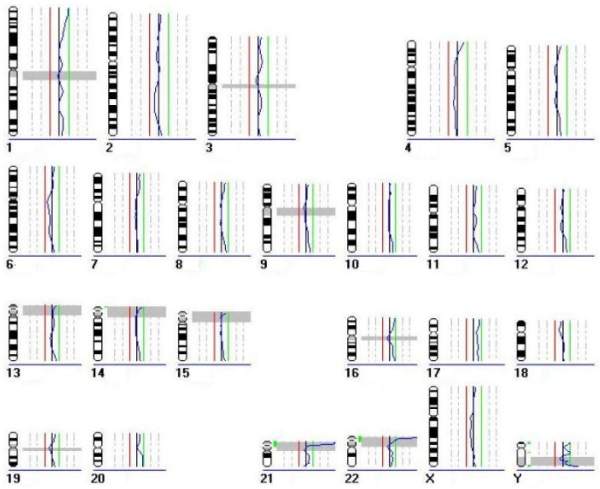

2436 LKN1

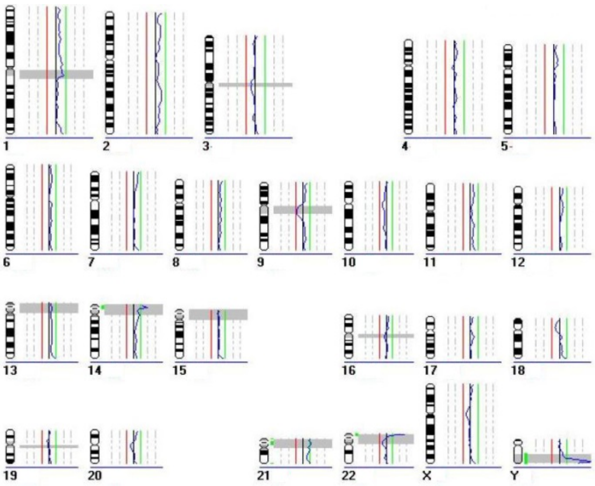

2501 KMN1

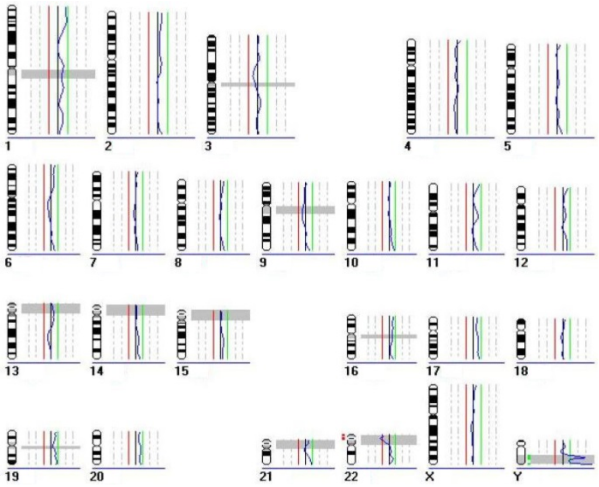

3214 LKN1

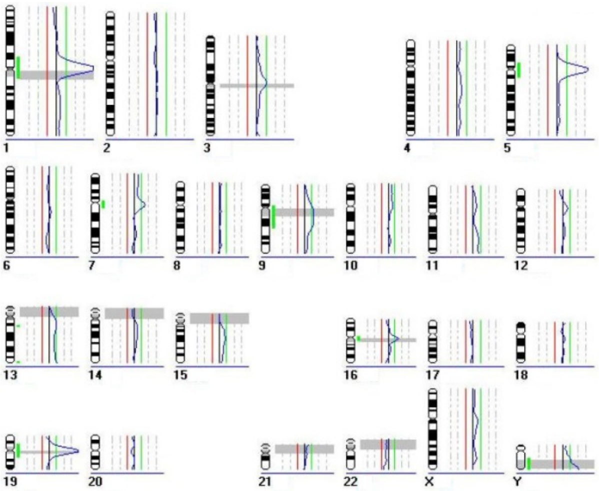

3425 KMN1

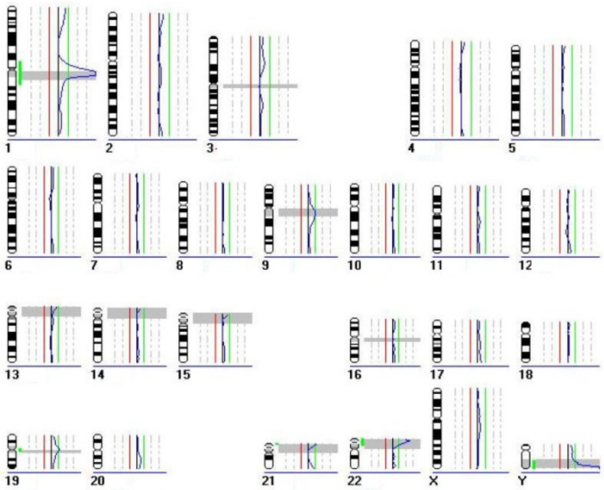

3596 KMN1

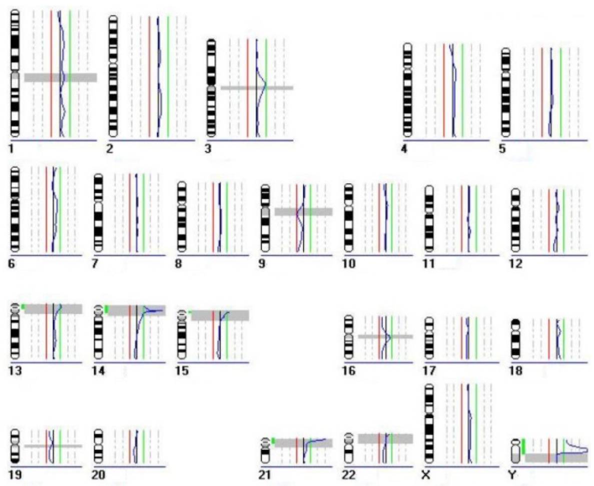

3641 KMN2

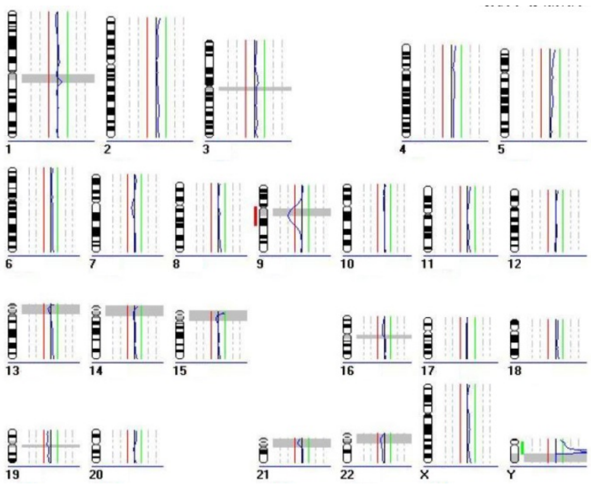

778 NZ1

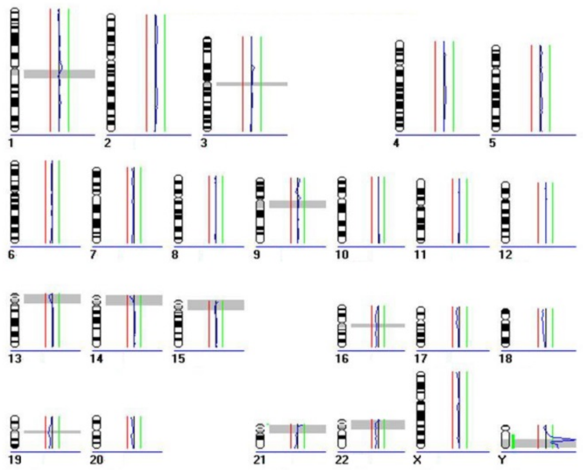

778 NZ2

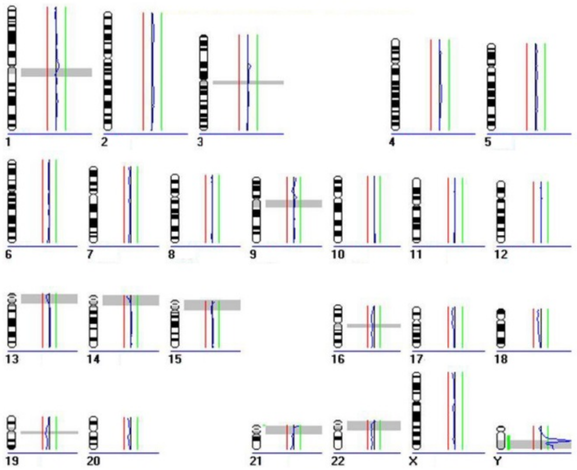

1032 NZ1

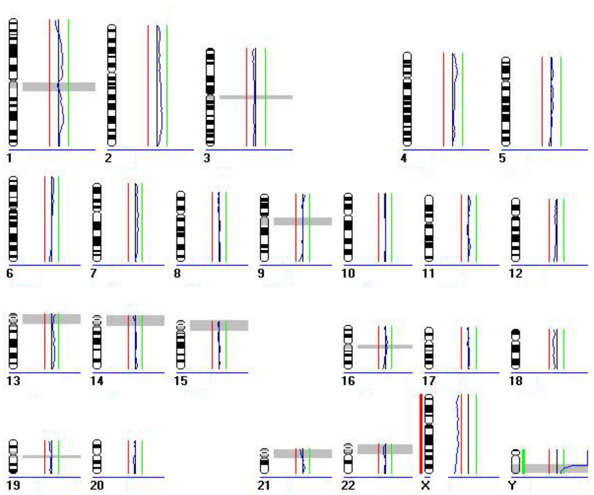

1032 NZ2

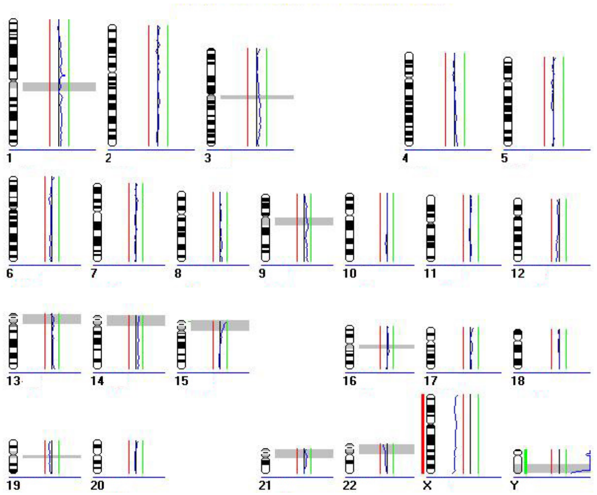

1077 NZ2

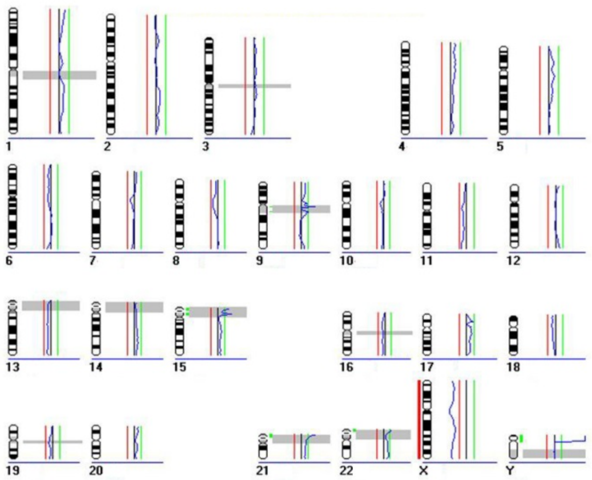

LK 16 N1

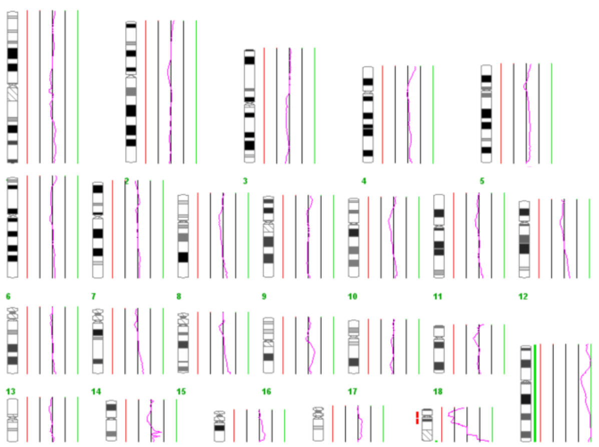

LK 16 N2

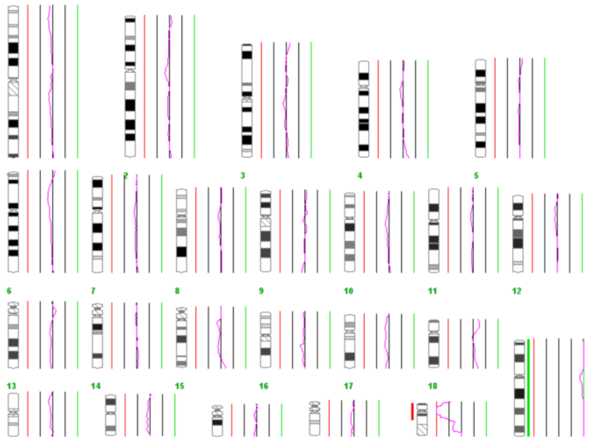

LK 16 N3

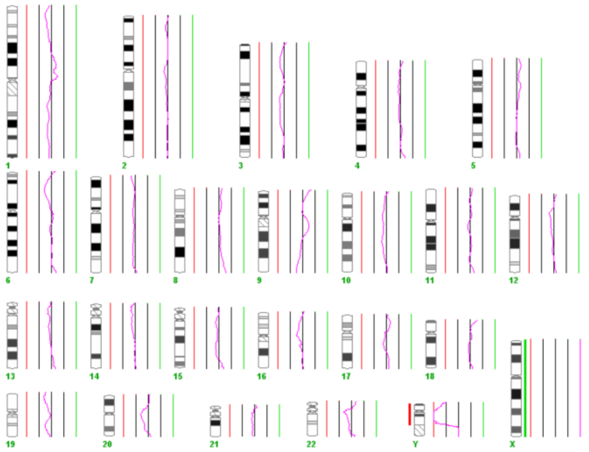

RPC 94 N

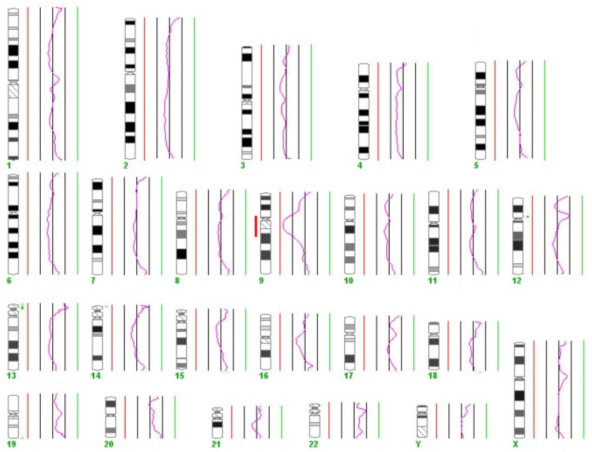

114 N

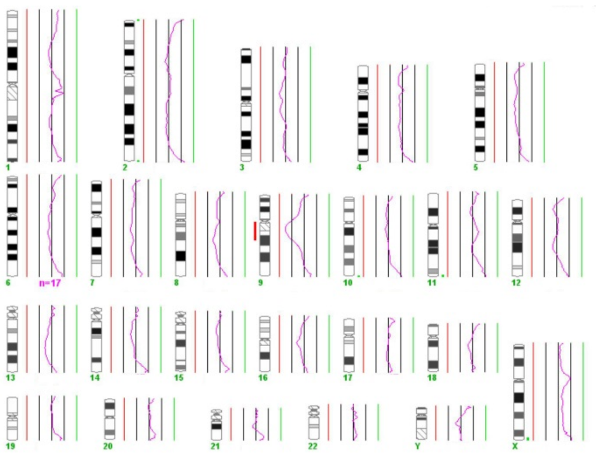

856-3b

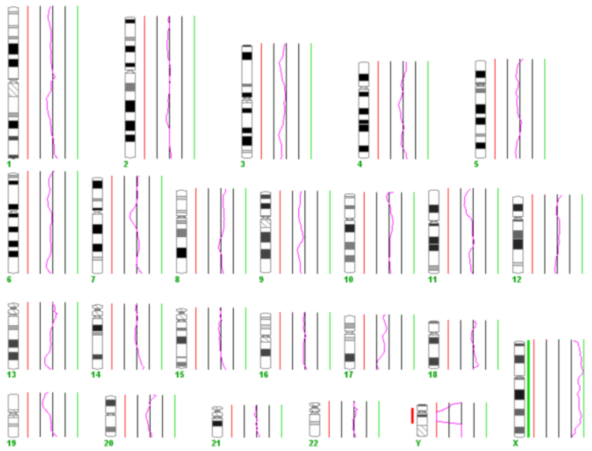

741-9SMb

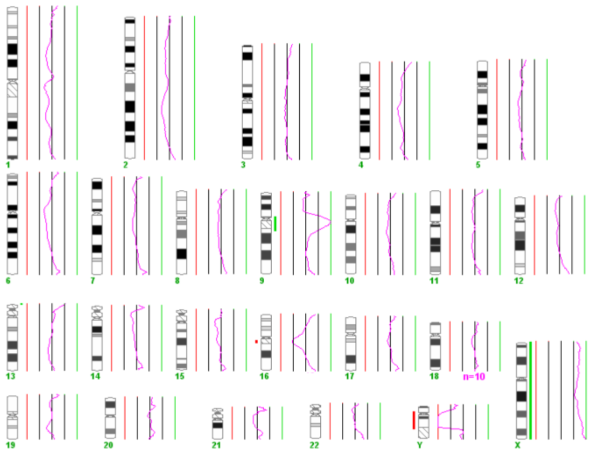

983-9SMb16

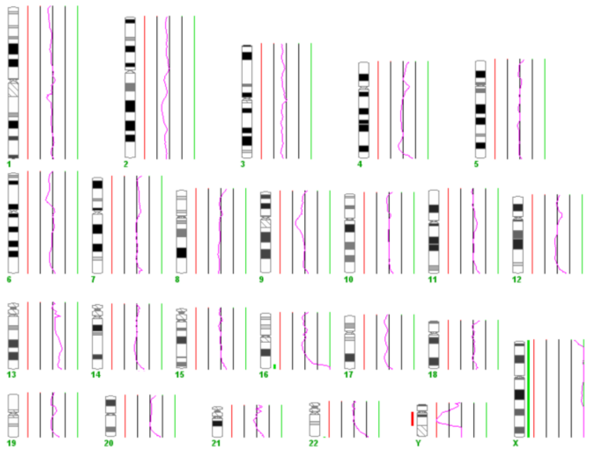

PBL SC 1

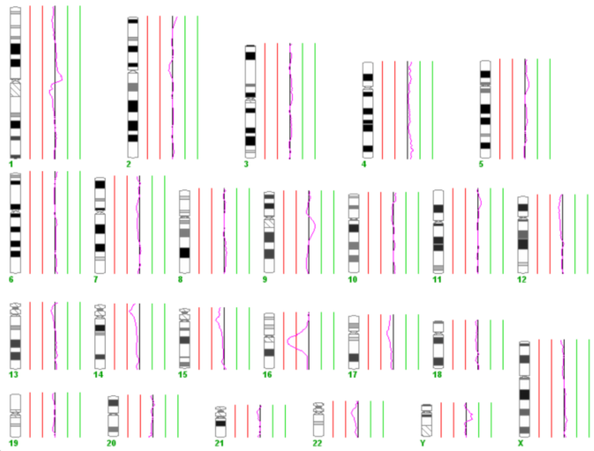

PBL SC 2

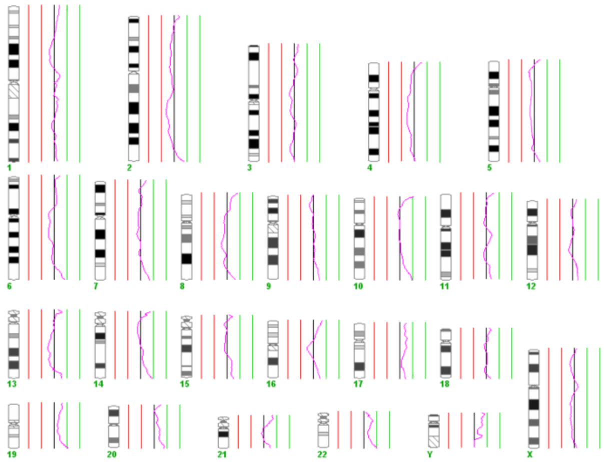

PBL SC 3

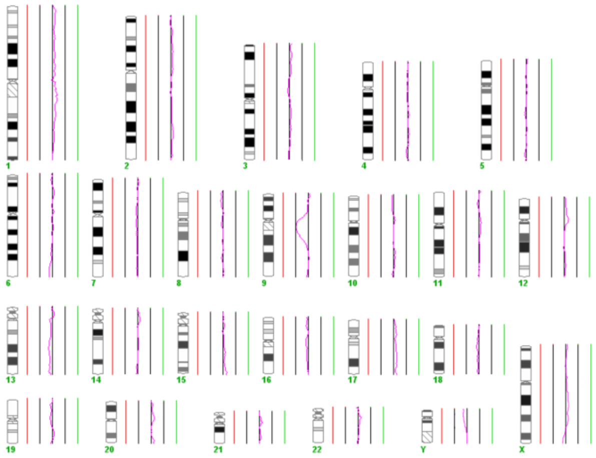

PBL SC 4

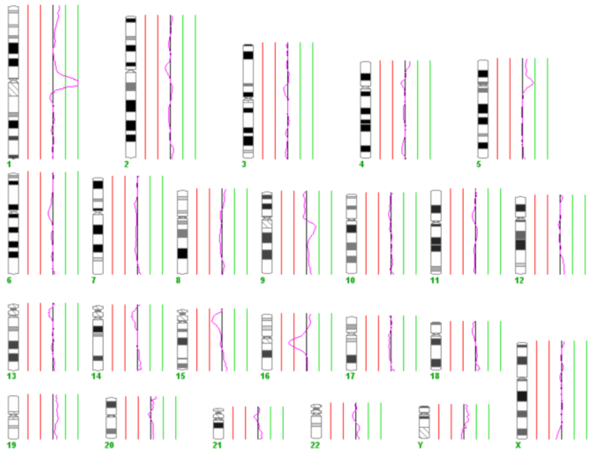

PBL SC 5

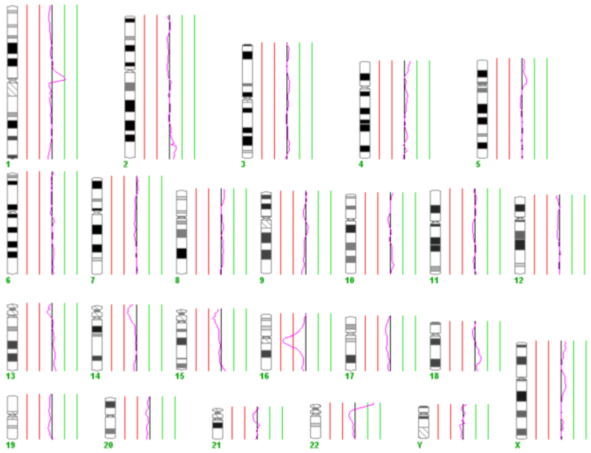

Supplementary Figure 2. CGH profiles of control cells. CGH profiles of 30 control cells isolated from blood, lymph node or bone marrow. Cells were identically isolated and amplified as gp100-positive cells. All chromosomes are depicted. In some cases, we used sex-mismatch control DNA to demonstrate successful hybridization. Coloured bars indicate relative gains or losses of the X-chromosome (red, underrepresentation in test cells; green, overrepresentation in test cells) bars next to the ideogram. Chromosomal regions (centromeric regions) marked by gray bars are excluded from analysis as they contain repetitive regions. All cells displayed normal genomes.

CGH-Profiles of patients with PT/DCC pairs

Primary tumour

T01 PT1

chromosome 9:example for heterochromatin that was **not** considered;
Sex mismatch hybridization: X/Y excluded;

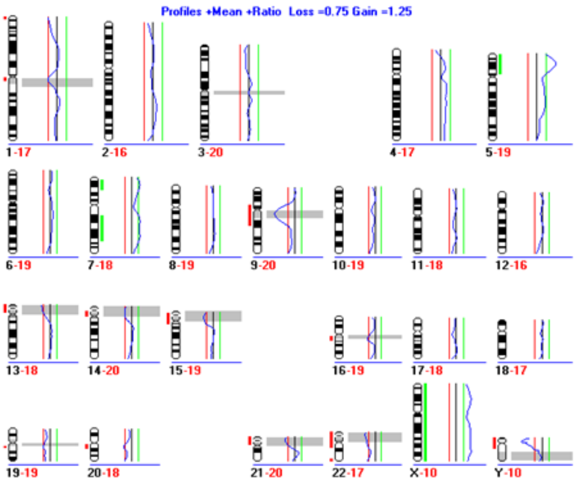

T02 PT1

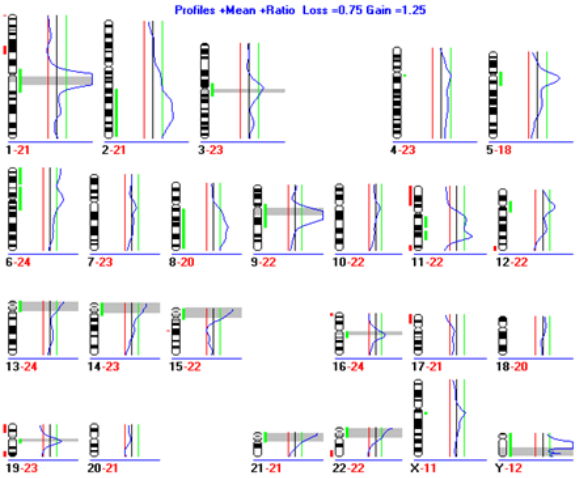

DCCs

T01 DCC1:

chromosome 1:example of telemetric gain that was considered;
chromosome 3: example of telemetric gain that was **not** considered;

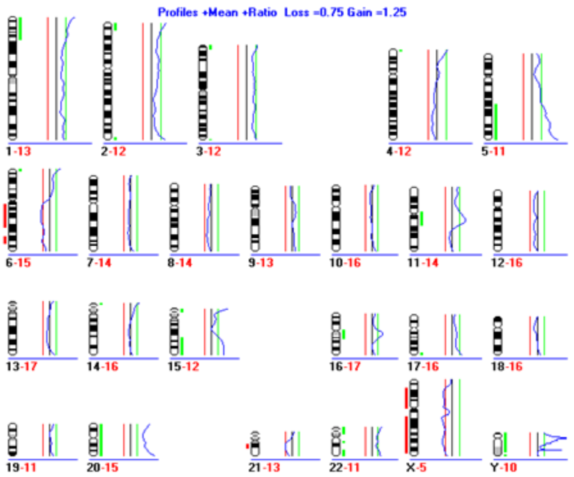

T02 DCC1

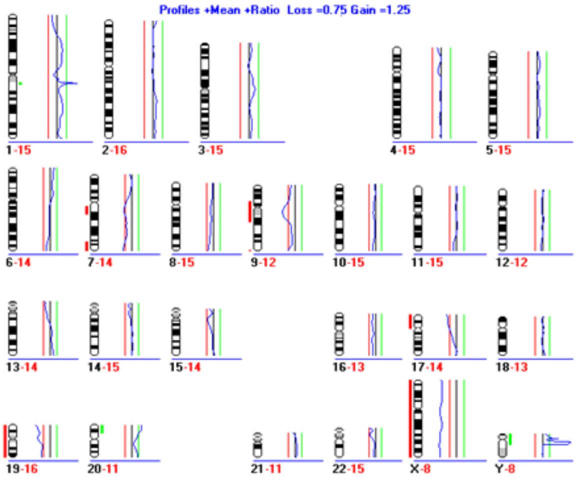

Primary tumour | DCCs

T03 PT1

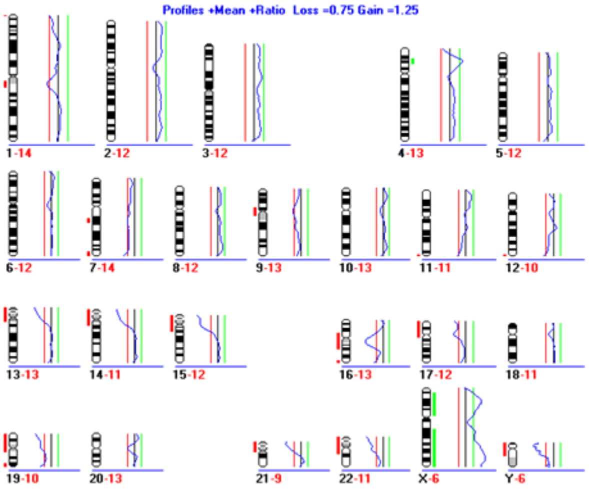

T03 DCC5

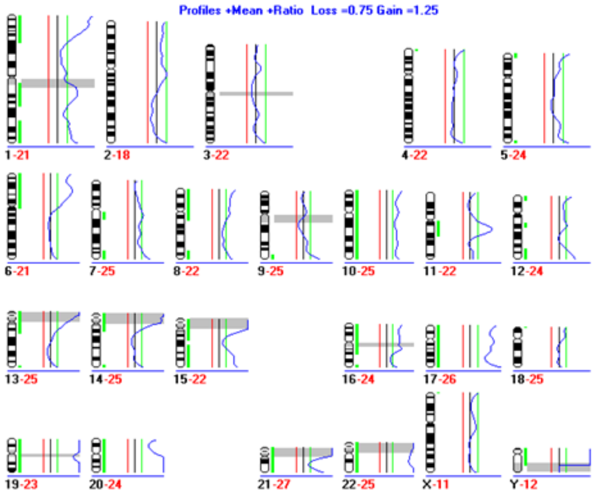

T05 PT1

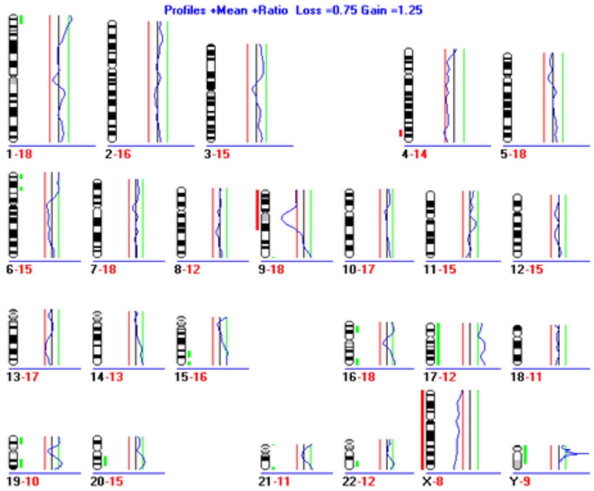

T05 DCC1

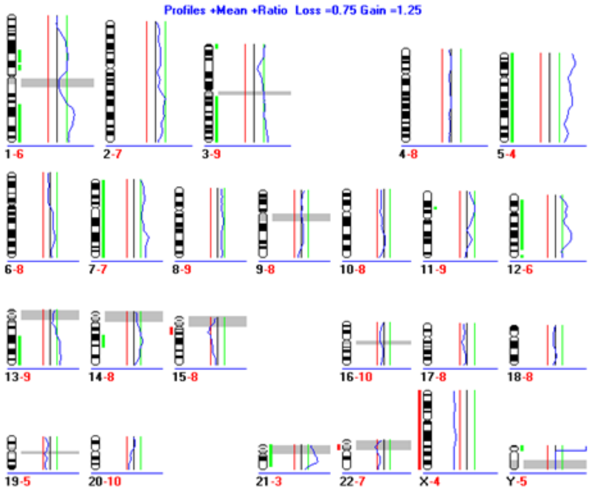

T06 PT1

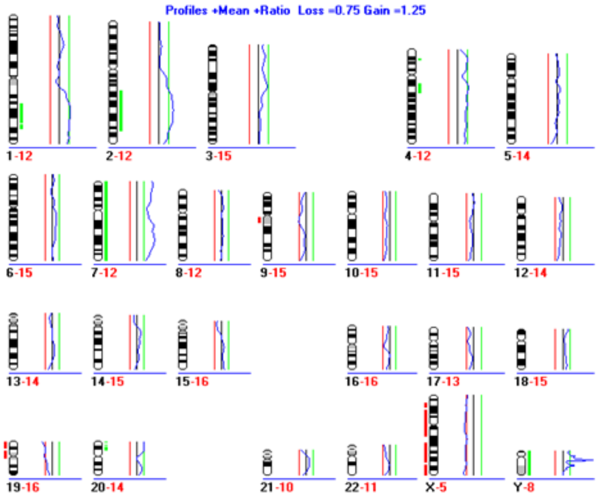

T06 DCC1

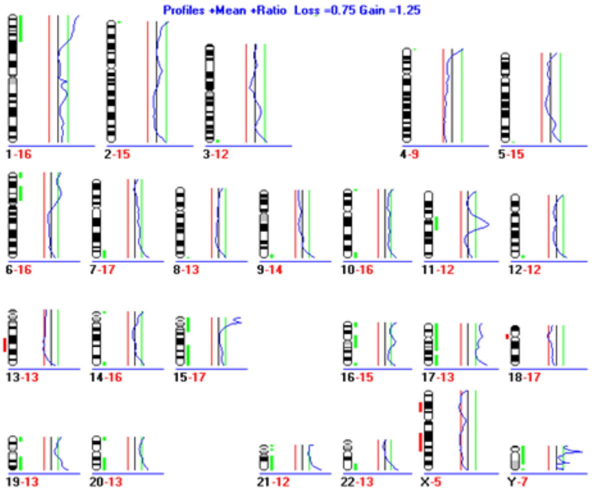

Primary tumour

DCCs

T07 PT1

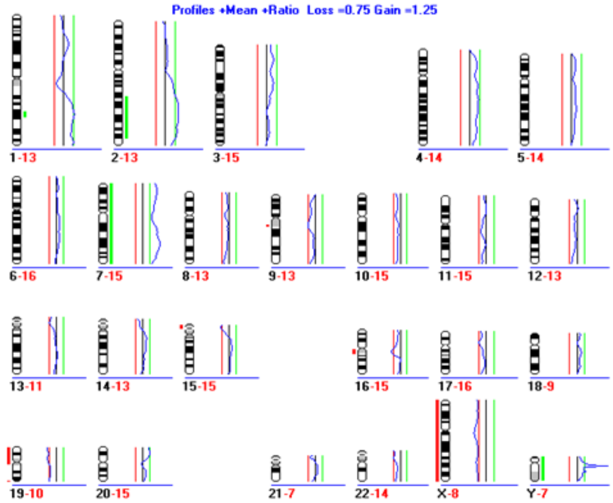

T07 DCC2

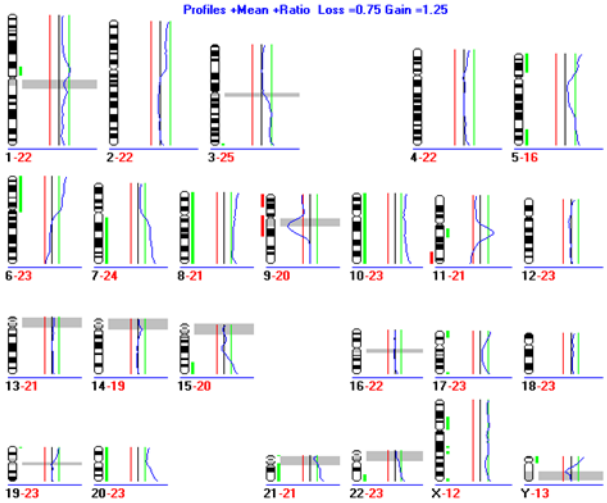

T08 PT1

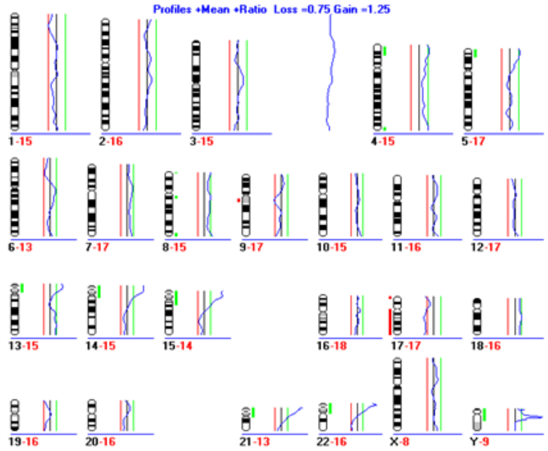

T08 DCC1

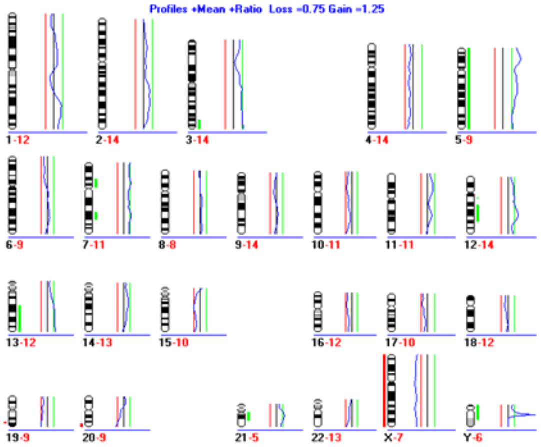

T08 DCC3

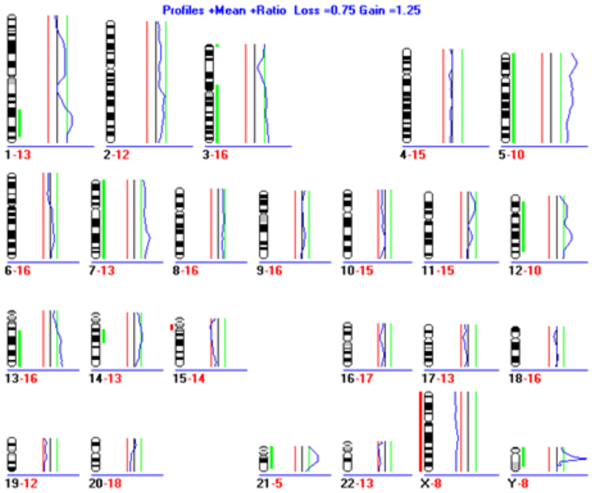

Primary tumour

DCCs

T09 PT2

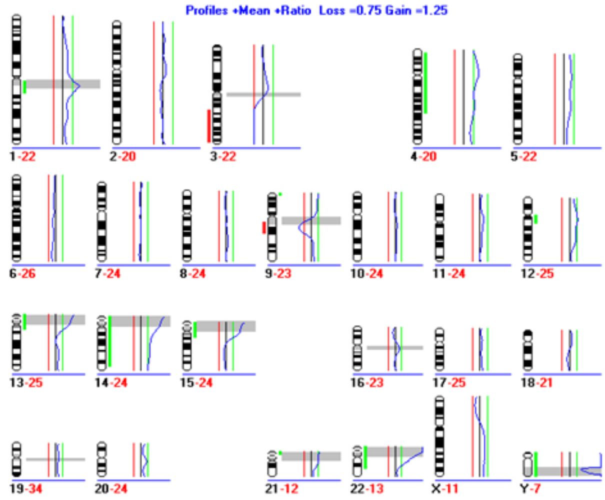

T09 DCC1

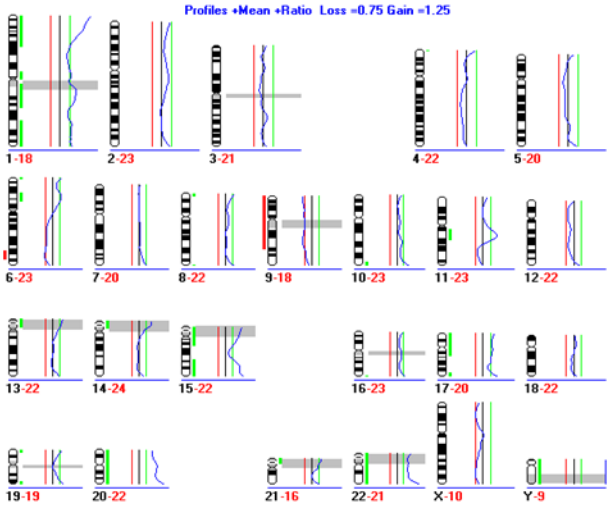

T09 PT3

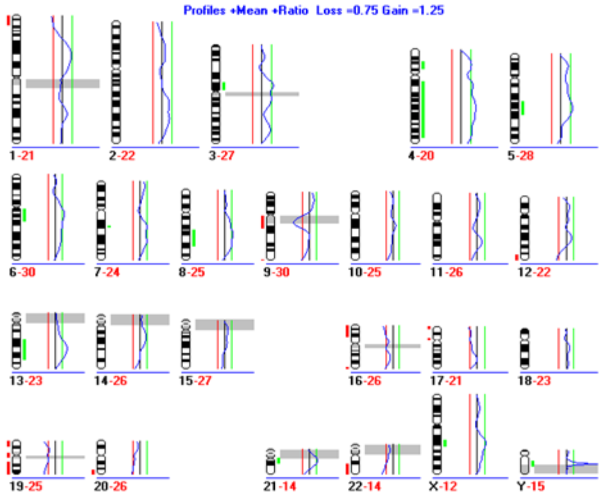

T09 DCC2

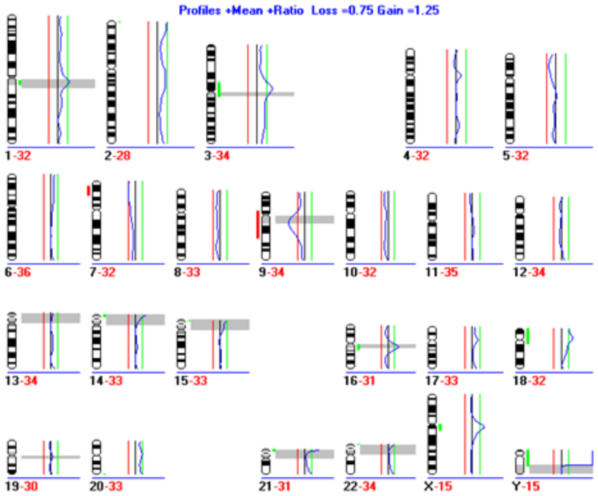

T09 DCC3

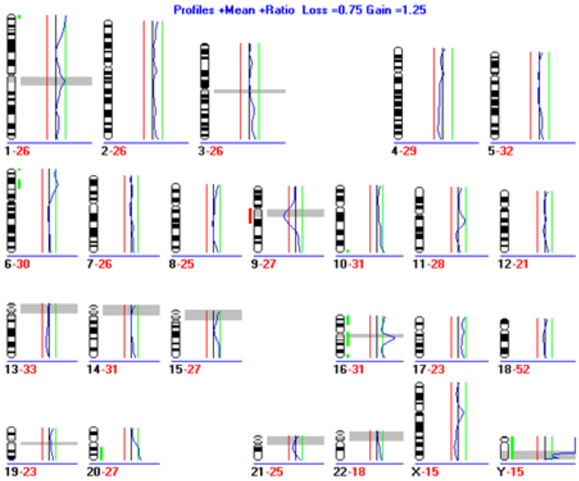

Primary tumour

DCCs

T10 PT1

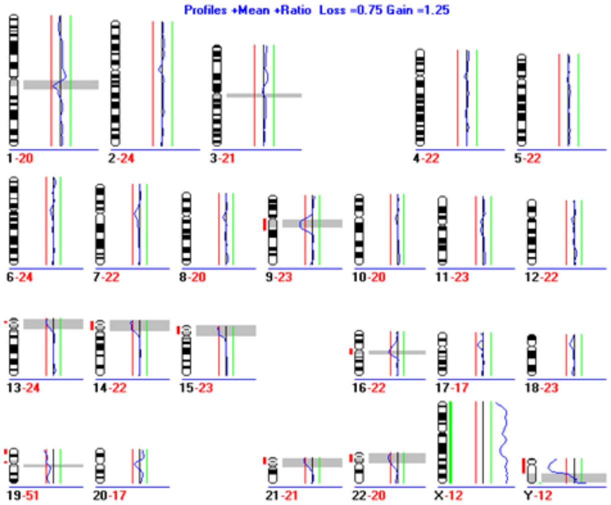

T10 DCC1

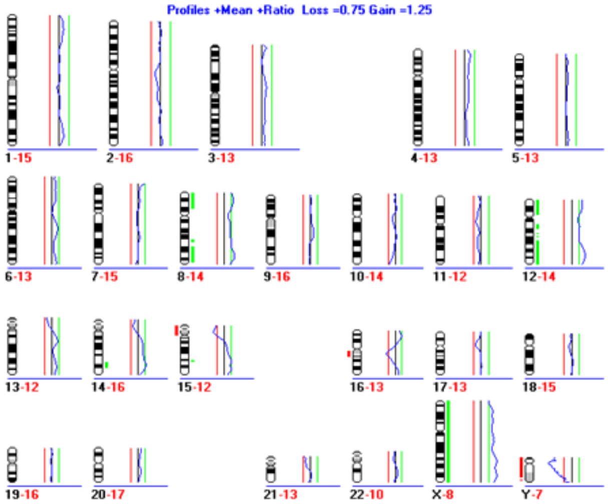

T11 PT1

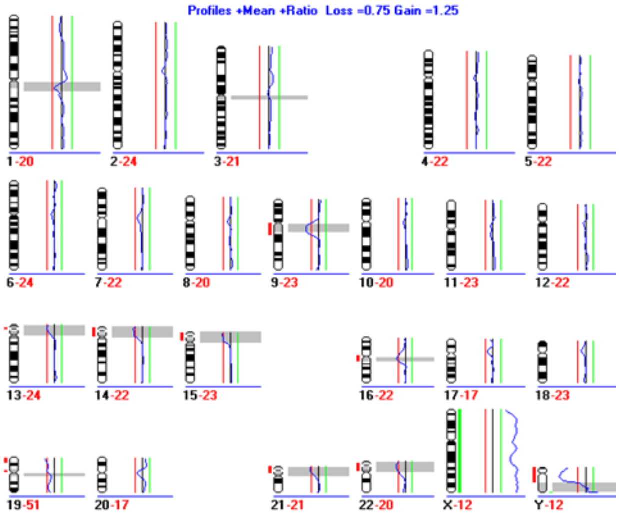

T11 DCC1

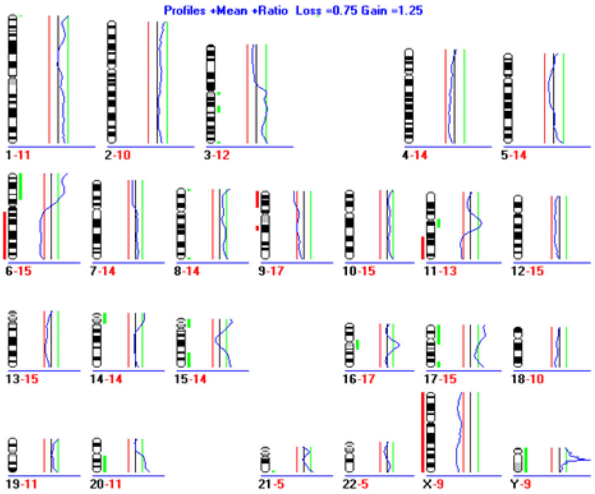

T11 DCC2

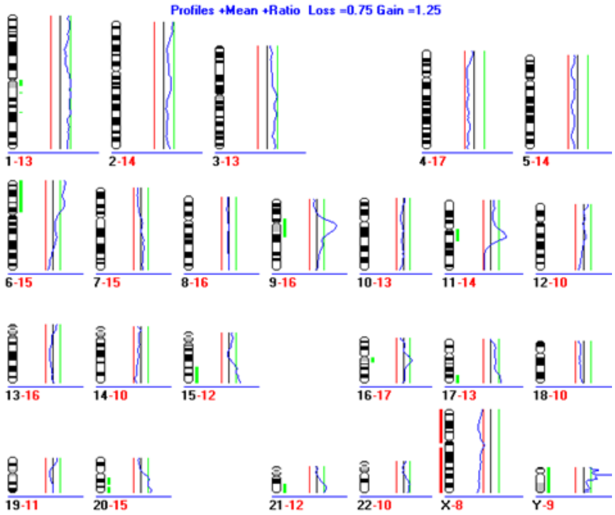

Primary tumour

DCCs

T12 PT1

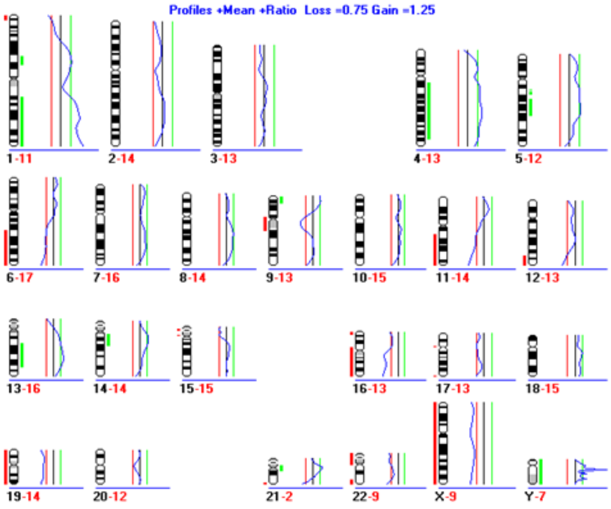

T12 DCC2

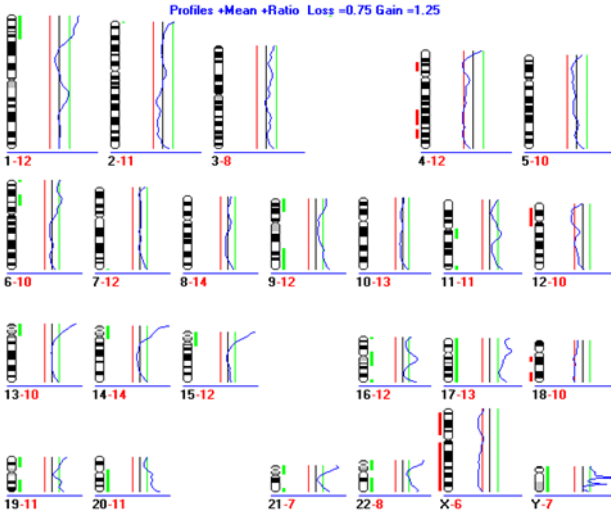

T12 DCC3

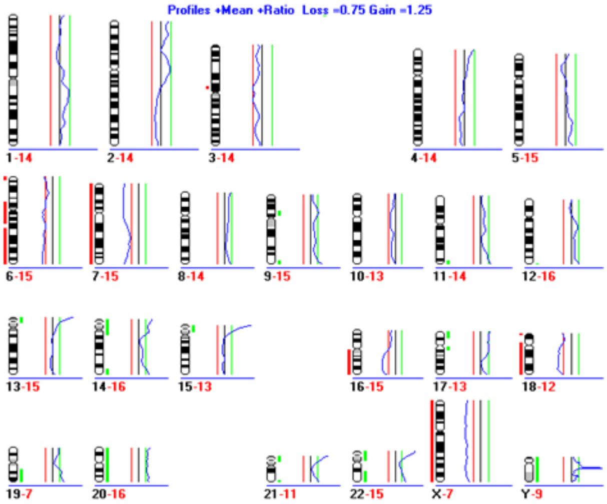

T13 PT1

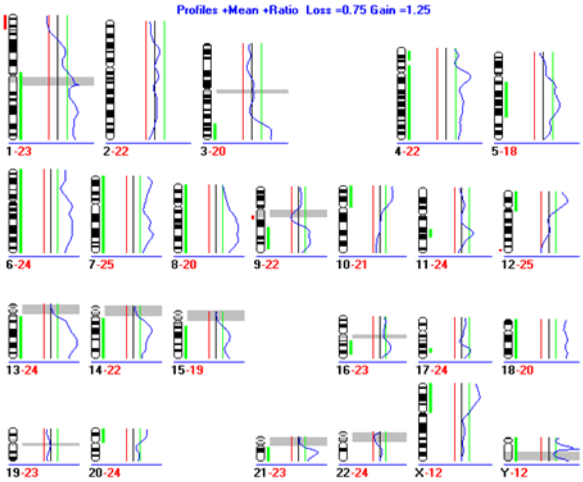

T13 DCC2

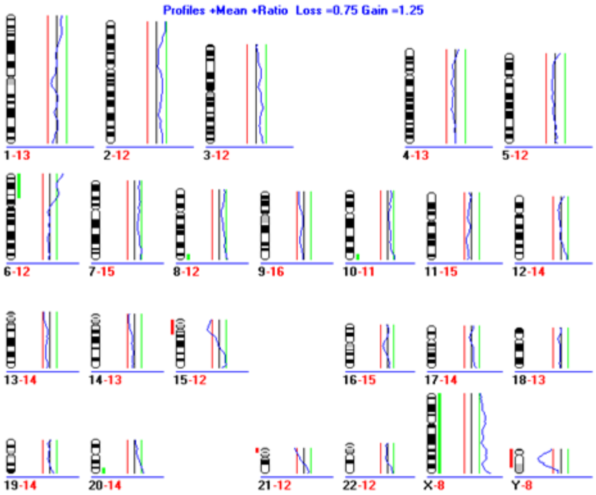

Primary tumour

DCCs

T14 PT1

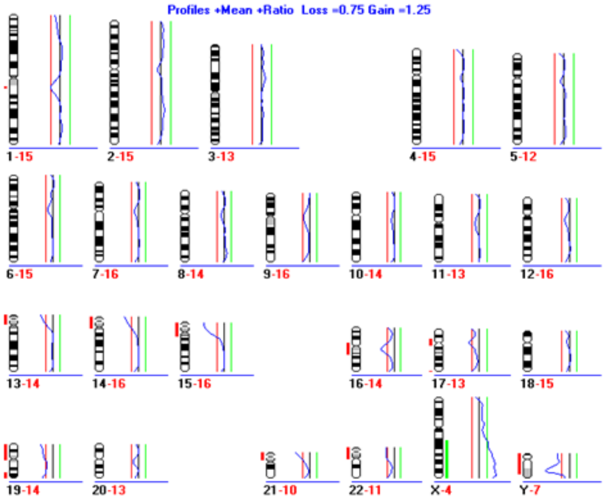

T14 DCC2

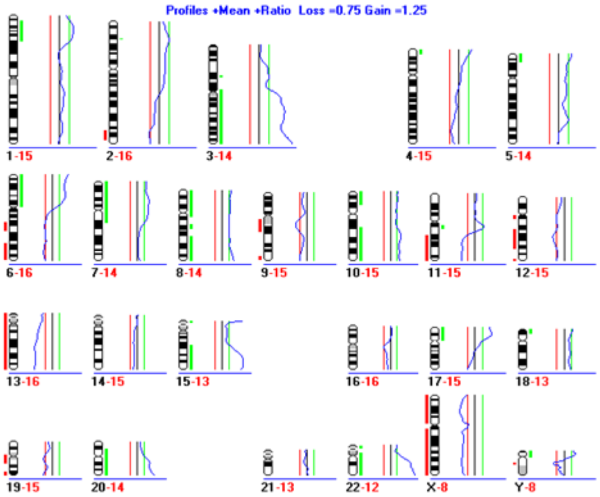

T24 PT1

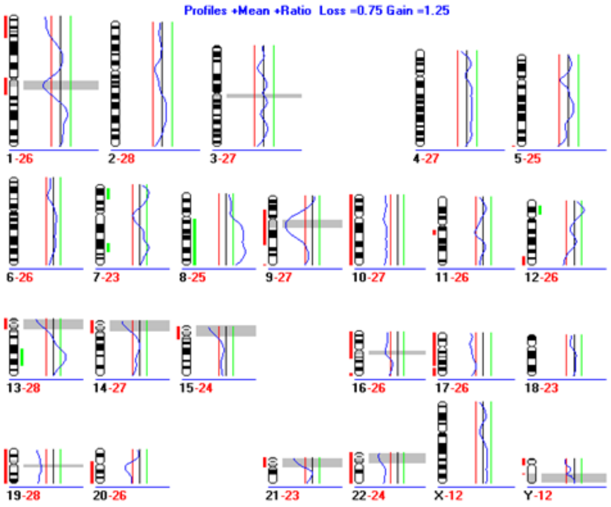

T24 DCC1

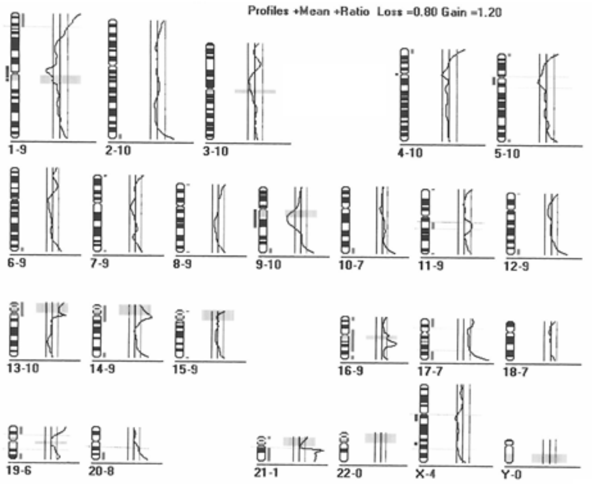

T27 PT2

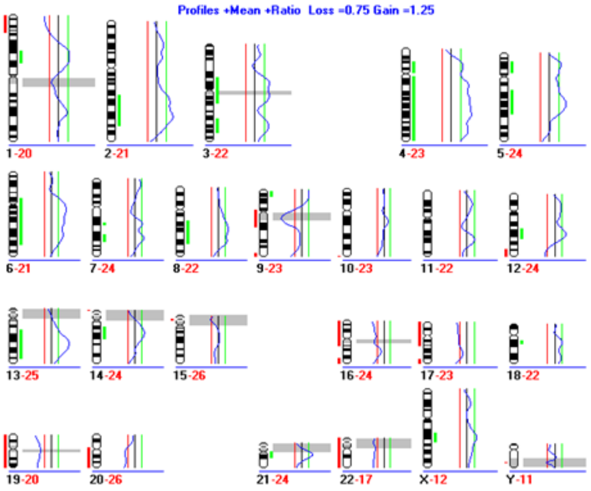

T27 DCC2

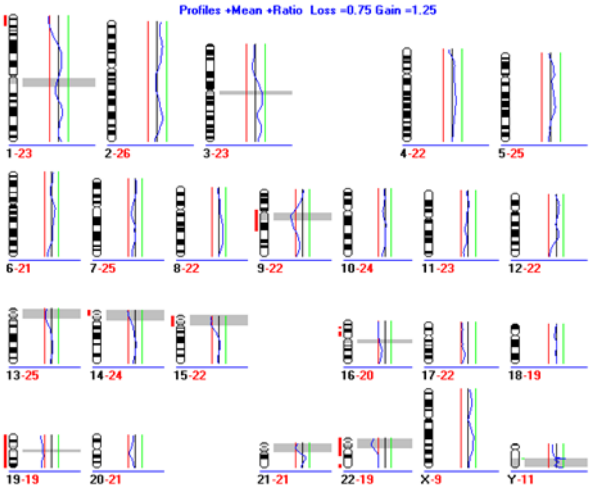

Primary tumour

DCCs

T28 PT1

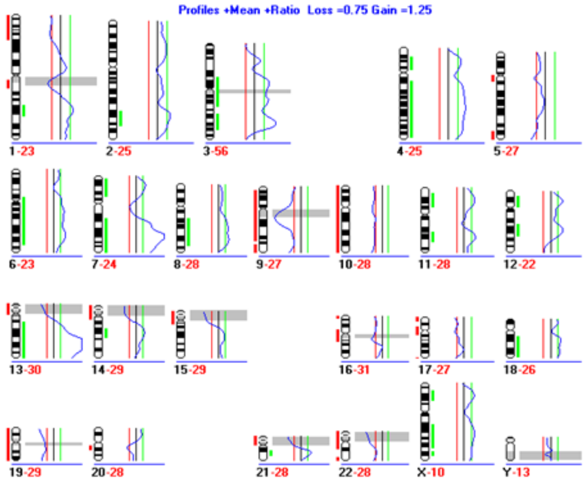

T28 DCC1

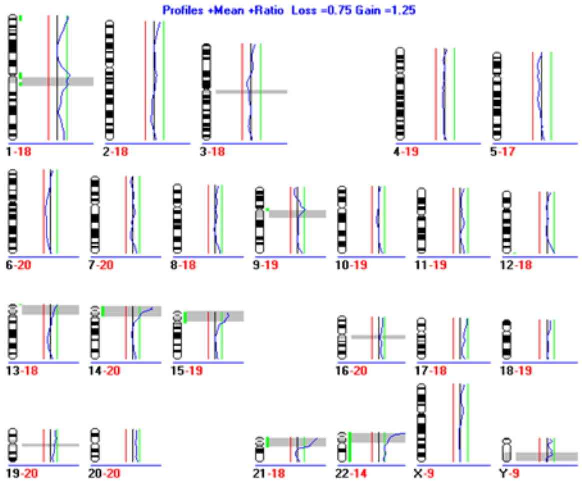

T28 PT2

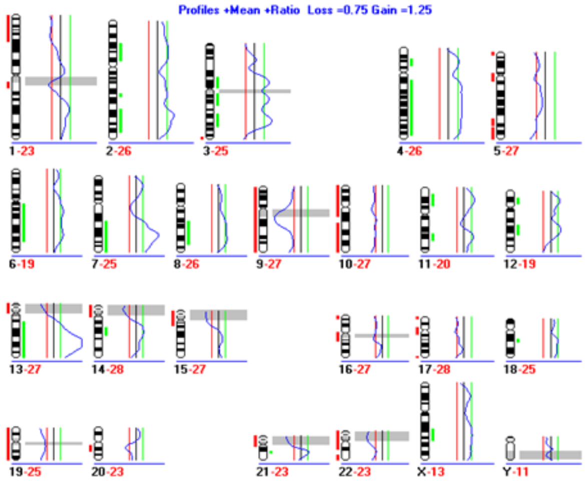

T29 PT1

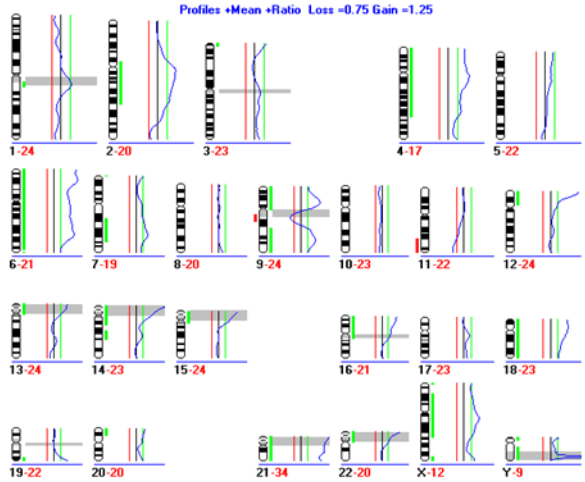

T29 DCC3

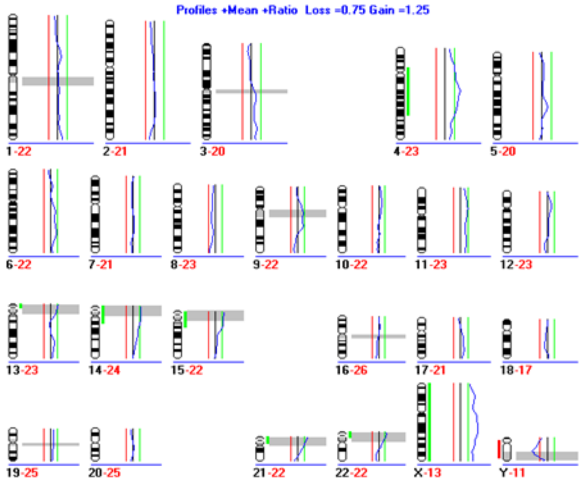

Primary tumour

DCCs

T30 PT2

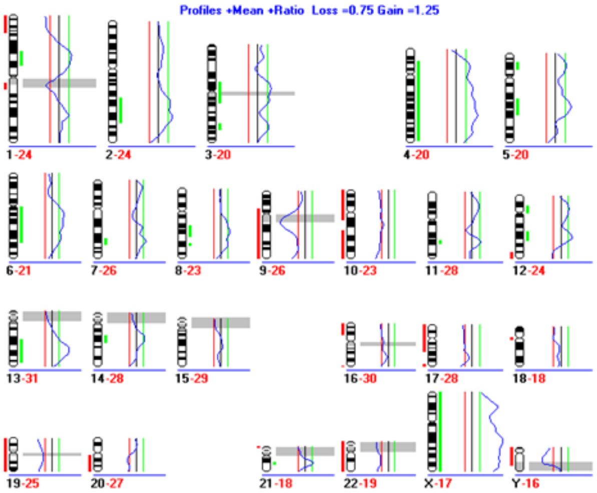

T30 DCC3

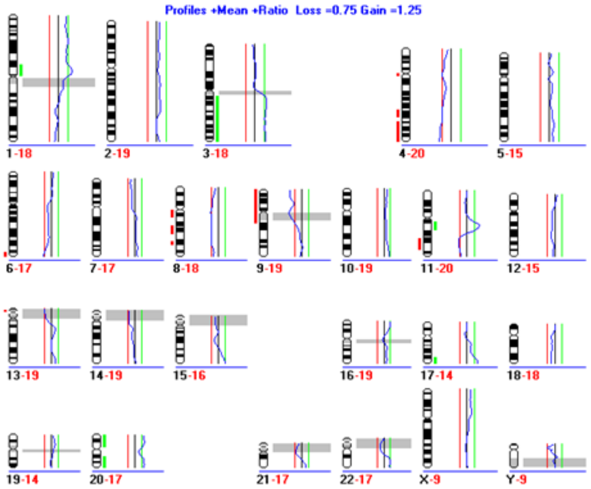

T30 PT3

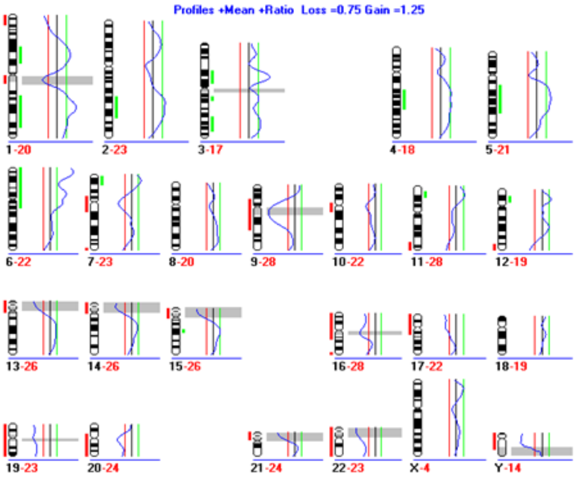

T30 PT4

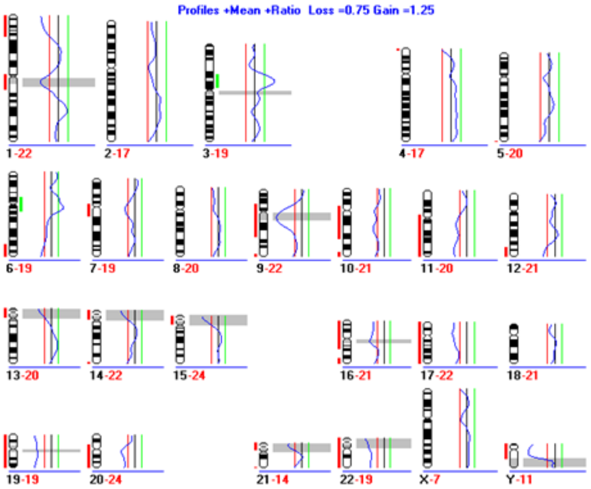

Supplementary Figure 3. CGH profiles of PT/DCC pairs. CGH profiles of 17 patients with PT/DCC pairs. PT and DCCs were microdissected and isolated as gp100-positive cells, respectively and the DNA was amplified. All chromosomes are depicted. In some cases, we used sex-mismatch control DNA to demonstrate successful hybridization. Coloured bars indicate relative gains or losses of the X-chromosome (red, underrepresentation in test cells; green, overrepresentation in test cells) bars next to the ideogram. Chromosomal regions (centromeric regions) marked by gray bars are excluded from analysis as they contain repetitive regions.

a

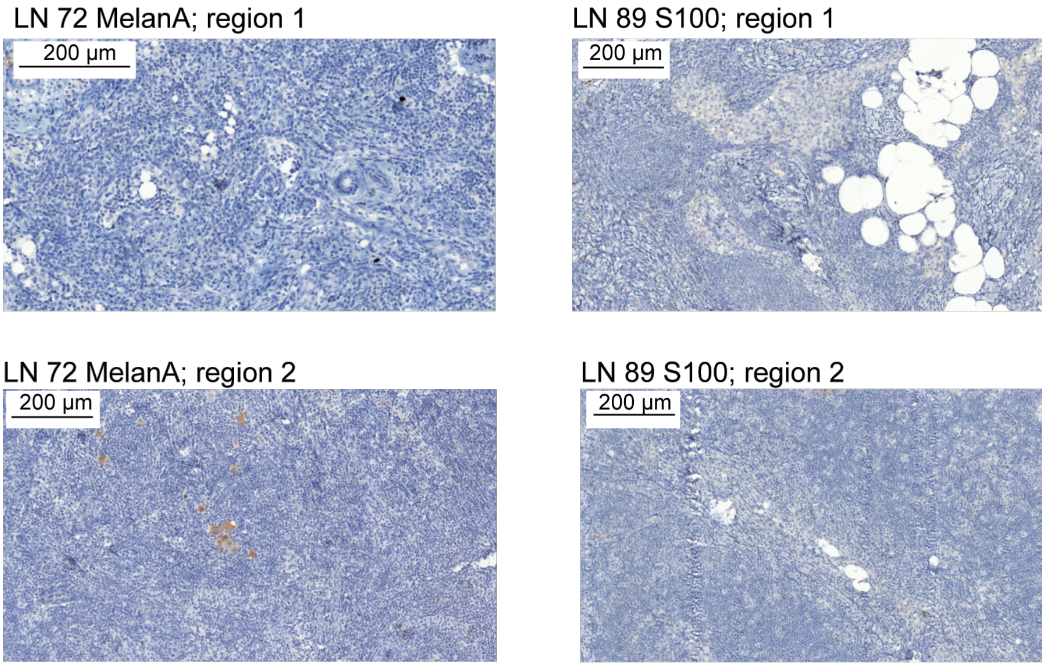

b

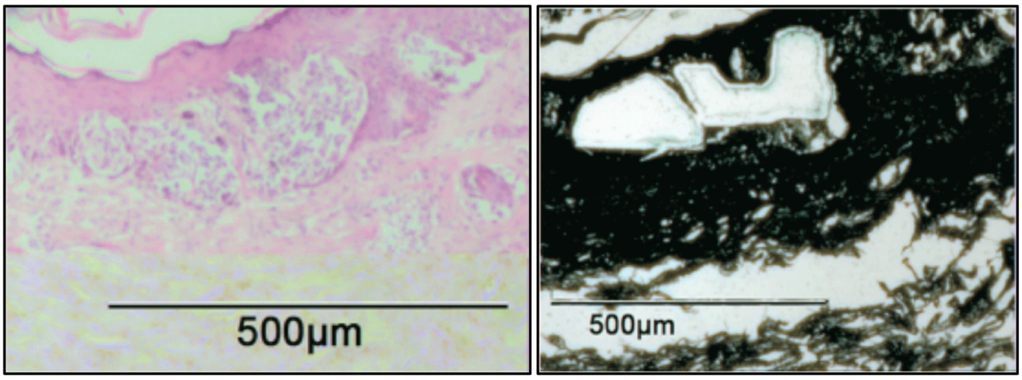

Supplementary Figure 4. DCCs in sentinel lymph nodes and microdissection of FFPE primary melanoma. **(a)** Close-up of central and core regions of lymph node samples with subcapsulary melanoma cell invasion. DCCs enter the lymph node via subcapsulary sinusoid regions. More central regions do not harbour melanoma cells. Selection of region 1 and 2, see main Figure 1b. **(b)** Microdissection of primary melanoma. Left: H&E skin staining containing an area of primary melanoma. Right: Same area after laser-microdissection. Note the excised area of the melanoma.

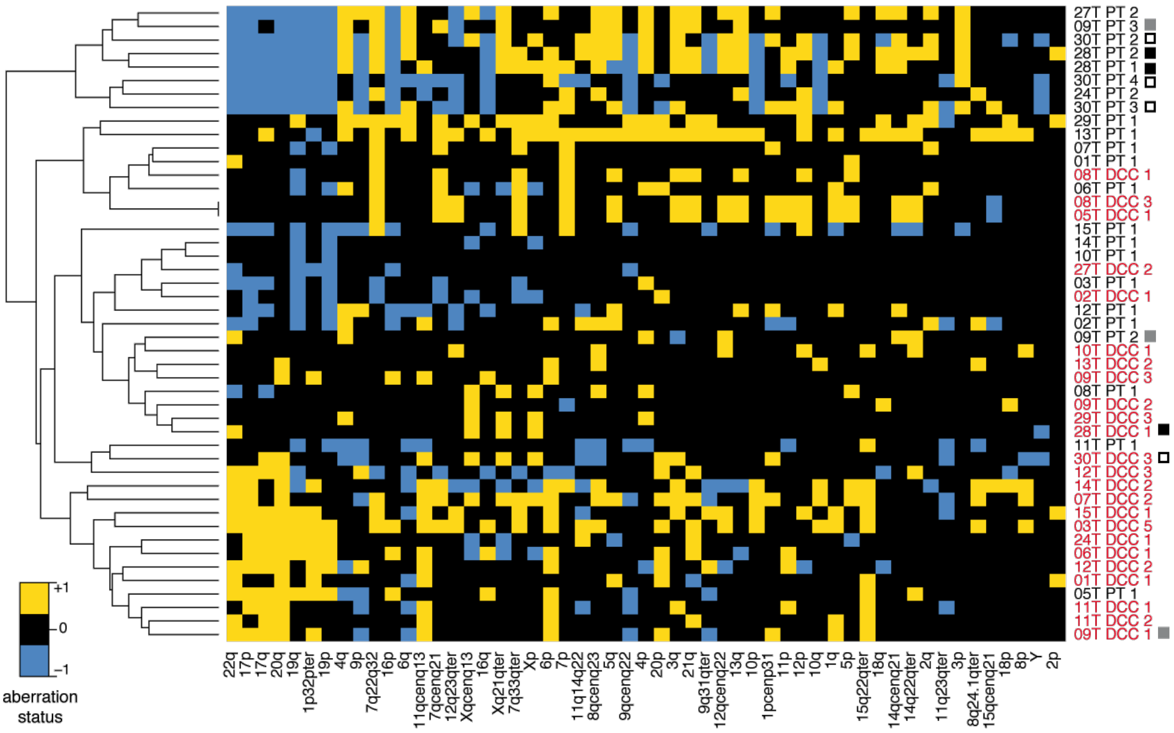

Supplementary Figure 5. Cluster analysis of paired primary tumours and DCC samples. Primary Tumor samples (PT, n=23, 19 patients) and disseminated cancer cells (DCC, n=24, 19 patients) were clustered for chromosomal aberrations (gain = +1; loss = -1). All variable regions are included and ordered according to variability. The identifiers indicate patient ID, sample type and sample index. White (30T), black (28T) and grey (09T) filled squares indicate PT-DCC pairs for which several areas of the primary tumour were available.

Group 1: no branching point

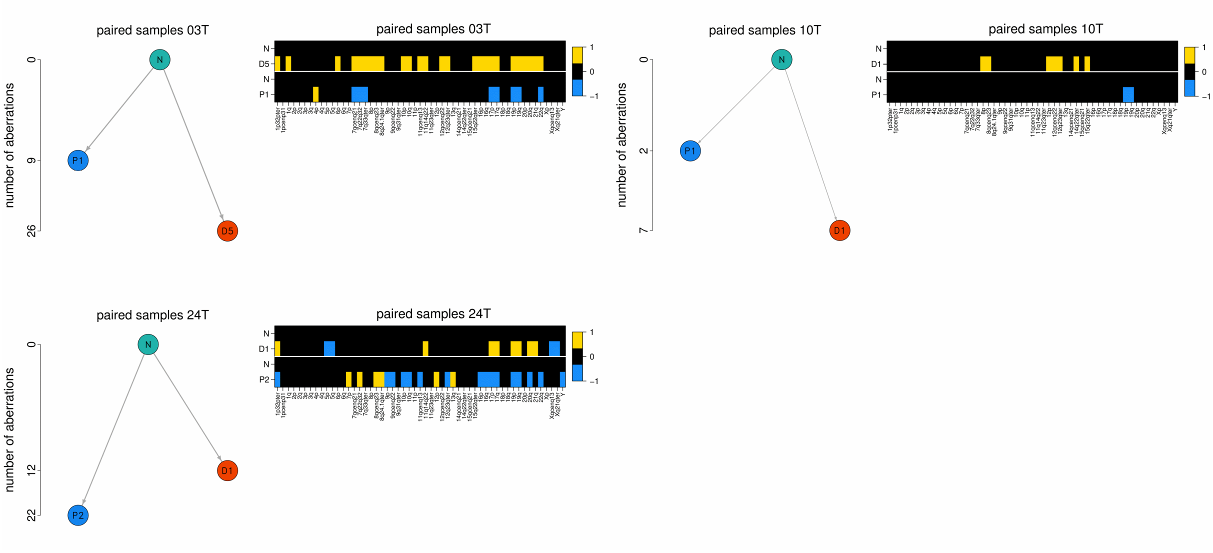

Supplementary Figure 6. Phylogenetic trees for the 3 paired PT-DCC-samples from observed group 1: no branching point. The ordinate of the tree panels indicates the number of aberrations per profile (square root scale). The panels on the respective right sides show aberration profiles along paths from normal cells (N) to primary tumour (P1-2) and DCC (D1-5) samples, respectively. Short sample names correspond to full sample names in Figure 2 and Supplementary Figure 5, e.g. sample ‘P1’ in the first plot for patient ‘03T’ refers to the full sample name ‘03T PT 1’.

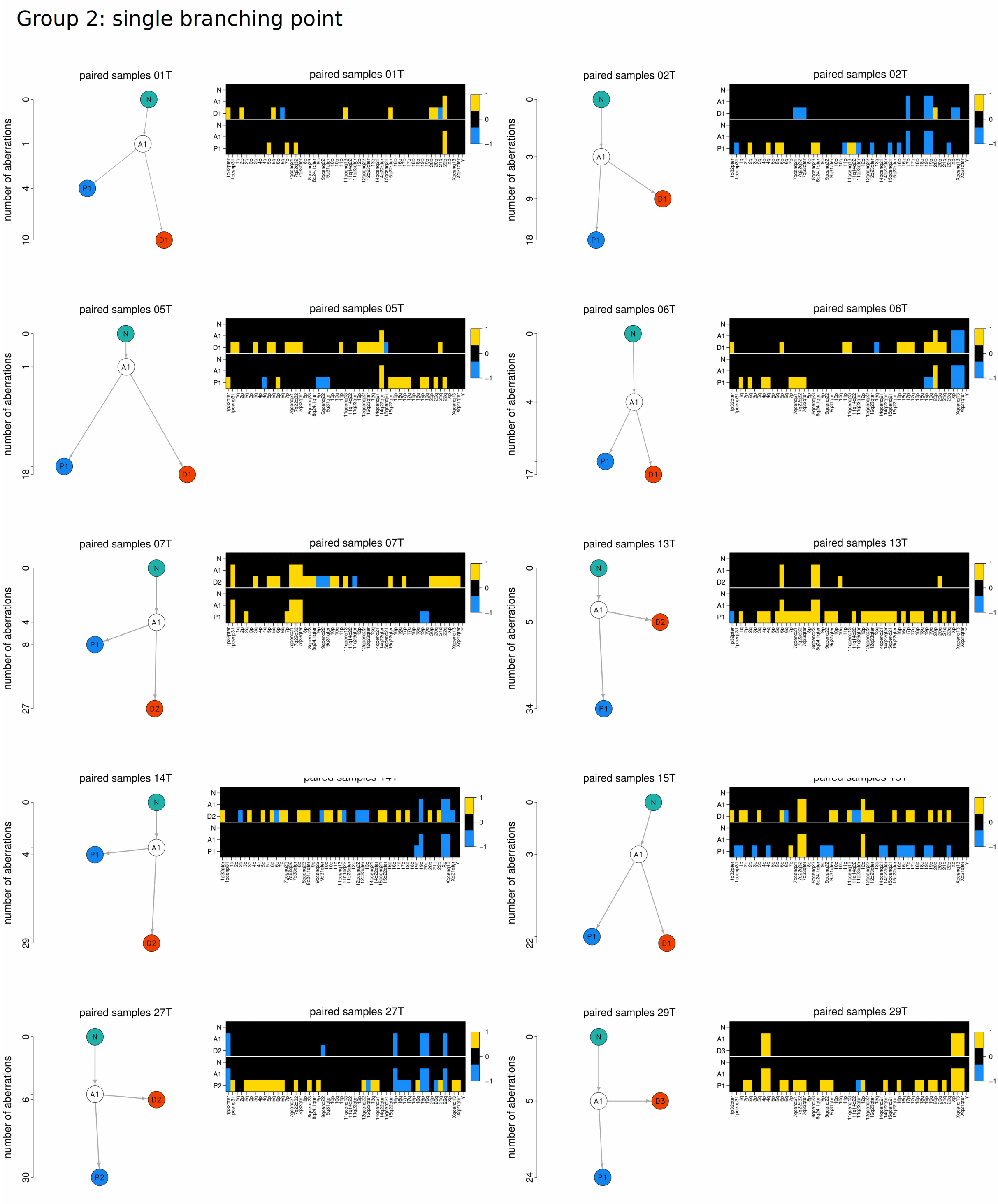

Supplementary Figure 7. Phylogenetic trees for the 10 paired PT-DCC-samples from observed group 2: single branching point. Profiles A1 denote inferred common ancestors (intermediates) (see Methods). For notation see Supplementary Figure 6.

Group 3: multiple branching points

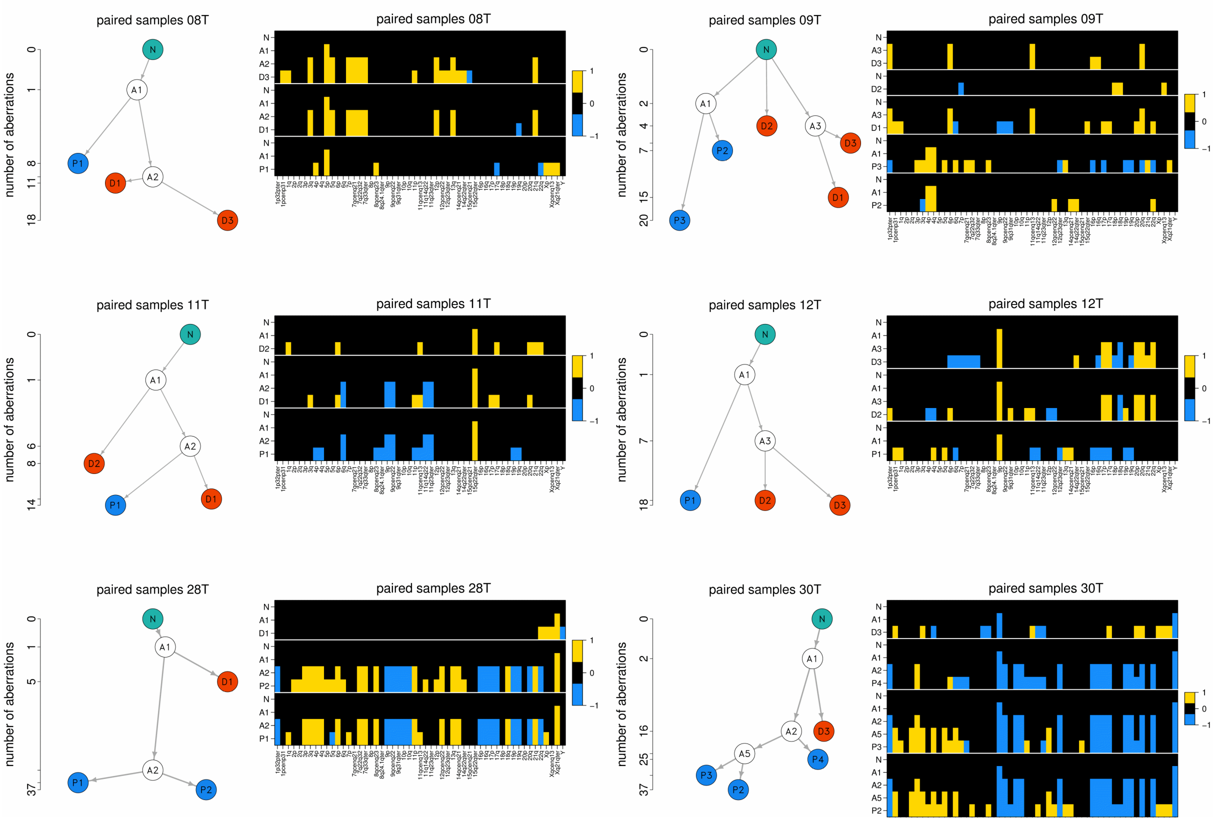

Supplementary Figure 8. Phylogenetic trees for the 6 paired PT-DCC-samples from observed group 3: multiple branching points. For multiple (PT, DCC) samples, acquisition of the same aberration in different branches of the tree was a frequent feature, which notably, would be absent in the commonly used but more restraining Infinite Sites Model (ISM) for clonal trees [2, 3]. Profiles A1-5 denote inferred common ancestors (intermediates) (see Methods). Refer to Supplementary Figure 6 for notation.

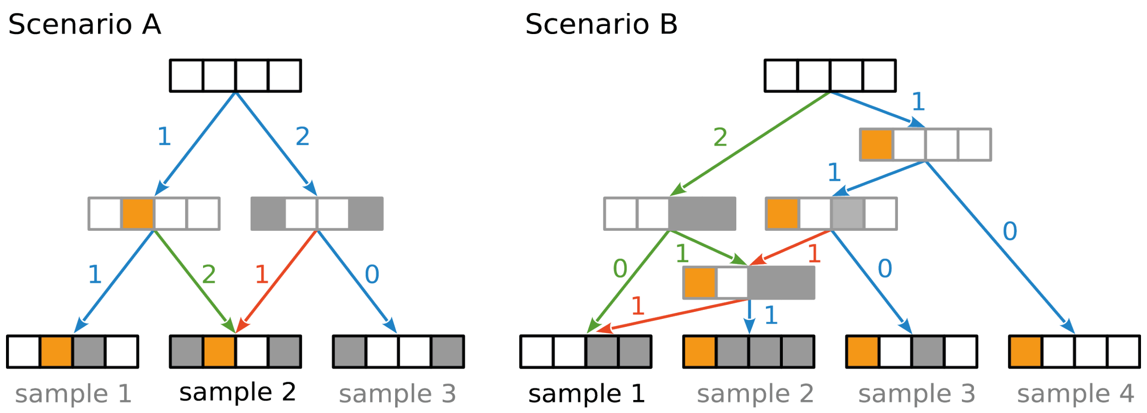

Supplementary Figure 9. Illustration of the phylogeny reconstruction method applied and differences regarding the Infinite Sites Model (ISM). Scenario A: The same alteration can be acquired in different tree branches. The orange alteration in sample 2 can be acquired early (blue edges + green edge, total graph length L = 6) or alternatively late (blue edges + red edge, L = 5). **Scenario B:** Genome evolution can involve reversion of gains. In sample 1 the orange alteration is never acquired (blue + green edges, total graph length L = 6) or re-lost (blue + red edges, back-mutation, L = 5). Note that in both scenarios the graphs that most obviously violate central assumptions of the ISM (i.e. new mutations occur at novel sites (Scenario A) and there is no reversion of alterations (Scenario B)) are shorter and thus favoured by parsimony. Moreover, the sample sets as a whole do not conform to the ISM (no strict inclusion structure, i.e. some samples have non-empty overlap with different branches of the tree) and would be flagged erroneous. Numbers indicate edge lengths (genomic changes). The total graph length L is the sum of the edge lengths. Inferred ancestors are shown with grey edges. Tree roots are normal cells (no alterations).

a

	Low	High	D1	D2	D3
P1	1	1.4	1	0.4	0.2
P2	1.4	2.6	0.8	1	0.8
P3	2.6	3	0.2	0.4	1

b

	Low	High	D1|D1	D1|D2	D1|D3	D2|D1	D2|D2	D2|D3	D3|D1	D3|D2	D3|D3
P1	1	1.4	0.5|0.5	0.6|0.4	0.8|0.2	0.4|0.6	0.2|0.2	0.2|0.1	0.2|0.8	0.1|0.2	0.1|0.1
P2	1.4	2.6	0.4|0.4	0.5|0.5	0.5|0.5	0.5|0.5	0.5|0.5	0.5|0.5	0.5|0.5	0.5|0.5	0.4|0.4
P3	2.6	3	0.1|0.1	0.1|0.2	0.2|0.8	0.2|0.1	0.2|0.2	0.4|0.6	0.8|0.2	0.6|0.4	0.5|0.5

Supplementary Figure 10. Matrix of maximum DCC-like clone fractions. a Clone fraction matrix M for a locus that was measured to be deleted (1), balanced (2) or amplified (3) in the primary tumour (P1,P2,P3) and a single DCC (D1,D2,D3), e.g. if the locus is deleted in the PT (P1) and amplified in the DCC (D3) the matrix element M(P1,D3) (upper right) indicates that the maximum DCC-clone fraction within the PT that is consistent with P1 and D3 is 0.2. The PT can consist of different subclones that have discrete allele frequencies but quasi-continuous clone fractions. Mathematically, these can be pooled into only one unknown clone with continuous allele frequency $C \in [1,3]$. The indicated limiting \`Low´ and \`High´ values specify the range of values that correspond to the respective discrete calls (P1, P2, P3), e.g. all PT mean allele frequencies in the interval [1,1.4] are mapped to the discrete call P1. Matrix M gives the maximum DCC fractions f for which a $C \in [1,3]$ exists so that the mean allele frequency $\bar{P} = (1-f)C + fD$ falls into the corresponding measurement interval [Low,High], e.g. for $f = M(P1, D3) = 0.2$ and $C = 1$ this is fulfilled since $\bar{P} = (1-0.2) \cdot 1 + 0.2 \cdot 3 = 1.4$. If multiple loci are measured, valid fractions across loci must be determined prior to maximization. **b** Clone fraction matrix M for two DCC-like clones $Di_1|Di_2$ and defining equation $\bar{P} = (1 - f_1 - f_2)C + f_1 D_1 + f_2 D_2$. Generally, a continuum of fractions (f_1, f_2) and values C exist for which $\bar{P}$ is within the measured interval, e.g. for P3 and D2|D1 the equation $\bar{P} = (1 - f_1 - f_2)C + f_1 \cdot 2 + f_2 \cdot 1$ has the extremal solutions $\bar{P}_1 = 3$ for $(f_1, f_2) = (0,0)$ and $\bar{P}_2 = 2.6$ for $(f_1, f_2) = (0.2, 0.1)$ using $C = 3$ in both cases. Out of these solutions, M displays fractions with the maximum product $f_1 \cdot f_2$. Evidently, if multiple loci were measured, valid fractions across loci must be determined before product maximization can be performed (see also Methods).

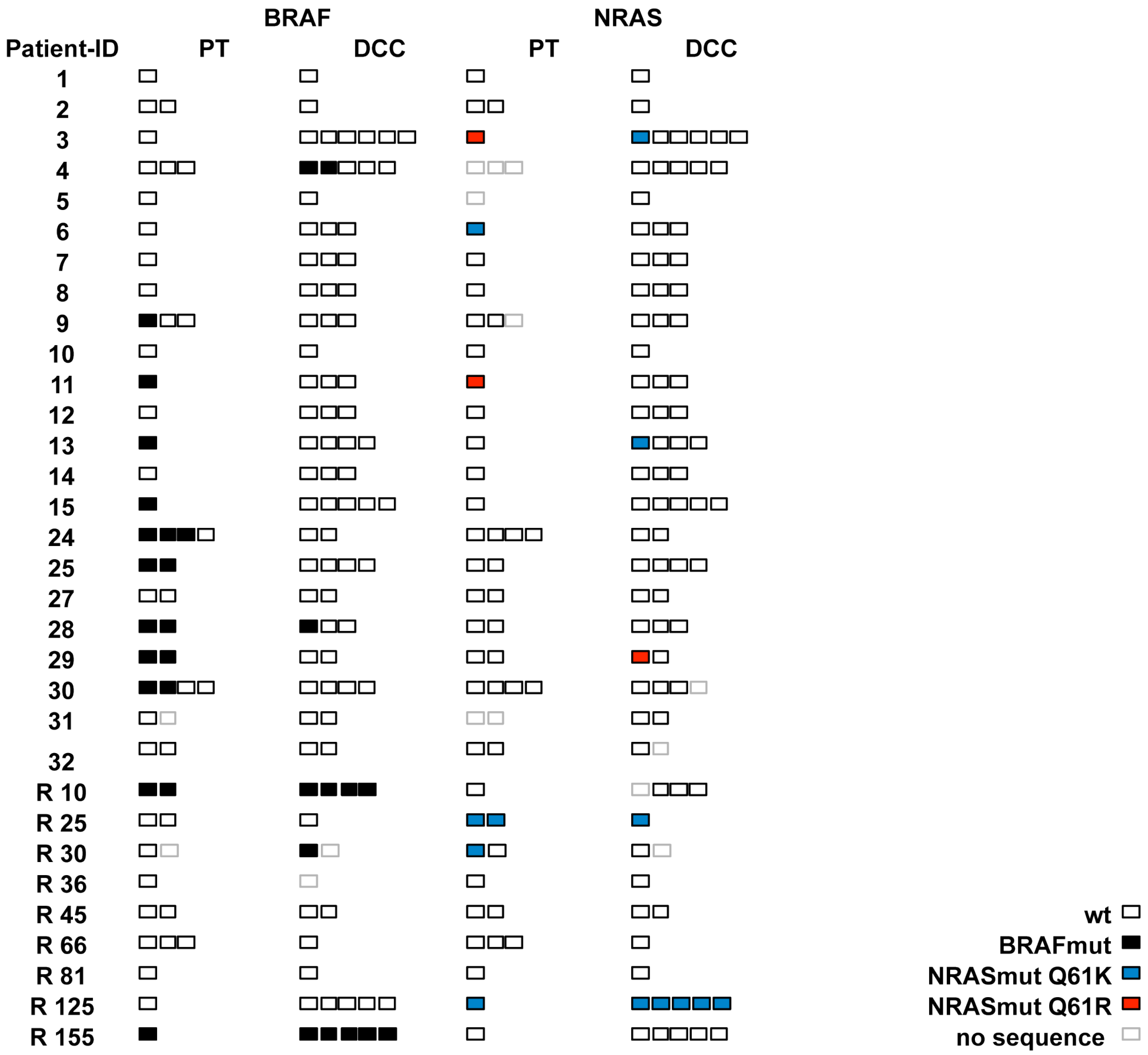

Supplementary Figure 11. Oncogenic mutations in *BRAF* and *NRAS* of paired primary tumours and DCCs. Squares indicate areas (PT, 32 patients) or individual cells (DCCs, 32 patients). Black filled squares indicate that the mutation was detected and white filled squares indicate wild type sequence. Blue and red filled squares indicate *NRAS*mut Q61K and *NRAS*mut Q61R, respectively. Grey lined squares indicate samples of which no sequence could be obtained.

a

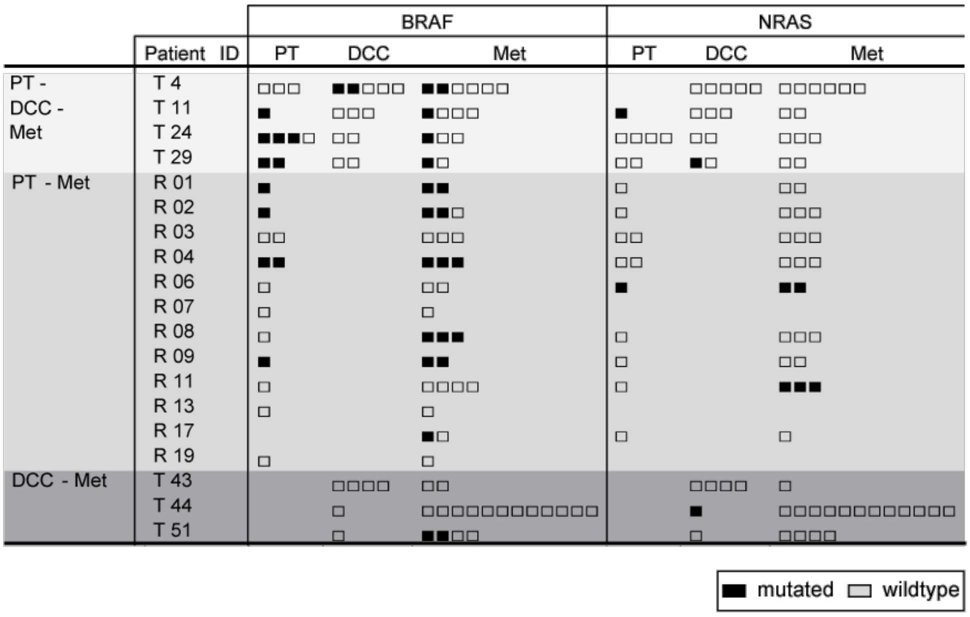

b

patient	date PT-surgery	date Met-surgery	therapy until Met-surgery
R1	04/2008	04/2008 + 05/2008	none
R2	06/2008	06/2008+10/2007+12/2008	none
R3	07/2008	12/2008 + 2009	none
R4	10/2007	06/2009	08/2008-11/2008: 4 cycles of Dacarbazin
R6	2007*	06/2007 + 02/2009	none
R7	12/2005	09/2009	none
R8	1999	07/2009 + 11/2009	none
R9	11/2006	12/2006	none
R11	1997	05/2003+ 07/2004	none
R13	06/2006	03/2010	none
R17	07/2005	2005	none
R19**	09/2009	08/2009	none
T4	10/2003	07/2006	intradermal experimental vaccination and Dacarbazin
T11	05/2004	03/2006	none
T24	08/1999	03/2003	none
T29	07/2003	01/2004	low dose IFN alpha
T43	01/05/2004	15/05/2004	none
T44	05/2004	01/2006	none
T51	10/2003	04/2005	low dose IFN alpha

* surgery outside of hospital, metastasis formed after primary tumour resection
** metastasis was surgically removed before primary tumour

Supplementary Figure 12. Oncogenic mutations in *BRAF* and *NRAS* of paired PT-DCC-metastases triplets (n=4), pairs of PT-metastases (n=12) or pairs of DCC-metastases (n=3). (a) Squares indicate areas (PT and metastases) or individual cells (DCCs). Black squares indicate mutation was detected and white squares indicate wild type sequence. **(b)** Patient information: date of primary tumour and metastasis resection. Therapy that was applied between primary tumour resection and resection of metastasis is indicated.

a

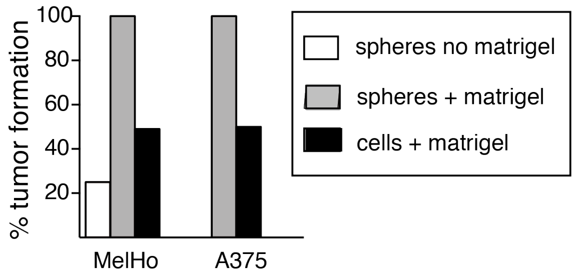

b

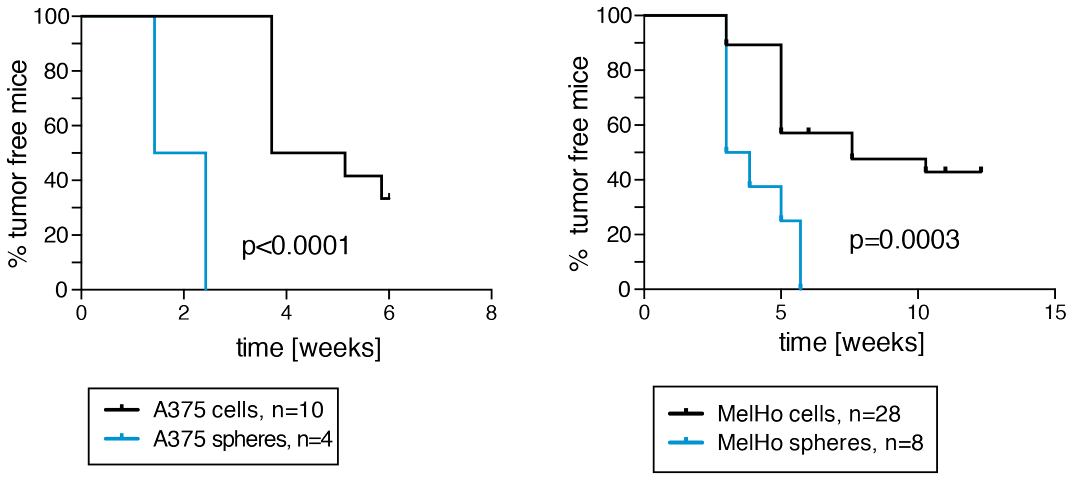

c

patient ID	sample	TH01	D21S11	D5S818	D13S317	D16S538	vWA
LN125	DCC	7	29, 30	10,13	11,12	9,13	11,14,21
	PDX	7	29, 30	10,13	11,12	9,13	11,14
LN135	DCC	8,9	29,30,32.2,33.2	12,13	9,11	12,13	12,14,15
	PDX	8,9	29,30,32.2,33.2	13	9,11	12,13	12,14,15
LN154	DCC	7,9.3	28,29	11,12	11,12	n.d.	12,13,17,21
	sphere	7,9.3	28,29,30.2,31.2	11,12	11,12	11,12	13,17,21
	PDX	n.d.	n.d.	n.d.	n.d.	n.d.	n.d.
LN155	DCC	7,9	29,32.2	12,13	11,12	9,10,11	14,17,21
	sphere	7,9	29,32.2	12,13	11,12	9,10,11	14,17,21
	PDX	7,9	29,32.2	12,13	11,12	9,10,11	14,17,21

Supplementary Figure 13: Xenotransplantation of single cells and spheres from melanoma cell lines. (a) Groups of 3-5 spheres with or without matrigel or groups of 5 single cells with matrigel were s.c. transplanted into NSG-mice. The percentage of injection sites with tumour growth was determined. Mel-Ho: 4-28 injcetions, A375: 4-16 injections. **(b)** Mice with s.c. injections of groups of 3-5 spheres plus matrigel or groups of 5 single cells plus matrigel were weekly palpated and the time-point of first palpation of the s.c. growing tumour was documented. p-values indicate statistical significance (log-rank test). Number of injection sites are given in the figures. **(c)** Confirmation of patient-origin of spheres/xenografts by STR analysis for 4/6 xenografts. LN154 was erroneously fixed and could not be analysed thereafter. Tri- as well as quatro-allelic patterns at D21S11, D16S538 and vWA loci indicate repeat number at these loci. n.d. = not detectable

	number of patients	percentage [%]	median	range	interquartile range
**gender**					
female	25	41			
male	36	59			
**age [years]**			61	20 - 78	47.5 - 70
**Breslow`s thickness** [mm]			2.35	0.6 - 10.0	1.3 - 4.08
**Ulceration**					
no	39	63.9			
yes	19	31.2			
not specified	3	4.9			
**localization**					
extremities	32	52.5			
trunk or head	29	47.5			
**nodal status histopathology**					
negative	37	60.7			
positive	24	39.3			
**DCCD**			32	1 - 800000	3 - 177
**clinical stage**					
IA	4	6.5			
IB	15	24.6			
IIA	7	11.5			
IIB	5	8.2			
IIC	2	3.3			
IIIA	11	18.0			
IIIB	13	21.3			
IIIC	4	6.5			

Supplementary Table 1. Baseline characteristics of melanoma patients. The number of patients with clinical stage IA to IIC is lower than the number of lymph node negative patients (33 vs. 37), because four patients with histopathologically negative sentinel lymph nodes were staged IIIB due to satellite metastasis of the skin or intralymphatic metastases (N2c).

Category	Cell Division / Risk Type 'Clock'	Angle Type	Positive Change		Negative Change	
			Del (+D)	Amp (+A)	Del (-D)	Amp (-A)
1	n_T	135	percental increase with n_T (+D1)	percental increase with n_T (+A1)	percental die out with n_T (-D1)	percental normalisation or die out with n_T (-A1)
2	n_T+n_L	90	percental increase with n_T+n_L (+D2)	percental increase with n_T+n_L (+A2)	percental die out with n_T+n_L (-D2)	percental normalisation or die out with n_T+n_L (-A2)
3	n_T+2n_L	45	percental increase with n_T+2n_L (+D3)	percental increase with n_T+2n_L (+A3)	percental die out with n_T+2n_L (-D3)	percental normalisation or die out with n_T+2n_L (-A3)
4	n_L	0	percental increase with n_L (+D4)	percental increase with n_L (+A4)	percental die out with n_L (-D4)	percental normalisation or die out with n_L (-A4)

Supplementary Table 2: Classifier categories. Each classifier category (1-4) corresponds to a risk function *E* (Cell Division / Risk Type, 'Clock') and prototypic angle (Angle Type). When applied to a specific locus/mutation, each classifier divides the experimental samples into two classes (sample populations) denoted by class1 and class2. Each class has a certain percentage of samples in which the locus is deleted, balanced or amplified. These percentages may experience positive or negative changes when going from class2 to class1, e.g. if in class2 10% of samples carry a deletion while in class1 this applies to 30%, the percental change is +20%. Columns 4-5 list positive changes for amplifications (+A) and deletions (+D), while columns 6-7 list the corresponding negative changes (-A,-D). Since class1 always corresponds to a higher number of cell divisions of the corresponding risk type (column 2) as compared to class2, positive changes (+A,+D) correspond to an increased risk of acquiring alterations through cell division. Conversely, negative changes (-A,-D) correspond to genetic normalisation (re-loss) of amplifications (-A) or die out of cells that carry amplifications (-A) or deletions (-D), assuming that the re-gain of deletions can be neglected in practical terms. Changes are numbered by the corresponding type, e.g. +A3 indicates a positive change in the percentage of amplifications according to category 3. The effect of stopped dissemination is equivalent to a die out in the primary thus always conforming to category 1.

	Chr Band	FDR	Angle	Angle Type	Change [%]		Change Type		Gene Symbol
					Del	Amp	Del	Amp	
Category 1: n_T	12q11-q13	0.047	141	135	+20	+14	+D1	+A1	ARID2, ATF1, ERBB3, SMARCD1, STAT6
	9p11.1-p13	0.004	138		-41	-1	[+D] –D1	[+A] –A1	FANCG, PAX5
	18q21-q23	0.003	134		+37	+8	+D1	+A1	BCL2, KDSR, MALT1, SETBP1, SMAD2, SMAD4
	13q11-q22	0.014	133		+20	+12			BRCA2, CDX2, FLT3, FOXO1, LCP1, LHFP, RB1, ZNF198
	13q31-q34	0.014	133		+16	+12			ERCC5
	19p11-p13.3	0.037	132		+8	+25			BRD4, CALR, CRTC1, DNAJB1, DNM2, ELL, FSTL3, GNA11, JAK3, KEAP1, LYL1, MAP2K2, MLLT1, PRKACA, SH3GL1, SMARCA4, STK11, TCF3, TPM4
	3q11.1-q21	0.05	128		+16	+40			CBLB, CNBP, TFG
	19q11-q13.1	0.008	123		+12	+26			AKT2, BCL3, CCNE1, CEBPA, TFPT
Category 2: n_T+n_L	1p11-p13	0.041	89	90	+5	-23	+D2	[+A] –A2	FAM46C, NOTCH2, RBM15, TRIM33
	17q25	0.033	85		0	-39			ASPSCR1, CANT1, CLTC, H3F3B, RNF213, SEPT9, SRSF2
	17q22-q24	0.008	79		0	-38			BCL5, BRCA1, BRIP1, CD79B, CLTC, COL1A1, DDX5, ETV4, HLF, MLLT6, MSI2, PPM1D, PRKAR1A, RNF43
	1p34.1-p36.3	0.028	79		+4	-39	+D2		ARID1A, CAMTA1, CSF3R, LCK, MDS2, MPL, MTOR, MUTYH, MYCL, PAX7, PRDM16, SDHB, SFPQ, SPEN, THRAP3
	1p21-p33	0.002	74		+13	-40			ATP1A1, BCL10, CDKN2C, EPS15, JAK1, JUN, MUTYH, PRDM16, RPL5, STIL, TAL1
Category 3: n_T+2n_L	**BRAFmut**	0.0005	38	45		+45		+A3	**BRAF**
	11q11-q13	0.007	38		-5	-31	[+D] –D3	[+A] –A3	CCND1, CLP1, MEN1, NUMA1, SDHAF2
	6p11.1-p12	0.007	38		+2	-32	+D3		HSP90AB1
	6p21.1-p25	0.014	33		+2	-35			CCND3, DAXX, DEK, FANCE, HIST1H3B, HIST1H4I, HLA-A, HMGA1, IRF4, NFKBIE, PIM1, POU5F1, SRSF3, TFEB, TRIM27
	15q23-q26	0.049	28		+2	-33			NTRK3, PML
Category 4: n_L	10p11.1-p15	0.015	6	0	+23	-12	+D4	[+A] –A4	ABI1, GATA3, KIF5B, KLF6, MLLT10
	3q22-q29	0.03	4		+8	-26			ATR, BCL6, CNBP, EIF4A2, ETV5, FOXL2, GMPS, LPP, MAP3K13, MECOM, MLF1, PIK3CA, RPN1, SOX2, TBL1XR1, TFRC, TP63, WWTR1
	**9p21-p24**	0.022	0		+41	-1			CD274, **CDKN2A, CDKN2A(p14)**, JAK2, MLLT3, PSIP1
	10q23-q26	0.035	0		+12	-22			FAS, FGFR2, KIAA1598, NFKB2, NT5C2, NUTM2A, PRF1, PTEN, SUFU, TCF7L2, TLX1, VTI1A
	Xq25-q28	0.041	0		-25	-29	[+D] -D4		ATP2B3, BCORL1, ELF4, GPC3, MTCP1, PHF6, RPL10, SEPT6, STAG2
	9q11.1-q13	0.03	-2		+43	-6	+D4		
	Yq11.1-q11.2	0.03	-2		+29	+16		+A4	
	9q34-qter	0.005	-4		+3	-49		[+A] –A4	ABL1, BRD3, NOTCH1, NUP214, RALGDS, SET, TSC1
	**7q21-q36**	0.011	-8		-10	+32	[+D] -D4	+A4	AKAP9, BRAF, CDK6, CREB3L2, CUX1, EZH2, FAM131B, KIAA1549, **MET**, MNX1, POT1, SMO, SND1, TRB, TRIM24, TRRAP
	9q21-q33	0.023	-8		+29	-9	+D4	[+A] –A4	FANCC, FNBP1, GNAQ, KLF4, NR4A3, OMD, PTCH1, SYK, TAL2, XPA

Supplementary Table 3: Characteristics of best classifiers for different loci. Chromosomal band (Chr Band), FDR-corrected p-value (FDR), best classifier angle (Angle), prototypic angle (Angle Type), change in the percentage of deletions (Change Del) and amplifications (Change Amp), the corresponding type of change (Change Type; Supplementary Table 1) and the cancer genes (Gene Symbol) located within the respective chromosomal band (according to the COSMIC cancer gene census v80). Negative percental changes (-A,-D) must be preceded by a corresponding acquisition (+A,+D) occurring early during tumour/colony development before sample acquisition (thickness < 0.6 mm). Early acquisition is indicated by a prefixed [+A] or [+D] without type label (unknown). As an example, for 9p11.1-13 the entry '[+D] –D1' indicates that deletions were acquired early, while later cells carrying the deletion died out in the primary (nutritional change, clonal competition, etc.) or stopped disseminating while surviving in the lymph node. Generally, loci in category 1 acquired alterations with increasing number of cell divisions (7/8 cases) while loci in categories 2 and 3 lost amplifications (9/9 cases) and gained deletions (6/9 cases). BRAF mutations are treated as amplifications and increase with an increasing number of cell divisions (category 3). The picture for category 4 is slightly more heterogeneous but follows the same trend: gain of deletions (8/10 cases) and loss of amplifications (8/10 cases).

References

1. Polzer B, *et al.* Molecular profiling of single circulating tumor cells with diagnostic intention. *EMBO molecular medicine* **6**, 1371-1386 (2014).

2. Jahn K, Kuipers J, Beerenwinkel N. Tree inference for single-cell data. *Genome biology* **17**, 86 (2016).

3. Ross EM, Markowetz F. OncoNEM: inferring tumor evolution from single-cell sequencing data. *Genome biology* **17**, 69 (2016).
